# The Blood Exposome and Its Role in Discovering Causes of Disease

**DOI:** 10.1289/ehp.1308015

**Published:** 2014-03-21

**Authors:** Stephen M. Rappaport, Dinesh K. Barupal, David Wishart, Paolo Vineis, Augustin Scalbert

**Affiliations:** 1 Center for Exposure Biology, School of Public Health, University of California, Berkeley, Berkeley, California, USA; 2 Nutrition and Metabolism Section, Biomarkers Group, International Agency for Research on Cancer, Lyon, France; 3 Department of Biological Sciences, University of Alberta, Edmonton, Alberta, Canada; 4 MRC-PHE (Medical Research Council–Public Health England) Centre for Environment and Health, School of Public Health, Imperial College, London United Kingdom; 5 HuGeF (Human Genetics Foundation), Torino, Italy

## Abstract

Background: Since 2001, researchers have examined the human genome (G) mainly to discover causes of disease, despite evidence that G explains relatively little risk. We posit that unexplained disease risks are caused by the exposome (E; representing all exposures) and G × E interactions. Thus, etiologic research has been hampered by scientists’ continuing reliance on low-tech methods to characterize E compared with high-tech omics for characterizing G.

Objectives: Because exposures are inherently chemical in nature and arise from both endogenous and exogenous sources, blood specimens can be used to characterize exposomes. To explore the “blood exposome” and its connection to disease, we sought human blood concentrations of many chemicals, along with their sources, evidence of chronic-disease risks, and numbers of metabolic pathways.

Methods: From the literature we obtained human blood concentrations of 1,561 small molecules and metals derived from foods, drugs, pollutants, and endogenous processes. We mapped chemical similarities after weighting by blood concentrations, disease-risk citations, and numbers of human metabolic pathways.

Results: Blood concentrations spanned 11 orders of magnitude and were indistinguishable for endogenous and food chemicals and drugs, whereas those of pollutants were 1,000 times lower. Chemical similarities mapped by disease risks were equally distributed by source categories, but those mapped by metabolic pathways were dominated by endogenous molecules and essential nutrients.

Conclusions: For studies of disease etiology, the complexity of human exposures motivates characterization of the blood exposome, which includes all biologically active chemicals. Because most small molecules in blood are not human metabolites, investigations of causal pathways should expand beyond the endogenous metabolome.

Citation: Rappaport SM, Barupal DK, Wishart D, Vineis P, Scalbert A. 2014. The blood exposome and its role in discovering causes of disease. Environ Health Perspect 122:769–774; http://dx.doi.org/10.1289/ehp.1308015

## Introduction

Worldwide mortality is dominated by noncommunicable diseases, particularly cardiovascular disease (29%), cancer (15%), and respiratory diseases (7%) (Lozano et al. 2012). These chronic diseases result from the combined effects of the human genome (G) and exposome (E; representing all exposures). (Although geneticists use the term “environment” to denote nongenetic factors, many scientists and the general public equate “environment” with “pollution,” which represents only one class of exposures. We use the term “exposome” to encompass all exogenous and endogenous exposures.) But attribution of risks to G and E and their interaction (G × E) has been problematic because of disparities in characterizing genes and exposures (Rappaport and Smith 2010; Wild 2005). In fact, sequencing the human genome in 2001 permitted researchers to comprehensively explore G and its progeny (i.e., genome → transcriptome → proteome → metabolome) but did not promote detailed characterization of E, which in epidemiological and clinical research still relies on questionnaires, geographical information, and targeted surveys (Ezzati and Riboli 2013; Lim et al. 2012). In addition, the study of external and internal exposures (including endogenous chemicals) has focused on a limited number of molecules and metals that cannot compare with the resolution of genome-wide association studies (GWAS).

**Figure 1:**
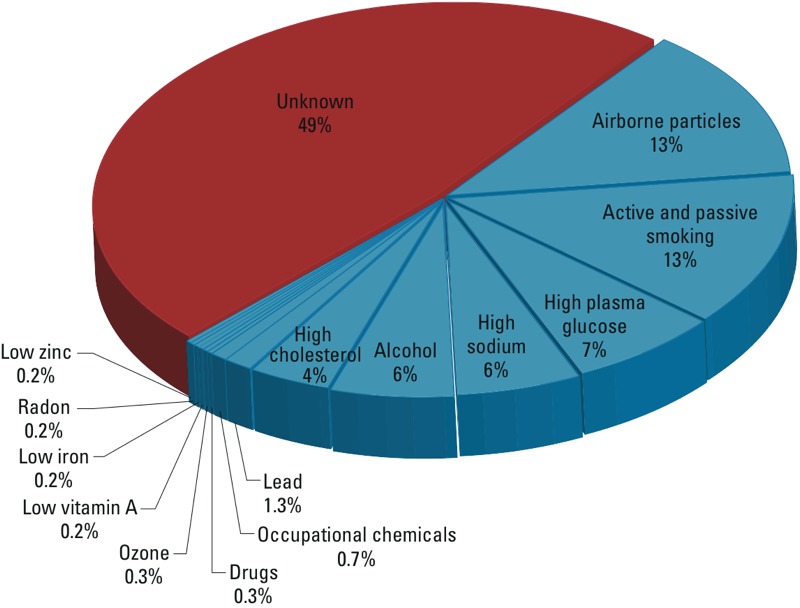
Risk factors for exposures that contribute to chronic-disease mortality. The chart was compiled from World Health Organization estimates of exposures affecting 50 million global deaths in 2010 (Lim et al. 2012). (Because some risk factors may be correlated, the indicated percentages are approximate.)

Interestingly, the variation in chronic-disease incidence explained by scores of GWAS has been so small that searches are under way for “missing heritability” (Goldstein 2009; Manolio et al. 2009) and “genetic dark matter” (Galvan et al. 2010; Martin and Chang 2012; Melhem and Devlin 2010). Even assuming that a host of rare alleles account for some unexplained phenotypic variation (Kraft and Hunter 2009), it is reasonable to posit that E and G × E are the primary causes of chronic diseases, as suggested by studies of families and twins (Hemminki et al. 2006; Lichtenstein et al. 2000), epigenetics (Gluckman et al. 2008, 2010; Smith and Meissner 2013), and gene-expression profiles that change with lifestyles and infections (Chen et al. 2012; Preininger et al. 2013). In fact, as shown in Figure 1, about half of the 50 million global deaths in 2010 were attributed to a small set of exposures, dominated by particulate air pollution (combined effects of ambient particles and household smoke), smoking (active and passive), and diet (Lim et al. 2012). This conundrum—where scientists use high-tech omics to detect small effects of G but rely upon low-tech methods to study potentially large effects of E and G × E—has produced a very uneven record of etiologic research.

One way to level the playing field would be to explore health impacts of E and G × E with exposome-wide association studies (EWAS) (Rappaport 2012) that obtain comprehensive, quantitative measurements of chemicals in human biospecimens (Holmes et al. 2008; Ritchie et al. 2010; Wang Z et al. 2011). This approach recognizes that meaningful exposures are mediated in the internal chemical environment (Rappaport and Smith 2010) by endogenous signaling molecules, exogenous chemicals, and reactive electrophiles (E-factors) that communicate with cells, tissues, and organs via mutations, posttranslational modifications, enzymes, transcription factors, and receptors (G-factors) (Brodsky and Medzhitov 2009; Liebler 2008; Menon and Manning 2013). Because blood transports chemicals to and from tissues and represents a reservoir of all endogenous and exogenous chemicals in the body at a given time (Nicholson et al. 2012b), the blood exposome offers a parsimonious but essentially unexplored means for interrogating biologically relevant exposures (Rappaport 2012).

## Methods

*Sources of data*. To investigate the portion of the blood exposome represented by small molecules and metals, we obtained blood concentrations of 1,561 chemicals from samples of healthy human populations compiled by the Human Metabolome Database (HMDB; http://www.hmdb.ca) (Wishart et al. 2013) (1,451 chemicals) and the U.S. National Health and Nutrition Examination Survey (NHANES) [Centers for Disease Control and Prevention (CDC) 2009, 2012, 2013] (110 chemicals). Each molecule or metal was assigned one of the following four source categories: *a*) endogenous chemical (from intrinsic human metabolism; *n* = 1,223), *b*) food chemical (*n* = 195), *c*) pollutant (*n* = 94), or *d*) drug (*n* = 49). (The process for selecting chemicals is described in Supplemental Material, pp. 2–4.) To link individual chemicals with chronic-disease risks and systems biology, we retrieved additional data from the National Center for Biotechnology Information databases PubMed (http://www.ncbi.nlm.nih.gov/pubmed; citations on chronic-disease risk factors) and Biosystems (http://www.ncbi.nlm.nih.gov/biosystems/; data on human metabolic pathways). Although modest in size, these samples allowed us to explore the range of human blood concentrations, to test for differences in median levels across source categories and to map chemical similarities after weighting by blood concentration, disease-risk citations, and human metabolic pathways. Relevant data are given in Supplemental Material, Table S1.

HMDB entries were from metabolic studies in mostly Western populations, and included endogenous and food chemicals, drugs, and pollutants; NHANES included only nutrients and pollutants in U.S. populations. When a given chemical was present in both of these databases, we used NHANES concentrations. If the same chemical had been reported in more than one study or year, we used the geometric mean concentration. Numbers of individual subjects varied across chemicals. Drug concentrations were reported in clinical trials at therapeutic doses.

We used Chemical Abstract Service (CAS) registry number(s) as the query parameter to search PubMed along with medical subject headings (MeSH) annotations to retrieve the citations describing epidemiological studies. The search string was

(blood OR plasma OR serum) AND (“risk factors”[MeSH Terms] OR “relative risk*” OR “odds ratio*” OR “hazard ratio*”) + CAS number + [EC/RN number](“journal article”[pt] NOT review[pt] NOT “meta analysis”[pt]) (hasabstract[text] AND “humans”[MeSH Terms]) english[lang] (neoplasms[mesh] OR diabetes[mesh] OR “cardiovascular diseases”[mesh] OR “Respiratory Tract Diseases”[mesh]).

For retrieval of pathway hits, PubChem identifiers for each compound were searched against the Biosystems database. Chemical similarity maps were generated using MetaMapp (http://metamapp.fiehnlab.ucdavis.edu/homePage).

*Statistical analysis*. Differences in median blood concentrations across source categories were evaluated with Kruskal-Wallis tests via SAS for Windows (v.9.3) (SAS Institute Inc., Cary, NC).

## Results

**Figure 2:**
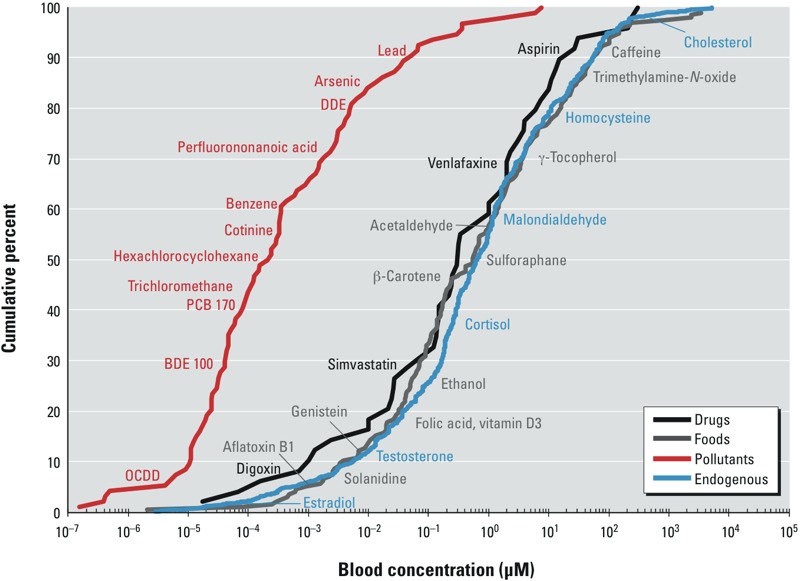
Small molecules and metals in human blood. Each curve represents the cumulative distribution of chemical concentrations from a particular source category (pollutants, *n* = 94; drugs, *n* = 49; food chemicals, *n* = 195; endogenous chemicals, *n* = 1,223). Abbreviations: BDE 100, 2,2′,4,4′,6-pentabromo­diphenyl ether; DDE, 1,1-bis-(4-chlorophenyl)-2,2-dichloroethene; OCDD, 1,2,3,4,6,7,8,9-octachloro­oxanthrene; PCB 170, 2,2′,3,3′,4,4′,5-heptachloro-1,1′-biphenyl.

*Blood concentrations*. Cumulative distributions of blood concentrations are shown in Figure 2 for the four sources of chemicals. Concentrations ranged from 160 fM to 140 mM, a staggering 11 orders of magnitude. Within each category, concentrations covered a 10^7^-fold range. Median blood levels of endogenous chemicals (0.94 μM), food chemicals (1.00 μM), and drugs (0.30 μM) were not significantly different (*p* = 0.246). In contrast, pollutant concentrations were 1,000 times lower (median, 2.4 × 10^–4^ μM, *p* < 0.0001), and only pollutants with blood levels above the median value overlapped with other distributions.

**Figure 3:**
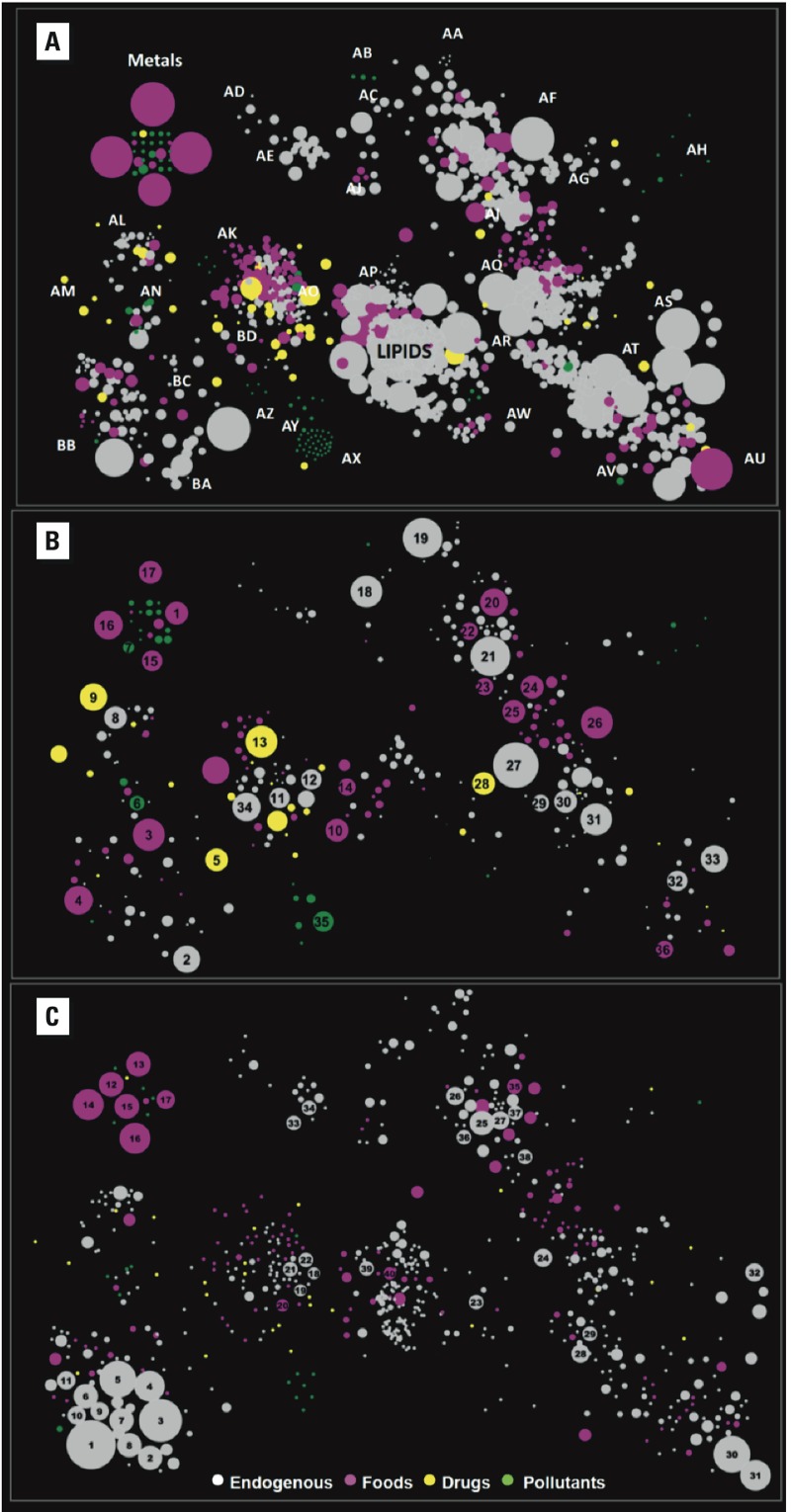
Chemical-similarity maps of small molecules and metals in human blood (Tanimoto coefficient ≥ 0.7; symbol color represents the source category). (*A*) All chemicals (*n* = 1,561; symbol size reflects the blood/serum concentration). Map locations: AA, leucotrienes; AB, perfluorinated compounds; AC, alkylamines; AD, pteridines; AE, pyrimidine nucleotides; AF, aliphatic amino acids and derivatives; AG, sphingo­lipids; AH, organochlorine pesticides; AI, prenol lipids; AJ, sulfur compounds; AK, flavonoids; AL, pyrroles and indoles; AM, pyridines; AN, alkaloids; AO, benzoic acids and phenols; AP, eicosanoids; AQ, fatty acids and fatty amines; AR, steroids; AS, organic acids; AT, monosaccharides; AU, phosphates; AV, alcohols; AW, fatty acid esters and conjugates; AX, polychlorinated biphenyls; AY, simple aromatics; AZ, chlorinated dioxins and furans; BA, sulfates and nitrites/nitrates; BB, purine nucleotides; BC, aromatic amino acids and derivatives; BD, benzoic acids and phenols. (*B*) Matching chemicals from (*A*) cited in studies of chronic-disease risks (*n* = 336; symbol size reflects the number of citations). Map locations: 1, selenium; 2, nitric oxide; 3, folic acid; 4, vitamin B12; 5, metformin; 6, cotinine; 7, lead; 8, bilirubin; 9, atorvastatin; 10, ascorbic acid; 11, thyroxine; 12, nor­epinephrine; 13, aspirin; 14, eicosapentaenoic acid; 15, magnesium; 16, calcium; 17, sodium; 18, uric acid; 19, creatinine; 20, l-arginine; 21, homocysteine; 22, l-methionine; 23, l-valine; 24, β-carotene; 25, vitamin A; 26, vitamin D3; 27, cholesterol; 28, simvastatin; 29, aldosterone; 30, cortisol; 31, testosterone; 32, malon­dialdehyde; 33, ᴅ-glucose; 34, estradiol; 35, PCBs; 36, ethanol. (*C*) Matching chemicals from (*A*) having human metabolic pathways (*n* = 658; symbol size reflects the number of pathways). Map locations: 1, adenosine triphosphate; 2, hydrogen peroxide; 3, adeno­sine diphosphate; 4, guano­sine diphosphate; 5, guanosine triphosphate; 6, NADPH; 7, cyclic AMP; 8, adenosine monophosphate; 9, NADH; 10, NAD; 11, FAD; 12, manganese; 13, sodium; 14, calcium; 15, zinc; 16, magnesium; 17, potassium; 18, norepinephrine; 19, epinephrine; 20, ʟ‑phenyl­alanine; 21, ʟ‑tyrosine; 22, dopamine; 23, palmitic acid; 24, cholesterol; 25, ʟ‑glutamic acid; 26, adenine; 27, ʟ‑aspartic acid; 28, oxoglutaric acid; 29, pyruvic acid; 30, phosphate; 31, pyrophosphate; 32, formic acid; 33, uridine 5′-mono­phosphate; 34, uridine 5′-diphosphate; 35, ʟ‑arginine; 36, ʟ‑alanine; 37, ʟ‑cysteine; 38, ʟ‑serine; 39, arachodonic acid; 40, α-linolenic acid.

*Chemical-similarity maps*. Endogenous and dietary molecules comprised > 100 chemical classes, particularly lipids, steroids, amino acids, fatty acids, and nucleotides (see Supplemental Material, Table S1). In addition to nutrients and vitamins, food chemicals included such bioactive molecules as aflatoxin B1 (a carcinogen from mold-infected grains and nuts), solanidine (a toxin from potatoes), sulforaphane (a DNA-protective agent from cruciferous vegetables), acetaldehyde (a mutagen from metabolism of alcohol), genistein (an endocrine-disrupting chemical from soy products) and trimethylamine-*N*-oxide (from metabolism of choline and carnitine; a suspected cause of atherosclerosis). Exogenous pollutants were primarily halogenated compounds—trihalomethanes, chlorinated pesticides, perfluorinated compounds, polychlorinated biphenyls (PCBs), brominated diphenyl ethers, and some chlorinated dioxins and furans—and metals, but also included a few volatile aromatic species (notably benzene) and metabolites of nicotine. This diversity is illustrated in Figure 3A, which maps the 1,561 chemicals by their structural similarities (Barupal et al. 2012), with symbol sizes indicating blood concentrations. Shown in Figure 3A, constellations of biochemical classes were populated largely by endogenous and food chemicals, whereas drugs clustered with aromatic compounds [between map locations AN (alkaloids) and BD (benzoic acids and phenols)] and pollutants were mainly at map peripheries [e.g., locations AH (organochlorine pesticides) and AX (PCBs)]. Metals and metalloids originated from foods (six most abundant: sodium, potassium, iron, calcium, phosphorus, and magnesium), pollution (six most abundant: silicon, strontium, nickel, lead, beryllium, and arsenic), and one drug (lithium).

Because citations to risk factors summarize epidemiological and clinical evidence associating a chemical with disease phenotypes, we found PubMed citations for 960 searchable substances in our inventory (only chemicals with CAS registry numbers were searchable in PubMed), and obtained 19,656 citations matching 336 (35%) of these chemicals. (Numbers of matching citations are included in Supplemental Material, Table S1.) The distribution of citations per chemical was highly skewed, with a median value of 7.5 and a maximum of 4,499 (cholesterol). The large numbers of citations per chemical and positive skewness probably reflect publication bias in hypothesis-driven epidemiological studies and clinical trials. Median numbers of citations varied 2-fold across source categories (drugs, 10; endogenous, 6; food chemicals, 13; pollutants, 6; *p* = 0.041). When food chemicals were removed, median values for the other categories were not significantly different (*p* = 0.307). This indicates that a typical food chemical was about twice as likely to be cited as a chronic-disease-risk factor than a chemical from another category.

The chemical-similarity map for these 336 chemicals is shown in Figure 3B, where symbol size reflects the number of citations. This map shared prominent clustering patterns with Figure 3A, except that individual lipid molecules were largely absent (lipids tend to be reported as classes rather than discrete molecules in clinical and epidemiological studies) and most endogenous molecules with large blood concentrations had few PubMed citations. Several highly cited chemicals are familiar biomarkers of human diseases and causal exposures: for example, cholesterol (*n* = 4,449, cardiovascular disease), folic acid (*n* = 595, cancer and neural-tube defects), lead (*n* = 65, cardiovascular and neurological diseases), and cotinine (*n* = 78, smoking-related diseases), along with vitamins, hormones, and antioxidants. Aspirin was the most-cited drug (*n* = 515), followed by atorvastatin (*n* = 206).

Sequencing the human genome motivated mapping of G-centric molecular pathways at multiple levels and made metabolites with annotated pathways desirable targets for systems biology (Chen et al. 2012). In matching records retrieved from the Biosystems database for chemicals in our inventory, at least one human metabolic pathway had been reported for 658 of them (42%). (The numbers of pathways reported are included in Supplemental Material, Table S1.) Median numbers of pathways varied 6-fold across sources, with pollutants being significantly understudied (drugs, 4; endogenous, 6; food chemicals, 4; pollutants, 1; *p* < 0.0001). The chemical-similarity map of these 658 chemicals is shown in Figure 3C, with symbol size representing the number of pathways. The largest numbers of pathways corresponded to purine-nucleotide phosphates (maximum of 707 for adenosine triphosphate), amino acids and derivatives, fatty acids, and dietary metals. In contrast to prominent disease-risk citations that were distributed more or less evenly across source categories (Figure 3B), chemicals with many pathways were overwhelmingly endogenous molecules and essential nutrients (Figure 3C).

Because the sets of PubMed and Biosystems hits were not completely overlapping, we repeated the analysis of source categories for the 267 chemicals that had at least one disease-risk citation and at least one human metabolic pathway. Results from this subset of chemicals were essentially the same as for the complete data sets. Median numbers of PubMed hits varied 2.4-fold across source categories (drugs, 7; endogenous, 7; food chemicals, 17; pollutants, 9; *p* = 0.0261) but did not differ significantly when food chemicals were removed (*p* = 0.4135). In contrast, median numbers of human metabolic pathways varied 12-fold across source categories, and were much smaller for drugs and pollutants than for endogenous and food chemicals (drugs, 4; endogenous, 11.5; food chemicals, 12; pollutants, 1; *p* < 0.0001).

## Discussion

*Discovering causes of disease*. Data summarized in Figure 1 suggest that only about half of the current burden of chronic diseases can be attributed to known exposures and thus motivate more thorough scrutiny of the exposome to find unknown causes. This will be challenging because of the remarkable ranges of human exposures across sources and chemical classes that are displayed in Figures 2 and 3. Such extreme variation suggests that knowledge-driven studies are ill suited for discovering unknown causes of chronic diseases. There are simply too many diverse chemicals covering too great a concentration range to formulate reasonable hypotheses. We should narrow the list of chemical candidates by using EWAS to find discriminating exposures in biospecimens from diseased and healthy subjects (Holmes et al. 2008; Patel et al. 2010; Rappaport 2012; Ritchie et al. 2010; Wang Z et al. 2011), essentially following the same strategy as GWAS. Once identified, these chemicals can be targeted to investigate sources, causality, disease mechanisms, and interventions (Rappaport 2012). A good example of this two-stage strategy was provided by Hazen and coworkers, who linked risks of cardiovascular disease with blood concentrations of trimethylamine-*N*-oxide, a metabolite of choline and carnitine derived from microbial/human metabolism (Koeth et al. 2013; Tang et al. 2013; Wang Z et al. 2011).

Optimally, EWAS would employ untargeted methods to compare blood exposomes between cases and controls nested in cohort studies. Although untargeted high-resolution mass spectrometry (MS) can detect > 30,000 features of small molecules in human serum (Ivanisevic et al. 2013), use of untargeted platforms in our laboratories cannot reliably measure blood concentrations less than approximately 0.1 μM in 50 μL of serum. Given the extraordinary dynamic range of small molecules and metals (Figure 2), untargeted analyses may miss about 90% of pollutants and 30% of endogenous and food chemicals, including hormones (e.g., estradiol, testosterone), carcinogens (e.g., aflatoxin B1, benzene), and endocrine disruptors [e.g., genistein, PCBs, DDE (1,1-bis-(4-chlorophenyl)-2,2-dichloroethene)]. Thus, although increased sensitivity can be anticipated with untargeted MS, EWAS currently require a combination of untargeted (Holmes et al. 2008; Ritchie et al. 2010; Wang Z et al. 2011) and semitargeted (Patel et al. 2010) methods to quantify exposures. In addition, as for the Human Genome Project (National Human Genome Research Institute 2013), different laboratories could address specific parts of the exposome in a complementary and collaborative way.

*Magnitudes of exposures*. Ranges of blood concentrations varied greatly within and between sources of exposure as shown in Figure 2. Although we had anticipated that endogenous and food chemicals would have similar blood levels, we were surprised to observe the near-perfect overlap of concentrations of these chemicals with those of drugs. Such similar cumulative distributions suggest that blood concentrations of endogenous human metabolites and food chemicals are in the therapeutic range of pharmacologic agents. We were also somewhat surprised to observe that blood concentrations of pollutants were 1,000 times lower than those of chemicals from other categories. Such disparate blood levels across exposure sources awaken arguments by Ames and colleagues that natural toxins and protective chemicals are consumed in much greater quantities than synthetic chemicals and, therefore, should be considered when assessing disease risks (Ames 1983; Ames et al. 1987, 1990a, 1990b). This further emphasizes the importance of EWAS for interrogating all chemicals that can cause chronic diseases.

*Epidemiology and systems biology*. Weighting chemicals by blood concentrations (Figure 3A), epidemiological (risk factor) citations (Figure 3B), or human metabolic pathways (Figure 3C) altered the appearances of chemical-similarity maps. Epidemiological citations downgraded the importance of endogenous molecules while upgrading pollutants and drugs, but weighting by numbers of metabolic pathways had the opposite effect. These markedly different maps were unanticipated because it is generally thought that epidemiology and systems biology work hand in glove to elucidate causes and mechanisms of disease (Nicholson et al. 2012b).

Epidemiologists are interested in causes of disease, including genetic factors (G) and exposures (E) related to metabolism, diet, pollution, infections, lifestyles, and behaviors. When they have used blood concentrations to quantify chemical exposures from G, E, and G × E, epidemiologists have successfully linked chronic diseases to targeted endogenous and exogenous chemicals (Figures 1 and 3B). We assumed that chemicals that had been repeatedly associated with chronic diseases (Figure 3B) would be logical candidates for exploration of metabolic pathways. However, because only 29% of the chemicals in our database with three or more PubMed risk-factor citations also had a Biosystems hit (i.e., 189/658), this was apparently not the case.

Systems biologists focus on metabolic pathways that are under homeostatic control and, therefore, presume a G-centric hierarchy that culminates in the endogenous metabolome (Nicholson et al. 2012b). From the systems-biology perspective, the most meaningful metabolites are those that participate in many pathways (Loscalzo et al. 2007), and Figure 3C points to products of energy metabolism and essential nutrients as filling that role. If such molecules can be linked to disease, then their concentrations can promote early diagnosis and treatment even if causal E and G × E factors are unknown. For example, high concentrations of branched-chain amino acids (leucine, isoleucine, and valine) predict incipient diabetes and offer avenues for treatment (Newgard 2012; Wang TJ et al. 2011). However, the poor track record of GWAS in explaining the variation of chronic diseases suggests that systems biologists who look only at endogenous metabolites (i.e., molecules produced under human genomic control) will miss opportunities to discover causal pathways. Indeed, of the 41,000 small molecules currently thought to populate the human body (Wishart et al. 2013), only 2,626 (6.4%) (Recon X 2013) are products of endogenous human metabolism.

*The microbiome*. When considering G and G × E effects, it is important to remember that 90% of the approximately 10^14^ cells in the human body actually reside in the gut microbiota (Savage 1977). This superorganism contributes ~ 500,000 microbial protein-coding genes (Qin et al. 2010) compared with a human complement of ~ 20,000 protein-coding genes. Thus, human biospecimens contain a plethora of bioactive molecules generated from microbial metabolism (Nicholson et al. 2012a) in addition to chemicals introduced by the diet, drugs, infectious organisms, pollution, and lifestyle factors (Nicholson and Wilson 2003; Rappaport and Smith 2010). Chemicals produced by the microbiota control development and maintenance of the human immune system as well as important cell-signaling processes (Nicholson et al. 2012a) and appear to be intimately involved in development of chronic diseases (Blumberg and Powrie 2012; Haiser and Turnbaugh 2012). Although research involving microbial contributions to the human exposome is in its infancy, it should expand dramatically as the important roles played by the microbiota are recognized in disease etiology (Koeth et al. 2013; Ridaura et al. 2013; Tang et al. 2013; Wang Z et al. 2011).

*Internal and external measures of exposure*. To discover unknown exposures that cause disease, we advocate data-driven EWAS that profile chemicals in blood from disease cases and controls (Rappaport 2012). Internal measures of exposure, such as the blood exposome, offer advantages for EWAS because they represent all sources of chemicals, including those generated inside the body, and blood specimens are often archived in prospective cohort studies (Rappaport and Smith 2010). As EWAS discover new disease associations, knowledge-driven studies will be needed to curate exposure sources and quantify exposure–response relationships—thereby strengthening causal inferences—and to suggest interventions (Rappaport 2012). To the extent that important exposures originate outside the body, this follow-up will involve exposure scientists, industrial hygienists, food scientists, and analytical chemists who measure chemicals in air, water, and food, as well as biologists who evaluate mechanisms of action (Lioy and Rappaport 2011; Rappaport 2011; Scalbert et al. 2014; Wild 2012). Thus, the process of identifying causal exposures can require measurements of chemicals both inside and outside the body across a diverse scientific milieu.

*Limitations*. Because we relied on publically accessible data, our findings and their interpretation are conditioned by the chemicals compiled by the HMDB and NHANES and by publications and metabolic pathways curated through the National Center for Biotechnology Information. Most of the 1,561 chemicals we investigated in human blood were derived from foods and endogenous processes because these are major foci of the HMDB. Most of the pollutants in our database were reported by NHANES. Yet, we exclued a roughly equal number of other pollutants in NHANES because they were not detected in most blood samples (CDC 2009, 2012, 2013). If nondetects from NHANES had been included, the shift toward lower blood concentrations of pollutants relative to chemicals from other sources would have been even greater. We also recognize that some of our data could be biased. For example, using PubMed citations to assess disease associations of particular exposures can introduce biases related to prior publications and to research priorities for different diseases, numbers of investigators, journals, and so on. As noted previously, the Biosystems database of human metabolic pathways reflects apparent biases favoring chemicals that are involved in many pathways regardless of disease associations. Finally, we were unable to investigate possible effects of chemical interactions on disease risks. Despite these limitations, the vast diversity and concentration ranges of blood chemicals should be apparent, as should differences in median blood concentrations observed across source categories (Figures 2 and 3).

## Conclusions

The extreme complexity and dynamic range of the blood exposome (Figures 2 and 3) should motivate data-driven studies to discover unknown causes of chronic diseases, regardless of their exogenous and endogenous origins (Rappaport 2012). Candidate exposures can be identified by EWAS that compare omic profiles in blood from diseased and healthy subjects.

The apparent disconnect between chemical-specific disease risks (Figure 3B) and human metabolic pathways (Figure 3C) indicates that systems biologists are only marginally engaged in elucidating causal disease pathways. We promote a more global approach to systems biology (Nicholson and Wilson 2003) that expands beyond the endogenous metabolome to the blood exposome, illustrated here by a large sample of circulating small molecules and inorganic species.

Perhaps the most compelling reason for embracing the blood exposome is the potential to discover all chemicals that cause disease and then to intervene in order to modify exposures and the concomitant burden of disease (Christiani 2011). The current inventory of small molecules and metals associated with chronic diseases consists of about 300 chemicals that have been targeted repeatedly in epidemiological and clinical studies (Figure 3B). With recognition of their health significance, these chemicals have been routinely monitored for clinical interventions (e.g., cholesterol, folic acid, vitamins) and as regulated pollutants (e.g., lead, arsenic, benzene, PCBs). Yet, further scrutiny of these recognized health hazards adds little to our understanding of disease causation. If we expect to reduce the burden of chronic diseases, it is time to find the undiscovered health-impairing and health-promoting chemicals to which humans are exposed (Figure 1), not only small molecules and metals but also proteins and foreign DNA and RNA.

## Supplemental Material

(5.5 MB) PDF
