# Supplementary material for: The Blood Exposome and Its Role in Discovering Causes of Disease
Source: Environ Health Perspect. 2014 Mar 21;122(8):769–74. doi: 10.1289/ehp.1308015 (PMC4123034; doi:10.1289/ehp.1308015)
Supplement: (5.5 MB) PDF [file ehp.1308015.s001.pdf]

Environmental Health Perspectives doi:10.1289/ehp.1308015

SUPPLEMENTAL MATERIAL

The Blood Exposome and Its Role in Discovering Causes of Disease

Stephen M. Rappaport, Dinesh K. Barupal, David Wishart, Paolo Vineis, and Augustin Scalbert

Table S1. Database of 1,561 chemicals measured in blood from normal human populations. Concentrations of chemicals were compiled from the Human Metabolome Database (HMDB) and the U.S. National Health and Nutrition Examination Survey (NHANES) as described below. If multiple entries were obtained for the same chemical, chemical-specific geometric mean values were estimated.

HMDB chemicals, accessed on 4/15/13 at http://www.hmdb.ca, included 3,426 entries designated as 'Normal Blood Concentration Entries' from 'Adults' of either or both sexes (there were multiple entries for some chemicals). Each entry was a mean blood concentration (micromolar) over all subjects in the reported sample. Some chemicals with extremely high blood concentrations were removed (water, oxygen, carbon dioxide, bromide, iodide, chloride, sulfide and Fe2+) as were chemicals with concentrations either missing or reported as 'less than' some value. Upon examination of data and references, some additional entries were removed [acetone, alpha-cholesterol, formaldehyde, one unrealistic entry for methanol (468 mM) and several series of structurally similar glycerolipids, glycerophospholipids and sphingolipids with identical concentration values. Each entry was examined independently by two of the authors to determine the most relevant source category, which sometimes differed from the 'Origin' field in HMDB. If a HMDB chemical was the same as listed in the NHANES database, it was deleted. This left a total of 2,519 entries for 1,451 chemicals.

NHANES chemicals included 135 entries for 'Environmental Chemicals' (designated as 'pollutants' in our analyses) and 44 entries for 'Nutrients' (designated as 'food chemicals') yielding a total of 179 entries (CDC 2009, 2012, 2013). Each entry was either a geometric mean (if provided) or median concentration measured in serum or blood from all subjects (combined for gender, race and age, designated by NHANES as 'Total') during one year, except for 1,4-

dichlorobenzene that was reported for Mexican Americans only. (Chemicals without reported geometric mean or median values were excluded). Concentrations of a given chemical that were measured in different years were listed as separate entries. All chemicals were measured in adult populations except the following nutrients that included infants and/or children as well as adults: folate, pyridoxal-5'-phosphate, 4-pyridoxic acid, vitamin B12, methylmalonic acid, vitamin C, vitamin A, retinyl palmitate, vitamin E, gamma-tocopherol, alpha-carotene, trans-beta-carotene, beta-cryptoxanthin, lutein/zeaxanthin, trans-lycopene, lycopene (total) and 25-hydroxyvitamin D. The following fatty acids were renamed: docosanoic acid (22:0) to behenic acid, lignoceric acid (24:0) to tetracosanoic acid, docosenoic acid (22:1n-9) to erucic acid, homo-gamma-linolenic acid (20:3n-6) to 8,11,14 eicosatrienoic acid, and docosatetranoic acid (22:4n-6) to adrenic acid. The final dataset included 179 entries for 110 chemicals. Blood concentrations were converted to micromolar values assuming 1.025 g serum/ml and 0.0065 g lipid/g serum, as needed.

Variables:

PC_CID, PubChem ID

Chemical, chemical name

Dataset, (HMDB or NHANES)

Source, exposure source (Endo=endogenous, Drug=drug, Food=food, Pollut=pollutant)

Concentration (μM), geometric mean blood (or serum/plasma) concentration, μM, across studies

No. studies, number of studies (or NHANES years) that contributed data for estimating the blood concentration

Class, chemical class

PubMed, number of NCBI PubMed citations to risk factors for chronic diseases (respiratory, cardiovascular, cancer and diabetes)

Biosys, number of NCBI Biosys human metabolic pathways

CAS, chemical abstract service registry number

References

CDC. 2009. Fourth national report on human exposure to environmental chemicals. Atlanta, GA:National Center for Environmental Health, Centers for Disease Control and Prevention.

CDC. 2012. Second national report on biochemical indicators of diet and nutrition in the u.S. Population. Atlanta, Georgia:National Center for Environmental Health, Centers for Disease Control.

CDC. 2013. Fourth national report on human exposure to environmental chemicals, updated tables. Atlanta, GA:National Center for Environmental Health, Centers for Disease Control and Prevention.

Table S1. Database of 1,561 chemicals measured in blood from normal human populations.

PC_CID	Chemical	Dataset	Source	Concentration (µM)	No. studies	Class	PubMed	Biosys	CAS
92135	(R)-3-Hydroxybutyric acid	HMDB	Endo	48.9990054	3	Hydroxy Acids and Derivatives	0	10	625-72-9
11217234	(R)-3-Hydroxyisobutyric acid	HMDB	Endo	20.99952881	1	Hydroxy Acids and Derivatives	0	0	1910-47-0
54456	(R)-Salsolinol	HMDB	Endo	0.000961227	2	Isoquinolines	0	0	525-72-4
150929	(S)-3,4-Dihydroxybutyric acid	HMDB	Endo	18.00050837	1	Hydroxy Acids and Derivatives	0	0	51267-44-8
94318	(S)-3-Hydroxybutyric acid	HMDB	Endo	19.99935454	1	Hydroxy Acids and Derivatives	0	0	6168-83-8
440873	(S)-3-Hydroxyisobutyric acid	HMDB	Endo	20.99952881	1	Hydroxy Acids and Derivatives	0	2	2068-83-9
439434	(S)-b-aminoisobutyric acid	HMDB	Endo	1.030042435	1	Amino Acids and Derivatives	0	1	4249-19-8
163959	1-(alpha-Methyl-4-(2-methylpropyl)benzeneacetate)-beta-D-Glucopyranuronic acid	HMDB	Drug	12.99935837	1	Prenol Lipids	0	0	115075-59-7
4091	1,1-Dimethylbiguanide	HMDB	Drug	6.00024319	1	Guanidines	163	6	657-24-9
428	1,3-Diaminopropane	HMDB	Endo	0.039999033	1	Alkylamines	0	7	109-76-2
4685	1,4-Dichlorobenzene	NHANES	Pollut	0.002754553	3	Aromatic Homomonocyclic Compounds	0	1	106-46-7
64960	1,5-Anhydrosorbitol	HMDB	Endo	149.994706	1	Monosaccharides	5	11	154-58-5
5282259	10-Nitrolinoleic acid	HMDB	Endo	0.079000587	1	Lineolic Acids and Derivatives	0	0	774603-04-2
5283168	11(R)-HETE	HMDB	Endo	0.018854569	4	Eicosanoids	0	1	73347-43-0
5283146	11,12-DiHETrE	HMDB	Endo	0.022308221	4	Eicosanoids	0	8	192461-95-3
53480479	11,12-EpETrE	HMDB	Endo	0.045411041	3	Eicosanoids	0	0	.
101788	11b-Hydroxyprogesterone	HMDB	Endo	1.99996E-05	1	Steroids and Steroid Derivatives	0	1	600-57-7
5280886	11b-PGF2a	HMDB	Endo	0.000178147	2	Eicosanoids	0	5	.
5280891	11-Dehydro-thromboxane B2	HMDB	Endo	0.0027496	2	Eicosanoids	13	7	67910-12-7
11954058	11H-14,15-EETA	HMDB	Endo	0.884528981	2	Fatty Acids and Conjugates	0	2	219535-29-2
5283134	11-trans-Leukotriene C4	HMDB	Endo	8.99987E-06	1	Peptides	0	0	74841-69-3
53481695	11Z-Octadecenylcarnitine	HMDB	Endo	0.10999724	1	Fatty Acid Esters	0	0	.
5283007	12(13)Ep-9-KODE	HMDB	Endo	3.458379062	2	Fatty Acids and Conjugates	0	0	.
5280892	12(S)-HPETE	HMDB	Endo	1.450052844	1	Eicosanoids	0	7	71774-10-2
10236635	12,13-DHOME	HMDB	Endo	0.247115362	4	Fatty Acids and Conjugates	0	1	.
16061067	12,13-DiHODE	HMDB	Endo	0.218996397	1	Lineolic Acids and Derivatives	0	0	.
5356421	12,13-EpOME	HMDB	Endo	0.63419424	3	Fatty Acids and Conjugates	0	0	.

PC_CID	Chemical	Dataset	Source	Concentration (µM)	No. studies	Class	PubMed	Biosys	CAS
10041593	12-HEPE	HMDB	Endo	0.035465319	4	Eicosanoids	0	0	74838-73-6,109430-12-8,116180-17-7,81187-21-5
5283155	12-HETE	HMDB	Endo	0.287681878	5	Eicosanoids	0	11	54397-83-0
5283141	12S-HHT	HMDB	Endo	0.00203004	1	Fatty Alcohols	0	4	54397-84-1
161273	13,14-Dihydro PGE1	HMDB	Endo	2.79998E-06	1	Eicosanoids	0	0	19313-28-1
5283039	13,14-Dihydro-15-keto PGF2a	HMDB	Endo	0.000193913	2	Eicosanoids	1	4	27376-76-7
5280711	13,14-Dihydro-15-keto-PGE2	HMDB	Endo	0.000340839	4	Eicosanoids	0	4	363-23-5
5282379	13-cis-Retinoic acid	HMDB	Endo	0.003000129	1	Prenol Lipids	6	0	4759-48-2,97950-17-9
10469728	13-HOTE	HMDB	Endo	0.158833309	3	Lineolic Acids and Derivatives	0	0	.
5280720	13-L-Hydroperoxylinoleic acid	HMDB	Endo	6.009851263	1	Lineolic Acids and Derivatives	0	2	33964-75-9
6446027	13-OxoODE	HMDB	Endo	0.066324229	4	Lineolic Acids and Derivatives	0	1	54739-30-9
6443013	13S-hydroxyoctadecadienoic acid	HMDB	Endo	1.609623016	4	Lineolic Acids and Derivatives	0	9	5204-88-6
16061119	14,15-DiHETE	HMDB	Endo	0.024409082	2	Eicosanoids	0	0	.
5283147	14,15-DiHETrE	HMDB	Endo	0.029537342	4	Eicosanoids	0	8	77667-09-5
16061088	14,15-EpETE	HMDB	Endo	0.005366389	2	Fatty Acids and Conjugates	0	0	.
5353279	14,15-Epoxy-5,8,11-eicosatrienoic acid	HMDB	Endo	0.000484254	2	Fatty Acids and Conjugates	0	13	81276-03-1
5283201	14R,15S-EpETrE	HMDB	Endo	0.000133996	1	Eicosanoids	0	0	98103-48-1
16061062	15(16)-EpODE	HMDB	Endo	0.309406294	3	Fatty Acids and Conjugates	0	0	.
5280724	15(S)-HETE	HMDB	Endo	0.073138527	7	Eicosanoids	0	6	54845-95-3
5280893	15(S)-HPETE	HMDB	Endo	1.060032958	1	Eicosanoids	0	9	70981-96-3
5283145	15(S)-Hydroxyeicosatrienoic acid	HMDB	Endo	0.051257158	3	Eicosanoids	0	0	13-16-1
16061068	15,16-DiHODE	HMDB	Endo	1.076268537	3	Lineolic Acids and Derivatives	0	0	.
5311211	15-Deoxy-d-12,14-PGJ2	HMDB	Endo	0.001521437	3	Eicosanoids	0	1	87893-55-8
53480357	15-HEPE	HMDB	Endo	0.012674033	4	Eicosanoids	0	0	97850-14-1
5280701	15-KETE	HMDB	Endo	0.012746482	4	Fatty Acids and Conjugates	0	5	81416-72-0
5283042	15-Keto-13,14-dihydroprostaglandin A2	HMDB	Endo	0.000129997	1	Eicosanoids	0	0	74872-89-2
5280719	15-Keto-prostaglandin E2	HMDB	Endo	0.000599989	1	Eicosanoids	0	5	26441-05-4
5280887	15-Keto-prostaglandin F2a	HMDB	Endo	0.000198227	2	Eicosanoids	0	5	35850-13-6
14392758	16(17)-EpDPE	HMDB	Endo	0.009377895	2	Fatty Acids and Conjugates	0	0	.
9548884	16(R)-HETE	HMDB	Endo	0.000276998	1	Eicosanoids	0	1	.
115116	16a-Hydroxyestrone	HMDB	Endo	0.000780013	1	Steroids and Steroid Derivatives	3	0	566-76-7
16061120	17,18-DiHETE	HMDB	Endo	0.793818844	3	Eicosanoids	0	0	.

PC_CID	Chemical	Dataset	Source	Concentration (µM)	No. studies	Class	PubMed	Biosys	CAS
16061089	17,18-EpETE	HMDB	Endo	0.002852384	2	Fatty Acids and Conjugates	0	0	131339-23-6
68570	17a-Estradiol	HMDB	Endo	0.320010971	1	Steroids and Steroid Derivatives	0	3	57-91-0
5991	17a-Ethynylestradiol	HMDB	Drug	0.000160006	1	Steroids and Steroid Derivatives	38	0	57-63-6,406932-93-2,77538-56-8
91451	17a-Hydroxypregnenolone	HMDB	Endo	0.004242511	2	Steroids and Steroid Derivatives	0	9	387-79-1
13783824	17-beta-estradiol-3-glucuronide	HMDB	Endo	3.18587E-05	3	Steroids and Steroid Derivatives	0	0	.
6439179	17-HDoHE	HMDB	Endo	0.01224546	2	Fatty Acids and Conjugates	0	0	90780-52-2
6442740	17-HETE	HMDB	Endo	0.000113003	1	Eicosanoids	0	4	128914-47-6,183509-25-3
6442778	18-Hydroxyarachidonic acid	HMDB	Endo	0.000273011	1	Eicosanoids	0	4	128656-74-6
123655	18-Hydroxycortisol	HMDB	Endo	0.003289894	1	Steroids and Steroid Derivatives	0	0	864-27-7
123712	18-Oxocortisol	HMDB	Endo	0.000827005	1	Steroids and Steroid Derivatives	0	0	2410-60-8
53481496	18R-HEPE	HMDB	Endo	0.000137994	1	Eicosanoids	0	0	.
16061148	19,20-DiHDPA	HMDB	Endo	0.018734285	3	Fatty Acids and Conjugates	0	0	.
128251	19-Norandrosterone	HMDB	Endo	0.560010357	1	Steroids and Steroid Derivatives	0	0	1225-01-0
263	1-Butanol	HMDB	Endo	0.080002292	1	Alcohols and Polyols	0	1	71-36-3
27476	1-Methyladenosine	HMDB	Endo	0.099998509	1	Purine Nucleosides and Analogues	0	0	15763-06-1
96373	1-Methylguanosine	HMDB	Endo	0.046000639	1	Purine Nucleosides and Analogues	0	0	2140-65-0
3614	1-Methylhistamine	HMDB	Endo	0.000339988	1	Azoles	0	1	501-75-7
92105	1-Methylhistidine	HMDB	Endo	11.43066893	6	Amino Acids and Derivatives	0	0	332-80-9
65095	1-Methylinosine	HMDB	Endo	0.065343324	5	Purine Nucleosides and Analogues	0	0	2140-73-0
457	1-Methylnicotinamide	HMDB	Endo	0.42998713	1	Pyridines and Derivatives	1	8	3106-60-3
7408	1-Phenylethylamine	HMDB	Endo	0.013999971	1	Phenylmethylamines	0	0	98-84-0
18134	2-(N-Ethyl-perfluorooctane sulfonamido) acetic acid	NHANES	Pollut	0.001045876	1	Perfluorinated Compounds	0	0	1336-61-4
.	2-(N-Methyl-perfluorooctane sulfonamido) acetic acid	NHANES	Pollut	0.00065728	4	Perfluorinated Compounds	.	.	.
225936	2,3-Butanediol	HMDB	Food	5.253528783	2	Alcohols and Polyols	0	1	24347-58-8
53477747	2,3-Dinor-6-keto-prostaglandin F1 a	HMDB	Endo	3.80009E-05	1	Keto-Acids and Derivatives	0	0	.
1491	2,4-Dihydroxybenzoic acid	HMDB	Endo	0.13623145	8	Benzoic Acid and Derivatives	0	0	89-86-1
192742	2,4-Dihydroxybutanoic acid	HMDB	Endo	1.999905641	1	Hydroxy Acids and Derivatives	0	0	1518-62-3

PC_CID	Chemical	Dataset	Source	Concentration (µM)	No. studies	Class	PubMed	Biosys	CAS
9338	2,6-dihydroxybenzoic acid	HMDB	Endo	0.012152748	8	Benzoic Acid and Derivatives	0	0	303-07-1
15109	2,6-Dimethoxybenzoic acid	HMDB	Endo	0.171392334	8	Benzoic Acid and Derivatives	0	0	1466-76-8
92747	20a-Dihydroprogesterone	HMDB	Endo	0.020215614	4	Steroids and Steroid Derivatives	0	0	145-14-2
35027640	20-HETE ethanolamide	HMDB	Endo	0.020800048	1	Fatty Amides	0	0	79551-86-3
5283157	20-Hydroxyeicosatetraenoic acid	HMDB	Endo	0.051139402	4	Eicosanoids	3	7	79551-86-3
440709	21-Deoxycortisol	HMDB	Endo	0.001069996	1	Steroids and Steroid Derivatives	0	1	641-77-0
6434253	24,25-Dihydroxyvitamin D	HMDB	Food	0.006480873	2	Prenol Lipids	1	0	40013-87-4
121948	24-Hydroxycholesterol	HMDB	Food	0.001799944	1	Steroids and Steroid Derivatives	0	10	474-73-7
53477727	25,26-dihydroxyvitamin D	HMDB	Food	0.001564639	3	Prenol Lipids	0	0	29261-12-9
22833566	25-Hydroxyvitamin D2	NHANES	Food	1.387772578	1	Steroids and Steroid Derivatives	17	0	21343-40-8
99470	27-Hydroxycholesterol	HMDB	Endo	0.299991841	1	Steroids and Steroid Derivatives	0	12	13095-61-9
227	2-Aminobenzoic acid	HMDB	Drug	0.000999955	1	Benzoic Acid and Derivatives	0	4	118-92-3
6119	2-Aminoisobutyric acid	HMDB	Endo	0.878886073	2	Amino Acids and Derivatives	0	0	62-57-7
5282280	2-Arachidonylglycerol	HMDB	Endo	7.799814879	1	Fatty Amides	0	14	53847-30-6
99823	2-Hydroxy-3-methylbutyric acid	HMDB	Endo	5.886137441	2	Fatty Acids and Conjugates	0	0	4026-18-0
440864	2-Hydroxybutyric acid	HMDB	Endo	54.00086149	1	Hydroxy Acids and Derivatives	0	1	3347-90-8
440623	2-Hydroxyestrone	HMDB	Endo	0.000201988	2	Steroids and Steroid Derivatives	5	2	362-06-1
1000	2-Hydroxyphenethylamine	HMDB	Endo	0.001907809	2	Phenethylamines	0	0	7568-93-6
96	2-Ketobutyric acid	HMDB	Endo	5.594589668	2	Keto-Acids and Derivatives	1	18	541-50-4,4387-93-3
7052	2-Methoxybenzoic acid	HMDB	Food	0.696143125	8	Benzoic Acid and Derivatives	0	0	91-52-1
66414	2-Methoxyestradiol	HMDB	Drug	0.009999702	1	Steroids and Steroid Derivatives	0	2	362-07-2
439681	2-Methylcitric acid	HMDB	Endo	0.07681157	2	Carboxylic Acids and Derivatives	0	1	6061-96-7
53481667	2-Octenoylcarnitine	HMDB	Endo	0.200007583	1	Fatty Acid Esters	0	0	.
558	2-Oxoarginine	HMDB	Endo	0.120416345	2	Fatty Acids and Conjugates	0	3	3715-10-4
74563	2-Oxovaleric acid	HMDB	Endo	12.89964769	1	Keto-Acids and Derivatives	0	0	1821-02-9
19	2-Pyrocatechuic acid	HMDB	Endo	0.129005531	1	Benzoic Acid and Derivatives	0	0	303-38-8
12025	2-Pyrrolidinone	HMDB	Endo	0.097500283	1	Pyrrolidines	0	0	616-45-5
64860	3-(3,4,5-Trimethoxyphenyl)propanoic acid	HMDB	Food	0.019854988	7	Phenols and Derivatives	0	0	25173-72-2
439435	3-(3,4-Dihydroxyphenyl)lactic acid	HMDB	Endo	6.199694397	1	Benzyl Alcohols and Derivatives	0	1	23028-17-3
16848	3-(3,4-Dimethoxyphenyl)-2-propenoic acid	HMDB	Food	0.535261429	7	Cinnamic Acid Derivatives	0	0	14737-88-3
91	3-(3-Hydroxyphenyl)propanoic acid	HMDB	Food	0.07008125	8	Phenols and Derivatives	0	1	621-54-5

PC_CID	Chemical	Dataset	Source	Concentration (µM)	No. studies	Class	PubMed	Biosys	CAS
547	3,4-Dihydroxybenzeneacetic acid	HMDB	Food	0.010628672	7	Phenols and Derivatives	0	6	102-32-9
348154	3,4-Dihydroxyhydrocinnamic acid	HMDB	Food	0.031688546	9	Phenols and Derivatives	0	1	1078-61-1
85782	3,4-Dihydroxymandelic acid	HMDB	Endo	0.01099956	1	Phenols and Derivatives	0	3	775-01-9
91528	3,4-Dihydroxyphenylglycol	HMDB	Endo	0.005999975	1	Phenols and Derivatives	1	3	28822-73-3,3343-19-9,109835-90-7
74709	3,4-O-Dimethylgallic acid	HMDB	Food	0.03180601	8	Benzoic Acid and Derivatives	0	0	1916-08-1
7424	3,5-dihydroxybenzoic acid	HMDB	Food	0.101550405	8	Benzoic Acid and Derivatives	0	0	99-10-5
9305	3,5-Diiodo-L-tyrosine	HMDB	Endo	0.003600077	1	Amino Acids and Derivatives	0	8	300-39-0
7155252	3,5-Diiodothyronine	HMDB	Endo	1.60003E-05	1	Amino Acids and Derivatives	0	1	534-51-0,5563-89-3
24779614	3a-Hydroxy-5b-pregnane-20-one	HMDB	Endo	0.000288447	2	Steroids and Steroid Derivatives	0	0	.
64956	3-Aminoisobutanoic acid	HMDB	Endo	0.878886073	2	Amino Acids and Derivatives	0	3	144-90-1
53477687	3b,17b-Dihydroxyetiocholane	HMDB	Endo	0.119995624	1	Steroids and Steroid Derivatives	0	0	6038-31-9
86	3-Hydroxyanthranilic acid	HMDB	Endo	0.079000587	1	Benzoic Acid and Derivatives	0	5	548-93-6
7420	3-Hydroxybenzoic acid	HMDB	Food	0.166926781	7	Benzoic Acid and Derivatives	0	0	99-06-9
441	3-Hydroxybutyric acid	HMDB	Endo	81.41829483	8	Hydroxy Acids and Derivatives	6	7	300-85-6,625-71-8,26063-00-3
94216	3-Hydroxydodecanoic acid	HMDB	Endo	0.299991841	1	Fatty Acids and Conjugates	0	0	1883-13-2
181976	3-Hydroxyglutaric acid	HMDB	Endo	0.150002998	1	Hydroxy Acids and Derivatives	0	0	638-18-6
69362	3-Hydroxyisovaleric acid	HMDB	Endo	4.000022556	1	Hydroxy Acids and Derivatives	1	0	625-08-1
12122	3-Hydroxyphenylacetic acid	HMDB	Food	0.232538378	9	Phenylacetic Acid Derivatives	0	2	621-37-4
107802	3-Hydroxyvaleric acid	HMDB	Endo	1	1	Hydroxy Acids and Derivatives	0	0	10237-77-1
3744	3-Indolepropionic acid	HMDB	Endo	0.480994232	1	Indoles	0	0	830-96-6
3035420	3-Methoxy-4-Hydroxyphenylglycol sulfate	HMDB	Endo	0.090004105	1	Phenols and Derivatives	0	0	71324-20-4
11461	3-Methoxybenzoic acid	HMDB	Food	0.242804181	8	Benzoic Acid and Derivatives	0	0	586-38-9
1669	3-Methoxytyramine	HMDB	Endo	0.002499911	1	Phenols and Derivatives	1	3	554-52-9
9307	3-Methoxytyrosine	HMDB	Endo	0.089001683	1	Amino Acids and Derivatives	0	1	300-48-1
47	3-Methyl-2-oxovaleric acid	HMDB	Endo	20.21449659	2	Fatty Acids and Conjugates	0	0	1460-34-0
69520	3-Methylhistamine	HMDB	Endo	0.01099956	1	Azoles	0	0	644-42-8
64969	3-Methylhistidine	HMDB	Endo	2.845958734	2	Amino Acids and Derivatives	0	1	368-16-1
10455	3-Methylsulfinylpropyl isothiocyanate	HMDB	Food	0.090004105	1	Sulfoxides	0	0	505-44-2
65124	3-Nitrotyrosine	HMDB	Endo	0.85001609	1	Amino Acids and Derivatives	13	2	621-44-3,3604-79-3
439168	3-Phosphoglyceric acid	HMDB	Endo	47.20028827	1	Sugar Acids and Derivatives	0	26	591-57-1

PC_CID	Chemical	Dataset	Source	Concentration (µM)	No. studies	Class	PubMed	Biosys	CAS
4615299	3-Pyridylacetic acid	HMDB	Endo	2.469955168	1	Pyridines and Derivatives	0	2	501-81-5
1549098	3-Sulfinoalanine	HMDB	Endo	1.380022771	1	Amino Acids and Derivatives	0	7	1115-65-7
22833586	3-trans,5-cis-Octadienoyl-CoA	HMDB	Endo	0.037500538	1	Purine Nucleotides	0	0	214769-63-8
133246	4a-Carbinolamine tetrahydrobiopterin	HMDB	Endo	4.17995299	1	Pteridines and Derivatives	0	2	79647-29-3
18189	4-Acetamidobutanoic acid	HMDB	Endo	0.500023591	1	Fatty Acids and Conjugates	0	3	3025-96-5
2148	4-Aminohippuric acid	HMDB	Endo	18.00050837	1	Amino Acids and Derivatives	2	5	554-52-9
403	4-Aminophenol	HMDB	Endo	10.00014907	1	Phenols and Derivatives	0	0	123-30-8
12086	4-Ethylbenzoic acid	HMDB	Pollut	1.594085153	8	Benzoic Acid and Derivatives	0	0	619-64-7
31242	4-Ethylphenol	HMDB	Food	2.165822073	8	Phenols and Derivatives	0	0	123-07-9
500	4-Guanidinobutanoic acid	HMDB	Endo	0.084500316	2	Amino Acids and Derivatives	0	2	463-00-3
31246	4-Heptanone	HMDB	Endo	0.029998737	1	Carbonyl Compounds	0	0	123-19-3
135	4-Hydroxybenzoic acid	HMDB	Food	3.269378808	8	Benzoic Acid and Derivatives	0	8	99-96-7
3037032	4-Hydroxybutyric acid	HMDB	Endo	47.99995148	1	Hydroxy Acids and Derivatives	0	5	502-85-2,52352-27-9,1320-61-2
107669	4-Hydroxydebrisoquine	HMDB	Drug	0.21699087	1	Quinolines and Derivatives	0	5	59333-79-8
102526	4-Hydroxyphenyl-2-propionic acid	HMDB	Food	0.168099368	8	Phenols and Derivatives	0	0	938-96-5
979	4-Hydroxyphenylpyruvic acid	HMDB	Endo	0.369982342	1	Benzyl Alcohols and Derivatives	0	9	156-39-8
699414	4-Methoxycinnamic acid	HMDB	Food	0.716770194	8	Cinnamic Acid Derivatives	0	0	830-09-1
7690	4-Methoxyphenylacetic acid	HMDB	Endo	0.143977247	8	Phenols and Derivatives	0	0	104-01-8
78016	4-O-Methylgallic acid	HMDB	Food	0.036563676	8	Benzoic Acid and Derivatives	0	0	4319-02-2
6723	4-Pyridoxic acid	NHANES	Endo	0.031901572	1	Pyridines and Derivatives	1	6	82-82-6,524-07-2
134	4-Trimethylammoniobutanoic acid	HMDB	Endo	10.00014907	1	Fatty Acids and Conjugates	0	2	407-64-7
9548565	5,10-Methylene-THF	HMDB	Endo	0.009999702	1	Pteridines and Derivatives	0	15	31690-11-6
5283158	5,15-DiHETE	HMDB	Endo	0.003179918	3	Eicosanoids	0	0	82200-87-1
5283142	5,6-DHET	HMDB	Endo	0.010745158	4	Eicosanoids	0	8	213382-49-1
5283202	5,6-Epoxy-8,11,14-eicosatrienoic acid	HMDB	Endo	0.000877529	2	Fatty Acids and Conjugates	0	14	81246-84-6
4028	5,8,11-Eicosatrienoic acid	HMDB	Endo	0.249798671	3	Fatty Acids and Conjugates	0	0	20590-32-3
242332	5a-Androstane-3b,17b-diol	HMDB	Endo	0.000148995	1	Steroids and Steroid Derivatives	0	0	571-20-0
92810	5a-Pregnane-3,20-dione	HMDB	Endo	0.030669089	2	Steroids and Steroid Derivatives	0	1	566-65-4
6439678	5-HEPE	HMDB	Endo	0.019732268	4	Eicosanoids	0	0	92008-51-0
5280733	5-HETE	HMDB	Endo	0.142573161	8	Eicosanoids	0	7	70608-72-9

PC_CID	Chemical	Dataset	Source	Concentration (µM)	No. studies	Class	PubMed	Biosys	CAS
1826	5-Hydroxyindoleacetic acid	HMDB	Endo	0.051601734	1	Indoles	5	9	54-16-0,1321-73-9,113303-91-6
439280	5-Hydroxy-L-tryptophan	HMDB	Endo	0.017999703	1	Amino Acids and Derivatives	0	11	4350-09-8
3032849	5-Hydroxylysine	HMDB	Endo	2.50002317	1	Amino Acids and Derivatives	0	0	1190-94-9
9061	5-Hydroxytryptophol	HMDB	Endo	0.000900014	1	Indoles	0	3	154-02-9,120465-13-6
5283159	5-KETE	HMDB	Endo	0.013893975	3	Fatty Acids and Conjugates	0	12	126432-17-5
1832	5-Methoxydimethyltryptamine	HMDB	Endo	0.210010027	1	Alkaloids and Derivatives	0	0	1019-45-0
12835	5-Methoxytryptophol	HMDB	Endo	0.000151564	5	Indoles	0	3	712-09-4
439234	5-Methyltetrahydrofolic acid	HMDB	Endo	0.299991841	1	Pteridines and Derivatives	9	5	134-35-0
5312400	5-Tetradecenoic acid	HMDB	Endo	1.500052339	1	Fatty Acids and Conjugates	0	0	544-66-1
1864	6-Hydroxymelatonin	HMDB	Endo	0.000239993	1	Indoles	0	5	2208-41-5
5280888	6-Keto-prostaglandin F1a	HMDB	Endo	0.005644351	4	Eicosanoids	16	6	58962-34-8
5283129	6-trans-12-epi-Leukotriene B4	HMDB	Endo	0.000146088	2	Eicosanoids	0	4	71548-19-1
5283128	6-trans-Leukotriene B4	HMDB	Endo	0.00176201	3	Eicosanoids	0	4	71652-82-9
53477732	7a-Hydroxycholesterol	HMDB	Endo	0.179999717	1	Steroids and Steroid Derivatives	0	0	.
473141	7b-Hydroxycholesterol	HMDB	Endo	0.01099956	1	Steroids and Steroid Derivatives	3	0	566-27-8
439423	7-Dehydrocholesterol	HMDB	Endo	4.999810441	1	Steroids and Steroid Derivatives	0	10	434-16-2
91474	7-Ketocholesterol	HMDB	Endo	0.033001575	1	Steroids and Steroid Derivatives	2	4	566-28-9
11361	7-Methylguanine	HMDB	Endo	0.129005531	1	Imidazopyrimidines	0	0	578-76-7
5280581	8,11,14-Eicosatrienoic acid	NHANES	Endo	151.0030447	1	Fatty Acids and Conjugates	0	13	1783-84-2
5283144	8,9-DiHETrE	HMDB	Endo	0.01099846	4	Eicosanoids	0	8	192461-96-4
5283203	8,9-Epoxyeicosatrienoic acid	HMDB	Endo	0.627005479	1	Eicosanoids	0	14	81246-85-7
129846	8-Dehydrocholesterol	HMDB	Endo	0.649988896	1	Steroids and Steroid Derivatives	0	0	70741-38-7
5283154	8-HETE	HMDB	Endo	0.025601611	4	Eicosanoids	0	3	98462-03-4
119315	8-Hydroxyguanine	HMDB	Endo	0.000100004	1	Imidazopyrimidines	1	5	82014-86-6
65131	8-Hydroxyguanosine	HMDB	Endo	0.001419998	1	Purine Nucleosides and Analogues	1	0	3868-31-3
5282263	8-Isoprostaglandin F2a	HMDB	Endo	0.00030106	5	Eicosanoids	41	2	27415-26-5
107873	8-Isoprostane	HMDB	Endo	0.000301482	3	Cycloalkanes	41	0	155976-51-5
16061060	9(10)-EpODE	HMDB	Endo	0.192549888	3	Fatty Acids and Conjugates	0	0	.
9548877	9(S)-HPODE	HMDB	Endo	5.140241177	1	Lineolic Acids and Derivatives	0	2	29774-12-7
5282965	9,10,13-TriHOME	HMDB	Endo	0.067333325	3	Fatty Acids and Conjugates	0	1	29907-57-1

PC_CID	Chemical	Dataset	Source	Concentration (µM)	No. studies	Class	PubMed	Biosys	CAS
9966640	9,10-DHOME	HMDB	Endo	4.772641857	3	Fatty Acids and Conjugates	0	1	.
16061066	9,10-DiHODE	HMDB	Endo	0.085949102	3	Lineolic Acids and Derivatives	0	0	.
6246154	9,10-Epoxyoctadecenoic acid	HMDB	Endo	0.261113526	3	Fatty Acids and Conjugates	0	0	.
9858729	9,12,13-TriHOME	HMDB	Endo	0.141126306	3	Fatty Acids and Conjugates	0	1	.
53481687	9,12-Hexadecadienoylcarnitine	HMDB	Endo	0.009999702	1	Fatty Acid Esters	0	0	.
449171	9-cis-Retinoic acid	HMDB	Endo	0.004849886	2	Prenol Lipids	0	12	5300-03-8
53481651	9-Decenoylcarnitine	HMDB	Endo	0.169992663	1	Fatty Acid Esters	0	0	.
5282796	9E,11E-Octadecadienoic acid	HMDB	Endo	19.29990165	1	Lineolic Acids and Derivatives	0	0	1839-11-8
5312978	9-HETE	HMDB	Endo	0.018935818	4	Eicosanoids	0	0	79495-85-5
53481653	9-Hexadecenoylcarnitine	HMDB	Endo	0.029998737	1	Fatty Acid Esters	0	0	.
5312830	9-HODE	HMDB	Endo	0.303158351	4	Lineolic Acids and Derivatives	0	9	73543-67-6
53480359	9-HOTE	HMDB	Endo	0.167093791	3	Lineolic Acids and Derivatives	0	0	.
9839084	9-OxoODE	HMDB	Endo	0.313392149	3	Lineolic Acids and Derivatives	0	1	.
16061061	A-12(13)-EpODE	HMDB	Endo	0.043265488	3	Fatty Acids and Conjugates	0	0	.
177	Acetaldehyde	HMDB	Food	1	1	Carbonyl Compounds	3	24	75-07-0,78944-68-0,12772-68-8
83944	Acetaminophen glucuronide	HMDB	Drug	30.19873391	1	Sugar Acids and Derivatives	0	4	16110-10-4
176	Acetic acid	HMDB	Endo	35.69105153	2	Carboxylic Acids and Derivatives	1	52	64-19-7,1563-79-7,157090-22-7,207004-55-5,248587-59-9,68475-71-8,77671-22-8,9035-69-2
6971017	Acetoacetic acid	HMDB	Endo	29.19799376	2	Keto-Acids and Derivatives	0	13	141-81-1
447765	Acetoin	HMDB	Food	1.060032958	1	Acyloins	0	1	78183-56-9
12035	Acetylcysteine	HMDB	Endo	4.000022556	1	Amino Acids and Derivatives	19	0	616-91-1,7696-05-1
10972	Acetylglycine	HMDB	Endo	87.33925342	2	Amino Acids and Derivatives	0	0	543-24-8
171161	Acetyl-N-formyl-5-methoxykynurenamine	HMDB	Endo	6.50015E-05	1	Benzoic Acid and Derivatives	0	4	52450-38-1
750	Adenine	HMDB	Endo	0.438191171	2	Imidazopyrimidines	30	83	56-40-6,25718-94-9,18875-39-3,52955-63-2,57678-19-0,848646-45-7,87867-94-5,112898-03-0
60961	Adenosine	HMDB	Endo	0.376175308	5	Purine Nucleosides and Analogues	56	45	58-61-7,30143-02-3,46946-45-6,46969-16-8
2024	Adenosine 2',3'-cyclic phosphate	HMDB	Endo	0.025998927	1	Purine Nucleotides	0	0	634-01-5

PC_CID	Chemical	Dataset	Source	Concentration (μM)	No. studies	Class	PubMed	Biosys	CAS
6083	Adenosine monophosphate	HMDB	Endo	12.25826006	4	Purine Nucleotides	7	153	61-19-8,13270-66-1,162756-82-3,24937-83-5,34051-12-2,47286-65-7,47287-97-8,53624-78-5,55036-25-4,67583-85-1,697214-87-2
5957	Adenosine triphosphate	HMDB	Endo	1894.480618	3	Purine Nucleotides	19	707	56-65-5,10168-83-9,16488-07-6,34369-07-8,51569-41-6,71800-44-7,84412-18-0,896506-78-8
196	Adipic acid	HMDB	Food	0.090004105	1	Carboxylic Acids and Derivatives	0	1	124-04-9
6022	ADP	HMDB	Endo	160.0041896	1	Purine Nucleotides	76	626	58-64-0,84412-16-8,20398-34-9,18389-49-6,896506-82-4,905904-58-7
5497181	Adrenic acid	NHANES	Endo	25.00060439	1	Fatty Acids and Conjugates	0	10	2091-25-0
5282273	Adrenoyl ethanolamide	HMDB	Endo	1.630032576	1	Fatty Amides	0	0	150314-35-5
186907	Aflatoxin B1	HMDB	Food	0.000900014	1	Aflatoxins	17	5	1162-65-8,11003-08-0,13214-11-4,27261-02-5
5839	Aldosterone	HMDB	Endo	9.0941E-05	3	Steroids and Steroid Derivatives	94	10	52-39-1,10328-70-8,152-04-5
204	Allantoin	HMDB	Endo	2.099921578	1	Imidazolidines	0	0	97-59-6
92787	Alloepipregnanolone	HMDB	Endo	0.000714163	2	Steroids and Steroid Derivatives	0	0	516-55-2
440700	Allopregnanolone	HMDB	Endo	0.001259929	3	Steroids and Steroid Derivatives	0	2	128-20-1
456569	Allopurinol riboside	HMDB	Endo	4.999810441	1	Pyrazolopyrimidine Nucleosides and Nucleotides	0	0	16220-07-8
5971	Allyl isothiocyanate	HMDB	Food	0.1400018	1	Isothiocyanates	0	6	57-06-7
4369188	Alpha-Carotene	NHANES	Food	0.05141116	1	Prenol Lipids	22	2	7488-99-5,432-70-2,24319-81-1,52646-96-5
9943542	Alpha-CEHC	HMDB	Food	0.013000077	1	Benzopyrans	0	1	4072-32-6
82400	Alpha-D-Glucose 1,6-bisphosphate	HMDB	Endo	98.00318714	1	Monosaccharides	0	1	10139-18-1
5282945	Alpha-dimorphecolic acid	HMDB	Endo	0.006800222	1	Lineolic Acids and Derivatives	0	0	15514-85-9
11671	Alpha-Hydroxyisobutyric acid	HMDB	Pollut	7.576110945	2	Hydroxy Acids and Derivatives	0	0	594-61-6
49	Alpha-ketoisovaleric acid	HMDB	Endo	11.000052	1	Fatty Acids and Conjugates	0	9	759-05-7
5280934	Alpha-Linolenic acid	NHANES	Food	63.09868946	1	Lineolic Acids and Derivatives	42	30	463-40-1
5283449	Alpha-Linolenoyl ethanolamide	HMDB	Endo	0.117996537	1	Fatty Amides	0	0	57086-93-8

PC_CID	Chemical	Dataset	Source	Concentration (μM)	No. studies	Class	PubMed	Biosys	CAS
92258	Alpha-N-Phenylacetyl-L-glutamine	HMDB	Endo	3.340097506	1	Amino Acids and Derivatives	0	7	28047-15-6
14985	Alpha-Tocopherol	NHANES	Food	25.30747989	1	Prenol Lipids	266	5	59-02-9,1406-18-4,11105-14-9,16826-11-2,181591-70-8,25094-97-7,4072-33-7
5282347	Alpha-Tocotrienol	HMDB	Food	4.229991569	1	Prenol Lipids	1	0	58864-81-6,1721-51-3
104727	Aluminum	HMDB	Food	0.184999899	1	Homogeneous Post-transition Metal Compounds	5	0	22537-23-1
215	Aminoacetone	HMDB	Endo	59.99732633	1	Carbonyl Compounds	0	1	298-08-8
469	Aminoadipic acid	HMDB	Endo	1.999905641	1	Amino Acids and Derivatives	0	0	542-32-5
564	Aminocaproic acid	HMDB	Drug	1.999905641	1	Fatty Acids and Conjugates	0	1	60-32-2,87867-96-7,93208-38-9
222	Ammonia	HMDB	Endo	32.94040435	4	Other Non-metal Organides	9	130	7664-41-7,3589-23-9,13981-22-1,17778-88-0,208990-07-2,214478-05-4,558443-52-0,63993-67-9,8007-57-6
2181	Anabasine	HMDB	Pollut	0.003099855	1	Alkaloids and Derivatives	0	0	13078-04-1
5281969	Anandamide	HMDB	Endo	0.42100945	3	Fatty Amides	1	7	94421-68-8
440114	Androstanedione	HMDB	Endo	0.000360002	1	Steroids and Steroid Derivatives	0	1	1229-12-5
6128	Androstenedione	HMDB	Endo	0.002835321	4	Steroids and Steroid Derivatives	62	18	63-05-8,104534-78-3,117598-81-9
5879	Androsterone	HMDB	Endo	0.008831769	2	Steroids and Steroid Derivatives	3	1	53-41-8
443078	Androsterone glucuronide	HMDB	Endo	0.056909101	4	Steroids and Steroid Derivatives	2	1	3602-09-3
159663	Androsterone sulfate	HMDB	Endo	11.09949931	1	Steroids and Steroid Derivatives	0	0	2479-86-9
112072	Anserine	HMDB	Food	41.00114528	1	Peptidomimetics	0	3	584-85-0
104894	Antimony	HMDB	Pollut	0.003799826	1	Homogeneous Metalloid Compounds	2	0	7440-36-0
3082883	APGPR Enterostatin	HMDB	Endo	1.39724159	4	Peptides	0	0	117830-79-2
5280960	Apigenin	HMDB	Food	0.126995961	1	Flavonoids	1	1	25515-46-2
6449885	Aquacobalamin	HMDB	Food	0.000289835	2	Tetrapyrroles and Derivatives	0	0	13422-52-1
10467	Arachidic acid	NHANES	Food	23.39915709	1	Fatty Acids and Conjugates	0	5	506-30-9
444899	Arachidonic acid	NHANES	Endo	776.0368452	1	Fatty Acids and Conjugates	48	66	506-32-1,93444-49-6
160437	Argininic acid	HMDB	Endo	0.200007583	1	Amino Acids and Derivatives	0	0	157-07-3
104734	Arsenic	HMDB	Pollut	0.017366712	2	Homogeneous Metalloid Compounds	33	1	22541-54-4

PC_CID	Chemical	Dataset	Source	Concentration (μM)	No. studies	Class	PubMed	Biosys	CAS
2244	Aspirin	HMDB	Drug	26.4988146	1	Benzoic Acid and Derivatives	515	1	50-78-2,11126-35-5,11126-37-7,2349-94-2,26914-13-6,98201-60-6
5368397	Astaxanthin	HMDB	Food	0.091996937	1	Prenol Lipids	0	1	472-61-7
2249	Atenolol	HMDB	Drug	3.750165604	1	Ethers	113	0	29122-68-7,60966-51-0,106020-65-9
60823	Atorvastatin	HMDB	Drug	0.009999702	1	Anilides	206	0	134523-00-5
12795736	Avenasterol	HMDB	Food	1.450052844	1	Steroids and Steroid Derivatives	0	1	23290-26-8
2266	Azelaic acid	HMDB	Endo	26.99900464	1	Carboxylic Acids and Derivatives	0	0	123-99-9
104810	Barium	HMDB	Pollut	0.008400198	1	Homogeneous Alkaline Earth Metal Compounds	.	.	#N/A
154083	BDE100	NHANES	Pollut	4.65167E-05	1	Bromodiphenyl Ethers	0	0	189084-64-8
155166	BDE153	NHANES	Pollut	5.90929E-05	1	Bromodiphenyl Ethers	0	0	68631-49-2
95170	BDE47	NHANES	Pollut	0.000242648	1	Bromodiphenyl Ethers	0	0	5436-43-1
36159	BDE99	NHANES	Pollut	8.14354E-05	1	Bromodiphenyl Ethers	0	0	60348-60-9
8215	Behenic acid	NHANES	Endo	69.29688807	1	Fatty Acids and Conjugates	0	1	112-85-6
241	Benzene	NHANES	Pollut	0.000353545	3	Aromatic Homomonocyclic Compounds	7	6	71-43-2,27271-55-2,8030-30-6,174973-66-1,26181-88-4,54682-86-9
243	Benzoic acid	HMDB	Food	15.76196879	8	Benzoic Acid and Derivatives	0	9	65-85-0
2353	Berberine	HMDB	Drug	0.001299988	1	Alkaloids and Derivatives	0	0	2086-83-1
107649	Beryllium	HMDB	Pollut	0.026601172	1	Homogeneous Alkaline Earth Metal Compounds	13	0	7440-41-7
239	Beta-Alanine	HMDB	Endo	2.541107476	5	Amino Acids and Derivatives	5	23	107-95-9,87867-95-6
5280489	Beta-Carotene	NHANES	Food	0.225372656	1	Prenol Lipids	194	10	7235-40-7,116-32-5,31797-85-0
5281235	Beta-Cryptoxanthin	NHANES	Food	0.13927568	1	Prenol Lipids	19	1	472-70-8
247	Betaine	HMDB	Endo	58.32320257	3	Amino Acids and Derivatives	6	22	107-43-7,11042-12-9,24980-93-6,45631-77-4,590-30-7
439734	Beta-Leucine	HMDB	Endo	4.799923595	1	Amino Acids and Derivatives	0	0	5699-54-7
222284	Beta-Sitosterol	HMDB	Food	22.8008998	1	Steroids and Steroid Derivatives	13	1	83-46-5,68555-08-8,5779-62-4
8989	Beta-tocopherol	HMDB	Food	0.200007583	1	Prenol Lipids	0	2	148-03-8

PC_CID	Chemical	Dataset	Source	Concentration (µM)	No. studies	Class	PubMed	Biosys	CAS
5280352	Bilirubin	HMDB	Endo	11.64876307	3	Tetrapyrroles and Derivatives	167	6	11053-42-2,114-24-9,18422-02-1,188341-33-5,19245-52-4,39372-61-7,493-86-7,55527-40-7,856927-39-4,917-01-1,93891-87-3
12646210	Biopterin	HMDB	Endo	0.017000713	1	Pteridines and Derivatives	0	0	13039-82-2
171548	Biotin	HMDB	Food	0.001745874	2	Thienoimidazolidines	1	14	58-85-5,15720-24-8,56846-45-8,22879-79-4,3672-05-7
86492	Bisnorbiotin	HMDB	Endo	0.001504041	3	Fatty Acids and Conjugates	0	1	16968-98-2
5280644	Bovinic acid	HMDB	Food	2.029927341	1	Lineolic Acids and Derivatives	0	1	2540-56-9
5283660	Brassicasterol	HMDB	Food	0.570010783	1	Steroids and Steroid Derivatives	0	0	474-67-9
6359	Bromodichloromethane	NHANES	Pollut	1.02074E-05	3	Organochlorides	1	0	75-27-4
6834	Brompheniramine	HMDB	Drug	0.024000035	1	Pheniramines	0	0	86-22-6
264	Butyric acid	HMDB	Food	1	1	Fatty Acids and Conjugates	0	16	107-92-6
439829	Butyrylcarnitine	HMDB	Endo	0.263474155	2	Fatty Acid Esters	0	0	25576-40-3
31193	Cadmium	NHANES	Pollut	0.002861813	6	Homogeneous Transition Metal Compounds	30	0	22537-48-0
689043	Caffeic acid	HMDB	Food	0.687564249	9	Cinnamic Acid Derivatives	1	1	331-39-5,501-16-6,71693-97-5,3598-26-3
2519	Caffeine	HMDB	Food	77.99931152	1	Imidazopyrimidines	56	14	58-08-2,71701-02-5,95789-13-2
5283731	Calcidiol	HMDB	Food	0.060319491	3	Prenol Lipids	52	12	19356-17-3,63283-36-3,25631-40-7,1384584-62-6
5280453	Calcitriol	HMDB	Food	7.9775E-05	2	Prenol Lipids	34	14	32222-06-3
271	Calcium	HMDB	Food	2382.724834	3	Homogeneous Alkaline Earth Metal Compounds	379	271	14127-61-8,17787-72-3
23724579	Campestanol	HMDB	Food	2.50002317	1	Steroids and Steroid Derivatives	1	1	474-60-2
173183	Campesterol	HMDB	Food	17.23082619	1	Steroids and Steroid Derivatives	10	2	474-62-4
5281227	Canthaxanthin	HMDB	Food	0.069997203	1	Prenol Lipids	2	1	514-78-3
2969	Capric acid	HMDB	Endo	11.000052	1	Fatty Acids and Conjugates	0	8	334-48-5
8892	Caproic acid	HMDB	Endo	16.99977315	1	Fatty Acids and Conjugates	0	0	142-62-1
119389	Caprylic acid	HMDB	Endo	4.472287399	2	Fatty Acids and Conjugates	0	1	124-07-2,68937-74-6

PC_CID	Chemical	Dataset	Source	Concentration (μM)	No. studies	Class	PubMed	Biosys	CAS
712	Carbon monoxide	HMDB	Endo	158.7451363	2	Aliphatic Acyclic Compounds	6	25	50-00-0,30525-89-4,104512-58-5,104512-63-2,104814-22-4,112068-71-0,53026-80-5,8005-38-7,8006-07-3,8013-13-6
439224	Carnosine	HMDB	Endo	6.539756942	1	Peptidomimetics	1	4	305-84-0
9064	Catechin	HMDB	Food	0.891633594	2	Flavonoids	9	0	154-23-4,159761-73-6,16198-00-8,321-01-7,4211-28-3,5323-80-8,7295-85-4,100786-01-4
6132	CDP	HMDB	Endo	35.99931822	1	Pyrimidine Nucleotides	0	15	63-38-7
99486	CE(14:0)	HMDB	Endo	79.99786925	1	Steroids and Steroid Derivatives	0	0	1989-52-2
53480456	CE(14:1(9Z))	HMDB	Endo	11.21105113	2	Steroids and Steroid Derivatives	0	0	.
246520	CE(16:0)	HMDB	Endo	237.9355884	4	Steroids and Steroid Derivatives	0	0	601-34-3
22833543	CE(16:1(9Z))	HMDB	Endo	114.8009777	2	Steroids and Steroid Derivatives	0	0	16711-66-3
118246	CE(18:0)	HMDB	Endo	47.03537603	2	Steroids and Steroid Derivatives	0	1	35602-69-8
53477793	CE(18:1(11Z))	HMDB	Endo	153.5459698	2	Steroids and Steroid Derivatives	0	0	.
5283632	CE(18:1(9Z))	HMDB	Endo	612.7763351	2	Steroids and Steroid Derivatives	1	0	303-43-5
5287939	CE(18:2(9Z,12Z))	HMDB	Endo	1655.734507	2	Steroids and Steroid Derivatives	2	0	604-33-1,100339-48-8,126346-09-6,55102-87-9
53480458	CE(18:3(6Z,9Z,12Z))	HMDB	Endo	58.97419645	2	Steroids and Steroid Derivatives	0	0	.
6436907	CE(18:3(9Z,12Z,15Z))	HMDB	Endo	58.89757981	2	Steroids and Steroid Derivatives	0	0	2545-22-4
16061336	CE(20:0)	HMDB	Endo	6.144761929	2	Steroids and Steroid Derivatives	0	1	2573-03-7
53477794	CE(20:1(11Z))	HMDB	Endo	1.260993623	2	Steroids and Steroid Derivatives	0	0	70832-37-0
53477891	CE(20:2(6Z,9Z))	HMDB	Endo	12.06368862	2	Steroids and Steroid Derivatives	0	0	77715-45-8
53480461	CE(20:3(5Z,8Z,11Z))	HMDB	Endo	9.46593912	2	Steroids and Steroid Derivatives	0	0	.
53477892	CE(20:3(8Z,11Z,14Z))	HMDB	Endo	24.46882847	2	Steroids and Steroid Derivatives	0	0	7274-08-0
6479222	CE(20:4(5Z,8Z,11Z,14Z)	HMDB	Endo	215.2499692	2	Steroids and Steroid Derivatives	0	1	604-34-2
53480459	CE(20:4(8Z,11Z,14Z,17Z))	HMDB	Endo	24.19388705	2	Steroids and Steroid Derivatives	0	0	.
53477889	CE(20:5(5Z,8Z,11Z,14Z,17Z)	HMDB	Endo	39.07176174	1	Steroids and Steroid Derivatives	0	0	74892-97-0
16061339	CE(22:0)	HMDB	Endo	4.98383662	2	Steroids and Steroid Derivatives	0	1	61510-09-6
53480460	CE(22:1(13Z))	HMDB	Endo	4.312854578	2	Steroids and Steroid Derivatives	0	0	.
53477893	CE(22:2(13Z,16Z))	HMDB	Endo	0.387747858	3	Steroids and Steroid Derivatives	0	0	.
53480462	CE(22:5(4Z,7Z,10Z,13Z,16Z))	HMDB	Endo	3.279857578	1	Steroids and Steroid Derivatives	0	0	.

PC_CID	Chemical	Dataset	Source	Concentration (µM)	No. studies	Class	PubMed	Biosys	CAS
53480463	CE(22:5(7Z,10Z,13Z,16Z,19Z))	HMDB	Endo	3.230057697	1	Steroids and Steroid Derivatives	0	0	.
53477890	CE(22:6(4Z,7Z,10Z,13Z,16Z,19Z))	HMDB	Endo	26.49086615	2	Steroids and Steroid Derivatives	0	0	70110-50-8
53480464	CE(24:0)	HMDB	Endo	0.91998308	1	Steroids and Steroid Derivatives	0	0	.
2662	Celecoxib	HMDB	Drug	0.025699082	1	Phenylpyrazoles	20	4	169590-42-5,184007-95-2,194044-54-7
5283563	Ceramide (d18:1/9Z-18:1)	HMDB	Endo	8.999778807	1	Sphingolipids	0	0	104404-17-3
53481046	Ceramide(d18:0/12:0)	HMDB	Endo	0.081049111	14	Sphingolipids	0	0	.
53481052	Ceramide(d18:1/22:1(13Z))	HMDB	Endo	0.128016003	14	Sphingolipids	0	0	.
53245830	Cervonoyl ethanolamide	HMDB	Endo	0.400997535	1	Fatty Acid Esters	0	0	.
10133	Chenodeoxycholic acid	HMDB	Endo	1.150273799	2	Steroids and Steroid Derivatives	2	19	474-25-9
21252312	Chenodeoxycholic acid 3-sulfate	HMDB	Endo	0.099998509	1	Steroids and Steroid Derivatives	0	0	59132-32-0
12544	Chenodeoxycholic acid glycine conjugate	HMDB	Endo	0.060000643	1	Steroids and Steroid Derivatives	0	11	640-79-9
1794427	Chlorogenic acid	HMDB	Food	0.039999033	1	Cyclic Alcohols and Derivatives	2	0	327-97-9
5997	Cholesterol	HMDB	Endo	416.5056388	4	Steroids and Steroid Derivatives	4499	73	57-88-5,80356-14-5,209124-38-9,218965-24-3,22243-67-0,262418-13-3,378185-03-6,676322-57-9,732297-95-9,793670-51-6,80356-33-8,849593-11-9,856708-55-9
65076	Cholesterol sulfate	HMDB	Endo	5.400005651	1	Steroids and Steroid Derivatives	0	5	1256-86-6
221493	Cholic acid	HMDB	Endo	0.720002928	1	Steroids and Steroid Derivatives	0	24	81-25-4,73163-53-8
305	Choline	HMDB	Food	8.898653739	4	Alcohols and Polyols	13	38	62-49-7,123-41-1,139741-81-4
24766	Chondroitin sulfate	HMDB	Endo	9.580214671	1	Disaccharides	9	2	9007-28-7,11120-14-2,25322-46-7,56480-79-6,9046-20-2,9062-29-7,9088-44-2
27668	Chromium	HMDB	Pollut	0.004806433	2	Homogeneous Transition Metal Compounds	9	1	16065-83-1
444539	Cinnamic acid	HMDB	Food	0.190215051	8	Cinnamic Acid Derivatives	0	2	140-10-3
22833575	cis-5-Tetradecenoylcarnitine	HMDB	Endo	0.060000643	1	Fatty Acid Esters	0	0	835598-21-5
6857557	cis-7,7',8,8',11,12-Hexahydro-Carotene	HMDB	Endo	0.302008535	1	Prenol Lipids	0	0	118574-77-9
11722594	Cis-8,11,14,17-Eicosatetraenoic acid	HMDB	Endo	0.007000316	1	Fatty Acids and Conjugates	0	0	2091-26-1
5282761	cis-Vaccenic acid (18:1n-7)	NHANES	Food	145.9990332	1	Fatty Acids and Conjugates	2	0	506-17-2

PC_CID	Chemical	Dataset	Source	Concentration (µM)	No. studies	Class	PubMed	Biosys	CAS
311	Citric acid	HMDB	Endo	124.0643025	3	Carboxylic Acids and Derivatives	7	26	77-92-9,12262-73-6,5077-53-2,6018-92-4,245654-34-6,43136-35-2,623158-96-3,856568-15-5,878903-72-1,890704-54-8,896506-46-0,906507-37-7
9750	Citrulline	HMDB	Endo	36.64584309	3	Amino Acids and Derivatives	10	19	372-75-8
46173708	Cob(I)alamin	HMDB	Endo	0.000300008	1	Tetrapyrroles and Derivatives	0	1	18534-66-2
5460183	Cobalamin	HMDB	Food	0.000244939	2	Prenol Lipids	0	9	18534-66-2
104730	Cobalt	HMDB	Pollut	0.001830072	1	Homogeneous Transition Metal Compounds	4	10	7440-48-4,13981-50-5,16610-75-6,177256-35-8,184637-91-0,195161-79-6
9962735	Coenzyme Q10	HMDB	Endo	0.27049545	5	Prenol Lipids	0	4	992-78-9
23978	Copper	HMDB	Food	18.87616472	6	Homogeneous Transition Metal Compounds	53	17	7440-50-8,13982-06-4,15128-03-7,133353-46-5,133353-47-6,195161-80-9,65555-90-0,72514-83-1
68271	Coproporphyrin I	HMDB	Endo	0.007745835	2	Tetrapyrroles and Derivatives	0	1	531-14-6
321	Coproporphyrin III	HMDB	Endo	0.005999975	1	Tetrapyrroles and Derivatives	0	6	2624-63-7
440776	Coproporphyrinogen I	HMDB	Endo	0.015000076	1	Tetrapyrroles and Derivatives	0	4	31110-56-2
440707	Cortexolone	HMDB	Endo	0.003899917	1	Steroids and Steroid Derivatives	1	12	152-58-9
5753	Corticosterone	HMDB	Drug	0.027000497	1	Steroids and Steroid Derivatives	1	13	50-22-6
5754	Cortisol	HMDB	Endo	0.274171899	3	Steroids and Steroid Derivatives	176	18	50-23-7,8056-08-4,80562-38-5,8063-42-1
222786	Cortisone	HMDB	Endo	0.046019043	3	Steroids and Steroid Derivatives	3	11	53-06-5
854019	Cotinine	NHANES	Pollut	0.000321858	6	Alkaloids and Derivatives	78	2	486-56-6
9815514	Cotinine N-oxide	HMDB	Pollut	1.7E-05	1	Alkaloids and Derivatives	0	0	36508-80-2
323	Coumaric acid	HMDB	Food	0.583798144	8	Cinnamic Acid Derivatives	3	11	91-64-5
5281707	Coumesterol	HMDB	Food	0.012299459	1	Benzopyrans	0	0	479-13-0
586	Creatine	HMDB	Endo	39.2636832	3	Amino Acids and Derivatives	50	8	57-00-1
588	Creatinine	HMDB	Endo	74.47027109	5	Lactams	1654	12	60-27-5,82016-55-5,15231-31-9,45514-66-7
969516	Curcumin	HMDB	Food	0.169992663	1	Curcuminoids	3	1	458-37-7,8024-37-1,15845-47-3,33171-04-9,73729-23-4,79257-48-0,91884-86-5
540	Cyanate	HMDB	Endo	0.044999676	1	Nitriles	0	2	420-05-3

PC_CID	Chemical	Dataset	Source	Concentration (µM)	No. studies	Class	PubMed	Biosys	CAS
768	Cyanide	HMDB	Endo	4.440646616	2	Nitriles	0	6	74-90-8,191234-22-7,341972-31-4
68247	Cyanidin	HMDB	Food	0.02000046	1	Flavonoids	0	0	528-58-5
4289526	Cyanocobalamin	NHANES	Food	0.000368894	1	Tetrapyrroles and Derivatives	347	0	68-19-9
6076	Cyclic AMP	HMDB	Endo	0.008499907	1	Purine Nucleotides	9	126	60-92-4,11002-78-1,54532-48-8
24316	Cyclic GMP	HMDB	Endo	0.00550004	1	Purine Nucleotides	18	24	7665-99-8
439498	Cysteinylglycine	HMDB	Endo	36.22320733	3	Peptides	14	10	19246-18-5
6175	Cytidine	HMDB	Endo	0.158120165	2	Pyrimidine Nucleosides and Analogues	1	15	65-46-3,4395-95-3,494210-74-1,873077-03-3
764	Cytosine	HMDB	Endo	6.400012862	1	Diazines	46	17	73-40-5,11006-44-3,66224-64-4,158475-99-1,15986-36-4,37432-34-1,492-33-1,54435-87-9,69257-39-2,929247-29-0,934585-79-2,936902-27-1
439391	D-2-Hydroxyglutaric acid	HMDB	Endo	0.699982461	1	Hydroxy Acids and Derivatives	0	6	2889-31-8
5281708	Daidzein	HMDB	Food	0.003293844	2	Flavonoids	6	0	486-66-8
83887	D-Aspartic acid	HMDB	Endo	20.49334092	2	Amino Acids and Derivatives	0	5	1783-96-6
65091	dCTP	HMDB	Endo	29.00012093	1	Pyrimidine Nucleotides	0	24	2056-98-6,102783-51-7
3035	DDE	NHANES	Pollut	0.005131281	3	Organo-chlorine Pesticides	36	1	72-55-9,12002-54-9
2966	Debrisoquine	HMDB	Drug	0.149001337	1	Isoquinolines	6	7	1131-64-2,581-88-4
10245190	Decanoylcarnitine	HMDB	Endo	0.191474622	2	Fatty Acid Esters	0	0	1492-27-9
835	Dehydroascorbic acid	HMDB	Endo	2.339880828	2	Monosaccharides	1	7	490-83-5,19455-06-2
5881	Dehydroepiandrosterone	HMDB	Endo	0.015876742	3	Steroids and Steroid Derivatives	51	18	53-43-0,105597-37-3,108673-53-6,9013-35-8
92094	Delta-Tocopherol	HMDB	Food	11.85441059	3	Prenol Lipids	0	2	119-13-1
222528	Deoxycholic acid	HMDB	Endo	0.433700966	2	Steroids and Steroid Derivatives	3	7	83-44-3,115349-14-9
6166	Deoxycorticosterone	HMDB	Endo	0.072999696	1	Steroids and Steroid Derivatives	1	13	64-85-7
13711	Deoxycytidine	HMDB	Endo	0.200007583	1	Pyrimidine Nucleosides and Analogues	43	9	951-77-9,207121-53-7,56905-41-0
53477703	Deoxypyridinoline	HMDB	Endo	0.000700021	1	Amino Acids and Derivatives	3	0	83462-55-9
13712	Deoxyuridine	HMDB	Endo	0.184095617	3	Pyrimidine Nucleosides and Analogues	0	9	951-78-0
10394	Desaminotyrosine	HMDB	Endo	0.810017036	8	Phenols and Derivatives	0	0	501-97-3

PC_CID	Chemical	Dataset	Source	Concentration (µM)	No. studies	Class	PubMed	Biosys	CAS
439577	Desmosterol	HMDB	Endo	2.6216398	2	Steroids and Steroid Derivatives	3	7	313-04-2
439163	D-Fructose	HMDB	Endo	38.57483034	2	Monosaccharides	11	6	10247-46-8
13734178	DG(14:0/16:0/0:0)	HMDB	Endo	0.580015762	1	Glycerolipids	0	0	.
53477950	DG(14:0/16:1(9Z)/0:0)	HMDB	Endo	0.231008678	1	Glycerolipids	0	0	.
53477951	DG(14:0/18:0/0:0)	HMDB	Endo	1.420061244	1	Glycerolipids	0	0	.
53477952	DG(14:0/18:1(11Z)/0:0)	HMDB	Endo	1.070044247	1	Glycerolipids	0	0	.
14275352	DG(14:0/18:2(9Z,12Z)/0:0)	HMDB	Endo	0.414990355	1	Glycerolipids	0	0	.
53477953	DG(14:0/18:3(6Z,9Z,12Z)/0:0)	HMDB	Endo	0.060000643	1	Glycerolipids	0	0	.
53477955	DG(14:0/20:0/0:0)	HMDB	Endo	1.369985286	1	Glycerolipids	0	0	.
53477956	DG(14:0/20:1(11Z)/0:0)	HMDB	Endo	3.870176062	1	Glycerolipids	0	0	.
53477957	DG(14:0/20:2(11Z,14Z)/0:0)	HMDB	Endo	2.819895986	1	Eicosanoids	0	0	.
53477958	DG(14:0/20:3(5Z,8Z,11Z)/0:0)	HMDB	Endo	1.650040775	1	Eicosanoids	0	0	.
53477960	DG(14:0/20:4(5Z,8Z,11Z,14Z)/0:0)	HMDB	Endo	0.172993718	1	Eicosanoids	0	0	.
53477963	DG(14:0/22:0/0:0)	HMDB	Endo	0.689974941	1	Glycerolipids	0	0	.
53477964	DG(14:0/22:1(13Z)/0:0)	HMDB	Endo	1.239985889	1	Glycerolipids	0	0	.
53477965	DG(14:0/22:2(13Z,16Z)/0:0)	HMDB	Endo	4.060069128	1	Glycerolipids	0	0	.
53477966	DG(14:0/22:4(7Z,10Z,13Z,16Z)/0:0)	HMDB	Endo	5.279862206	1	Glycerolipids	0	0	.
53477967	DG(14:0/22:5(4Z,7Z,10Z,13Z,16Z)/0:0)	HMDB	Endo	0.960981616	1	Glycerolipids	0	0	.
53477970	DG(14:0/24:0/0:0)	HMDB	Endo	0.003000129	1	Glycerolipids	0	0	.
53477971	DG(14:0/24:1(15Z)/0:0)	HMDB	Endo	0.013000077	1	Glycerolipids	0	0	.
53477975	DG(14:1(9Z)/16:0/0:0)	HMDB	Endo	0.231008678	1	Glycerolipids	0	0	.
53477976	DG(14:1(9Z)/16:1(9Z)/0:0)	HMDB	Endo	0.03200062	1	Glycerolipids	0	0	.
53477977	DG(14:1(9Z)/18:0/0:0)	HMDB	Endo	1.070044247	1	Glycerolipids	0	0	.
53477978	DG(14:1(9Z)/18:1(11Z)/0:0)	HMDB	Endo	0.414990355	1	Glycerolipids	0	0	.
53477980	DG(14:1(9Z)/18:2(9Z,12Z)/0:0)	HMDB	Endo	0.060000643	1	Glycerolipids	0	0	.
53477984	DG(14:1(9Z)/20:0/0:0)	HMDB	Endo	3.870176062	1	Glycerolipids	0	0	.
53477985	DG(14:1(9Z)/20:1(11Z)/0:0)	HMDB	Endo	2.819895986	1	Glycerolipids	0	0	.
53477986	DG(14:1(9Z)/20:2(11Z,14Z)/0:0)	HMDB	Endo	1.650040775	1	Eicosanoids	0	0	.
53477987	DG(14:1(9Z)/20:3(5Z,8Z,11Z)/0:0)	HMDB	Endo	0.172993718	1	Eicosanoids	0	0	.
53477992	DG(14:1(9Z)/22:0/0:0)	HMDB	Endo	1.239985889	1	Glycerolipids	0	0	.
53477993	DG(14:1(9Z)/22:1(13Z)/0:0)	HMDB	Endo	4.060069128	1	Glycerolipids	0	0	.

PC_CID	Chemical	Dataset	Source	Concentration (µM)	No. studies	Class	PubMed	Biosys	CAS
53477994	DG(14:1(9Z)/22:2(13Z,16Z)/0:0)	HMDB	Endo	8.100291106	1	Glycerolipids	0	0	.
53477995	DG(14:1(9Z)/22:4(7Z,10Z,13Z,16Z)/0:0)	HMDB	Endo	0.960981616	1	Glycerolipids	0	0	.
53477999	DG(14:1(9Z)/24:0/0:0)	HMDB	Endo	0.013000077	1	Glycerolipids	0	0	.
53478000	DG(14:1(9Z)/24:1(15Z)/0:0)	HMDB	Endo	0.022000282	1	Glycerolipids	0	0	.
18642216	DG(15:0/15:0/0:0)	HMDB	Endo	0.580015762	1	Glycerolipids	0	0	.
53478024	DG(16:0/14:0/0:0)	HMDB	Endo	0.580015762	1	Glycerolipids	0	0	.
53478025	DG(16:0/14:1(9Z)/0:0)	HMDB	Endo	0.231008678	1	Glycerolipids	0	0	.
644078	DG(16:0/16:0/0:0)	HMDB	Endo	1.420061244	1	Glycerolipids	0	0	.
9543678	DG(16:0/16:1(9Z)/0:0)	HMDB	Endo	1.070044247	1	Glycerolipids	0	4	.
9543688	DG(16:0/18:0/0:0)	HMDB	Endo	1.369985286	1	Glycerolipids	0	4	.
9543972	DG(16:0/18:1(11Z)/0:0)	HMDB	Endo	3.870176062	1	Glycerolipids	0	4	.
9543695	DG(16:0/18:2(9Z,12Z)/0:0)	HMDB	Endo	2.819895986	1	Glycerolipids	0	4	.
14275390	DG(16:0/18:3(6Z,9Z,12Z)/0:0)	HMDB	Endo	1.650040775	1	Glycerolipids	0	0	.
53478027	DG(16:0/18:4(6Z,9Z,12Z,15Z)/0:0)	HMDB	Endo	0.172993718	1	Glycerolipids	0	0	.
9543710	DG(16:0/20:0/0:0)	HMDB	Endo	0.689974941	1	Glycerolipids	0	4	.
9543715	DG(16:0/20:1(11Z)/0:0)	HMDB	Endo	1.239985889	1	Glycerolipids	0	4	.
9543721	DG(16:0/20:2(11Z,14Z)/0:0)	HMDB	Endo	4.060069128	1	Glycerolipids	0	0	.
53478028	DG(16:0/20:3(5Z,8Z,11Z)/0:0)	HMDB	Endo	8.100291106	1	Glycerolipids	0	0	.
9543736	DG(16:0/20:4(5Z,8Z,11Z,14Z)/0:0)	HMDB	Endo	5.279862206	1	Glycerolipids	0	4	.
9543744	DG(16:0/20:5(5Z,8Z,11Z,14Z,17Z)/0:0)	HMDB	Endo	0.960981616	1	Glycerolipids	0	0	.
9543762	DG(16:0/22:0/0:0)	HMDB	Endo	0.003000129	1	Glycerolipids	0	0	.
9543772	DG(16:0/22:1(13Z)/0:0)	HMDB	Endo	0.013000077	1	Glycerolipids	0	0	.
9543792	DG(16:0/22:2(13Z,16Z)/0:0)	HMDB	Endo	0.022000282	1	Glycerolipids	0	0	.
9543803	DG(16:0/22:4(7Z,10Z,13Z,16Z)/0:0)	HMDB	Endo	0.93997688	1	Glycerolipids	0	0	.
53478030	DG(16:0/22:5(4Z,7Z,10Z,13Z,16Z)/0:0)	HMDB	Endo	1.609944973	1	Glycerolipids	0	0	.
9543827	DG(16:0/22:6(4Z,7Z,10Z,13Z,16Z,19Z)/0:0)	HMDB	Endo	1.137007715	1	Glycerolipids	0	0	.
53478033	DG(16:1(9Z)/14:0/0:0)	HMDB	Endo	0.231008678	1	Glycerolipids	0	0	.
53478034	DG(16:1(9Z)/14:1(9Z)/0:0)	HMDB	Endo	0.03200062	1	Glycerolipids	0	0	.
53478036	DG(16:1(9Z)/16:0/0:0)	HMDB	Endo	1.070044247	1	Glycerolipids	0	0	.
9543679	DG(16:1(9Z)/16:1(9Z)/0:0)	HMDB	Endo	0.414990355	1	Glycerolipids	0	4	.
9543691	DG(16:1(9Z)/18:0/0:0)	HMDB	Endo	3.870176062	1	Glycerolipids	0	4	.

PC_CID	Chemical	Dataset	Source	Concentration (μM)	No. studies	Class	PubMed	Biosys	CAS
53478037	DG(16:1(9Z)/18:1(11Z)/0:0)	HMDB	Endo	2.819895986	1	Glycerolipids	0	0	.
9543699	DG(16:1(9Z)/18:2(9Z,12Z)/0:0)	HMDB	Endo	1.650040775	1	Glycerolipids	0	4	.
53478038	DG(16:1(9Z)/18:3(6Z,9Z,12Z)/0:0)	HMDB	Endo	0.172993718	1	Glycerolipids	0	0	.
9543714	DG(16:1(9Z)/20:0/0:0)	HMDB	Endo	1.239985889	1	Glycerolipids	0	4	.
9543720	DG(16:1(9Z)/20:1(11Z)/0:0)	HMDB	Endo	4.060069128	1	Glycerolipids	0	4	.
9543727	DG(16:1(9Z)/20:2(11Z,14Z)/0:0)	HMDB	Endo	8.100291106	1	Glycerolipids	0	0	.
53478040	DG(16:1(9Z)/20:3(5Z,8Z,11Z)/0:0)	HMDB	Endo	5.279862206	1	Glycerolipids	0	0	.
9543743	DG(16:1(9Z)/20:4(5Z,8Z,11Z,14Z)/0:0)	HMDB	Endo	0.960981616	1	Glycerolipids	0	4	.
9543771	DG(16:1(9Z)/22:0/0:0)	HMDB	Endo	0.013000077	1	Glycerolipids	0	0	.
9543780	DG(16:1(9Z)/22:1(13Z)/0:0)	HMDB	Endo	0.022000282	1	Glycerolipids	0	0	.
9543802	DG(16:1(9Z)/22:2(13Z,16Z)/0:0)	HMDB	Endo	0.279990389	1	Glycerolipids	0	0	.
9543814	DG(16:1(9Z)/22:4(7Z,10Z,13Z,16Z)/0:0)	HMDB	Endo	1.609944973	1	Glycerolipids	0	0	.
53478042	DG(16:1(9Z)/22:5(4Z,7Z,10Z,13Z,16Z)/0:0)	HMDB	Endo	1.137007715	1	Glycerolipids	0	0	.
53478045	DG(18:0/14:0/0:0)	HMDB	Endo	1.420061244	1	Glycerolipids	0	0	.
53478046	DG(18:0/14:1(9Z)/0:0)	HMDB	Endo	1.070044247	1	Glycerolipids	0	0	.
3246945	DG(18:0/16:0/0:0)	HMDB	Endo	1.369985286	1	Glycerolipids	0	4	.
53478048	DG(18:0/16:1(9Z)/0:0)	HMDB	Endo	3.870176062	1	Glycerolipids	0	0	.
9543705	DG(18:0/18:0/0:0)	HMDB	Endo	0.689974941	1	Glycerolipids	0	0	10567-21-2
53478049	DG(18:0/18:1(11Z)/0:0)	HMDB	Endo	1.239985889	1	Glycerolipids	0	0	.
6441562	DG(18:0/18:2(9Z,12Z)/0:0)	HMDB	Endo	4.060069128	1	Glycerolipids	0	4	34487-26-8
53478050	DG(18:0/18:3(6Z,9Z,12Z)/0:0)	HMDB	Endo	8.100291106	1	Glycerolipids	0	0	.
53478051	DG(18:0/18:4(6Z,9Z,12Z,15Z)/0:0)	HMDB	Endo	5.279862206	1	Glycerolipids	0	0	.
9543739	DG(18:0/20:0/0:0)	HMDB	Endo	0.003000129	1	Glycerolipids	0	4	.
9543748	DG(18:0/20:1(11Z)/0:0)	HMDB	Endo	0.013000077	1	Glycerolipids	0	4	.
9543757	DG(18:0/20:2(11Z,14Z)/0:0)	HMDB	Endo	0.022000282	1	Glycerolipids	0	0	.
53478052	DG(18:0/20:3(5Z,8Z,11Z)/0:0)	HMDB	Endo	0.279990389	1	Glycerolipids	0	0	.
6438587	DG(18:0/20:4(5Z,8Z,11Z,14Z)/0:0)	HMDB	Endo	0.93997688	1	Glycerolipids	0	4	.
9543787	DG(18:0/20:5(5Z,8Z,11Z,14Z,17Z)/0:0)	HMDB	Endo	1.609944973	1	Glycerolipids	0	0	.
9543859	DG(18:0/22:4(7Z,10Z,13Z,16Z)/0:0)	HMDB	Endo	0.036001308	1	Glycerolipids	0	0	.
9543881	DG(18:0/22:6(4Z,7Z,10Z,13Z,16Z,19Z)/0:0)	HMDB	Endo	0.254005337	1	Glycerolipids	0	0	.
53478057	DG(18:1(11Z)/14:0/0:0)	HMDB	Endo	1.070044247	1	Glycerolipids	0	0	.

PC_CID	Chemical	Dataset	Source	Concentration (µM)	No. studies	Class	PubMed	Biosys	CAS
53478058	DG(18:1(11Z)/14:1(9Z)/0:0)	HMDB	Endo	0.414990355	1	Glycerolipids	0	0	.
53478060	DG(18:1(11Z)/16:0/0:0)	HMDB	Endo	3.870176062	1	Glycerolipids	0	0	.
53478061	DG(18:1(11Z)/16:1(9Z)/0:0)	HMDB	Endo	2.819895986	1	Glycerolipids	0	0	.
53478062	DG(18:1(11Z)/18:0/0:0)	HMDB	Endo	1.239985889	1	Glycerolipids	0	0	.
53478063	DG(18:1(11Z)/18:1(11Z)/0:0)	HMDB	Endo	4.060069128	1	Glycerolipids	0	0	.
53478065	DG(18:1(11Z)/18:2(9Z,12Z)/0:0)	HMDB	Endo	8.100291106	1	Glycerolipids	0	0	.
53478066	DG(18:1(11Z)/18:3(6Z,9Z,12Z)/0:0)	HMDB	Endo	5.279862206	1	Glycerolipids	0	0	.
53478068	DG(18:1(11Z)/18:4(6Z,9Z,12Z,15Z)/0:0)	HMDB	Endo	0.960981616	1	Glycerolipids	0	0	.
53478069	DG(18:1(11Z)/20:0/0:0)	HMDB	Endo	0.013000077	1	Glycerolipids	0	0	.
53478070	DG(18:1(11Z)/20:1(11Z)/0:0)	HMDB	Endo	0.022000282	1	Glycerolipids	0	0	.
53478071	DG(18:1(11Z)/20:2(11Z,14Z)/0:0)	HMDB	Endo	0.279990389	1	Glycerolipids	0	0	.
53478072	DG(18:1(11Z)/20:3(5Z,8Z,11Z)/0:0)	HMDB	Endo	0.93997688	1	Glycerolipids	0	0	.
53478074	DG(18:1(11Z)/20:4(5Z,8Z,11Z,14Z)/0:0)	HMDB	Endo	1.609944973	1	Glycerolipids	0	0	.
53478076	DG(18:1(11Z)/20:5(5Z,8Z,11Z,14Z,17Z)/0:0)	HMDB	Endo	1.137007715	1	Glycerolipids	0	0	.
53478081	DG(18:1(11Z)/22:5(4Z,7Z,10Z,13Z,16Z)/0:0)	HMDB	Endo	0.254005337	1	Glycerolipids	0	0	.
53478083	DG(18:1(11Z)/22:6(4Z,7Z,10Z,13Z,16Z,19Z)/0:0)	HMDB	Endo	0.309994724	1	Glycerolipids	0	0	.
53478098	DG(18:2(9Z,12Z)/14:0/0:0)	HMDB	Endo	0.414990355	1	Glycerolipids	0	0	.
53478099	DG(18:2(9Z,12Z)/14:1(9Z)/0:0)	HMDB	Endo	0.060000643	1	Glycerolipids	0	0	.
53478101	DG(18:2(9Z,12Z)/16:0/0:0)	HMDB	Endo	2.819895986	1	Glycerolipids	0	0	.
53478102	DG(18:2(9Z,12Z)/16:1(9Z)/0:0)	HMDB	Endo	1.650040775	1	Glycerolipids	0	0	.
53478103	DG(18:2(9Z,12Z)/18:0/0:0)	HMDB	Endo	4.060069128	1	Glycerolipids	0	0	.
53478104	DG(18:2(9Z,12Z)/18:1(11Z)/0:0)	HMDB	Endo	8.100291106	1	Glycerolipids	0	0	.
9543729	DG(18:2(9Z,12Z)/18:2(9Z,12Z)/0:0)	HMDB	Endo	5.279862206	1	Glycerolipids	0	4	24529-89-3
14275409	DG(18:2(9Z,12Z)/18:3(6Z,9Z,12Z)/0:0)	HMDB	Endo	0.960981616	1	Glycerolipids	0	0	.
9543755	DG(18:2(9Z,12Z)/20:0/0:0)	HMDB	Endo	0.022000282	1	Glycerolipids	0	4	.
9543765	DG(18:2(9Z,12Z)/20:1(11Z)/0:0)	HMDB	Endo	0.279990389	1	Glycerolipids	0	4	.
9543775	DG(18:2(9Z,12Z)/20:2(11Z,14Z)/0:0)	HMDB	Endo	0.93997688	1	Glycerolipids	0	0	.
53478107	DG(18:2(9Z,12Z)/20:3(5Z,8Z,11Z)/0:0)	HMDB	Endo	1.609944973	1	Glycerolipids	0	0	.
9543796	DG(18:2(9Z,12Z)/20:4(5Z,8Z,11Z,14Z)/0:0)	HMDB	Endo	1.137007715	1	Glycerolipids	0	4	.
9543868	DG(18:2(9Z,12Z)/22:2(13Z,16Z)/0:0)	HMDB	Endo	0.036001308	1	Glycerolipids	0	0	.
9543879	DG(18:2(9Z,12Z)/22:4(7Z,10Z,13Z,16Z)/0:0)	HMDB	Endo	0.254005337	1	Glycerolipids	0	0	.

PC_CID	Chemical	Dataset	Source	Concentration (µM)	No. studies	Class	PubMed	Biosys	CAS
53478109	DG(18:2(9Z,12Z)/22:5(4Z,7Z,10Z,13Z,16Z)/0:0)	HMDB	Endo	0.309994724	1	Glycerolipids	0	0	.
53478112	DG(18:3(6Z,9Z,12Z)/14:0/0:0)	HMDB	Endo	0.060000643	1	Glycerolipids	0	0	.
53478115	DG(18:3(6Z,9Z,12Z)/16:0/0:0)	HMDB	Endo	1.650040775	1	Glycerolipids	0	0	.
53478116	DG(18:3(6Z,9Z,12Z)/16:1(9Z)/0:0)	HMDB	Endo	0.172993718	1	Glycerolipids	0	0	.
53478117	DG(18:3(6Z,9Z,12Z)/18:0/0:0)	HMDB	Endo	8.100291106	1	Glycerolipids	0	0	.
53478118	DG(18:3(6Z,9Z,12Z)/18:1(11Z)/0:0)	HMDB	Endo	5.279862206	1	Glycerolipids	0	0	.
53478120	DG(18:3(6Z,9Z,12Z)/18:2(9Z,12Z)/0:0)	HMDB	Endo	0.960981616	1	Glycerolipids	0	0	.
53478123	DG(18:3(6Z,9Z,12Z)/20:0/0:0)	HMDB	Endo	0.279990389	1	Glycerolipids	0	0	.
53478124	DG(18:3(6Z,9Z,12Z)/20:1(11Z)/0:0)	HMDB	Endo	0.93997688	1	Glycerolipids	0	0	.
53478125	DG(18:3(6Z,9Z,12Z)/20:2(11Z,14Z)/0:0)	HMDB	Endo	1.609944973	1	Glycerolipids	0	0	.
53478126	DG(18:3(6Z,9Z,12Z)/20:3(5Z,8Z,11Z)/0:0)	HMDB	Endo	1.137007715	1	Glycerolipids	0	0	.
53478132	DG(18:3(6Z,9Z,12Z)/22:1(13Z)/0:0)	HMDB	Endo	0.036001308	1	Glycerolipids	0	0	.
53478134	DG(18:3(6Z,9Z,12Z)/22:4(7Z,10Z,13Z,16Z)/0:0)	HMDB	Endo	0.309994724	1	Glycerolipids	0	0	.
53478140	DG(18:3(9Z,12Z,15Z)/14:0/0:0)	HMDB	Endo	0.060000643	1	Glycerolipids	0	0	.
53478159	DG(18:4(6Z,9Z,12Z,15Z)/16:0/0:0)	HMDB	Endo	0.172993718	1	Glycerolipids	0	0	.
53478161	DG(18:4(6Z,9Z,12Z,15Z)/18:0/0:0)	HMDB	Endo	5.279862206	1	Glycerolipids	0	0	.
53478162	DG(18:4(6Z,9Z,12Z,15Z)/18:1(11Z)/0:0)	HMDB	Endo	0.960981616	1	Glycerolipids	0	0	.
53478168	DG(18:4(6Z,9Z,12Z,15Z)/20:0/0:0)	HMDB	Endo	0.93997688	1	Glycerolipids	0	0	.
53478169	DG(18:4(6Z,9Z,12Z,15Z)/20:1(11Z)/0:0)	HMDB	Endo	1.609944973	1	Glycerolipids	0	0	.
53478170	DG(18:4(6Z,9Z,12Z,15Z)/20:2(11Z,14Z)/0:0)	HMDB	Endo	1.137007715	1	Glycerolipids	0	0	.
53478176	DG(18:4(6Z,9Z,12Z,15Z)/22:0/0:0)	HMDB	Endo	0.036001308	1	Glycerolipids	0	0	.
53478178	DG(18:4(6Z,9Z,12Z,15Z)/22:2(13Z,16Z)/0:0)	HMDB	Endo	0.254005337	1	Glycerolipids	0	0	.
53478185	DG(20:0/14:0/0:0)	HMDB	Endo	1.369985286	1	Glycerolipids	0	0	.
53478186	DG(20:0/14:1(9Z)/0:0)	HMDB	Endo	3.870176062	1	Glycerolipids	0	0	.
53478188	DG(20:0/16:0/0:0)	HMDB	Endo	0.689974941	1	Glycerolipids	0	0	.
53478189	DG(20:0/16:1(9Z)/0:0)	HMDB	Endo	1.239985889	1	Glycerolipids	0	0	.
3246949	DG(20:0/18:0/0:0)	HMDB	Endo	0.003000129	1	Glycerolipids	0	0	.
53478190	DG(20:0/18:1(11Z)/0:0)	HMDB	Endo	0.013000077	1	Glycerolipids	0	0	.
53478192	DG(20:0/18:2(9Z,12Z)/0:0)	HMDB	Endo	0.022000282	1	Glycerolipids	0	0	.
53478193	DG(20:0/18:3(6Z,9Z,12Z)/0:0)	HMDB	Endo	0.279990389	1	Glycerolipids	0	0	.
53478195	DG(20:0/18:4(6Z,9Z,12Z,15Z)/0:0)	HMDB	Endo	0.93997688	1	Glycerolipids	0	0	.

PC_CID	Chemical	Dataset	Source	Concentration (µM)	No. studies	Class	PubMed	Biosys	CAS
9543830	DG(20:0/20:4(5Z,8Z,11Z,14Z)/0:0)	HMDB	Endo	0.036001308	1	Glycerolipids	0	4	.
53478201	DG(20:1(11Z)/14:0/0:0)	HMDB	Endo	3.870176062	1	Glycerolipids	0	0	.
53478202	DG(20:1(11Z)/14:1(9Z)/0:0)	HMDB	Endo	2.819895986	1	Glycerolipids	0	0	.
53478204	DG(20:1(11Z)/16:0/0:0)	HMDB	Endo	1.239985889	1	Glycerolipids	0	0	.
53478205	DG(20:1(11Z)/16:1(9Z)/0:0)	HMDB	Endo	4.060069128	1	Glycerolipids	0	0	.
53478206	DG(20:1(11Z)/18:0/0:0)	HMDB	Endo	0.013000077	1	Glycerolipids	0	0	.
53478207	DG(20:1(11Z)/18:1(11Z)/0:0)	HMDB	Endo	0.022000282	1	Glycerolipids	0	0	.
53478209	DG(20:1(11Z)/18:2(9Z,12Z)/0:0)	HMDB	Endo	0.279990389	1	Glycerolipids	0	0	.
53478210	DG(20:1(11Z)/18:3(6Z,9Z,12Z)/0:0)	HMDB	Endo	0.93997688	1	Glycerolipids	0	0	.
53478212	DG(20:1(11Z)/18:4(6Z,9Z,12Z,15Z)/0:0)	HMDB	Endo	1.609944973	1	Glycerolipids	0	0	.
53478214	DG(20:1(11Z)/20:3(5Z,8Z,11Z)/0:0)	HMDB	Endo	0.036001308	1	Glycerolipids	0	0	.
9543853	DG(20:1(11Z)/20:5(5Z,8Z,11Z,14Z,17Z)/0:0)	HMDB	Endo	0.254005337	1	Glycerolipids	0	0	.
53478219	DG(20:2(11Z,14Z)/14:0/0:0)	HMDB	Endo	2.819895986	1	Glycerolipids	0	0	.
53478220	DG(20:2(11Z,14Z)/14:1(9Z)/0:0)	HMDB	Endo	1.650040775	1	Glycerolipids	0	0	.
53478222	DG(20:2(11Z,14Z)/16:0/0:0)	HMDB	Endo	4.060069128	1	Glycerolipids	0	0	.
53478223	DG(20:2(11Z,14Z)/16:1(9Z)/0:0)	HMDB	Endo	8.100291106	1	Glycerolipids	0	0	.
53478224	DG(20:2(11Z,14Z)/18:0/0:0)	HMDB	Endo	0.022000282	1	Glycerolipids	0	0	.
53478225	DG(20:2(11Z,14Z)/18:1(11Z)/0:0)	HMDB	Endo	0.279990389	1	Glycerolipids	0	0	.
53478227	DG(20:2(11Z,14Z)/18:2(9Z,12Z)/0:0)	HMDB	Endo	0.93997688	1	Glycerolipids	0	0	.
53478228	DG(20:2(11Z,14Z)/18:3(6Z,9Z,12Z)/0:0)	HMDB	Endo	1.609944973	1	Glycerolipids	0	0	.
53478230	DG(20:2(11Z,14Z)/18:4(6Z,9Z,12Z,15Z)/0:0)	HMDB	Endo	1.137007715	1	Glycerolipids	0	0	.
9543828	DG(20:2(11Z,14Z)/20:2(11Z,14Z)/0:0)	HMDB	Endo	0.036001308	1	Glycerolipids	0	0	.
9543852	DG(20:2(11Z,14Z)/20:4(5Z,8Z,11Z,14Z)/0:0)	HMDB	Endo	0.254005337	1	Glycerolipids	0	0	.
9543863	DG(20:2(11Z,14Z)/20:5(5Z,8Z,11Z,14Z,17Z)/0:0)	HMDB	Endo	0.309994724	1	Glycerolipids	0	0	.
53478238	DG(20:3(5Z,8Z,11Z)/14:0/0:0)	HMDB	Endo	1.650040775	1	Glycerolipids	0	0	.
53478239	DG(20:3(5Z,8Z,11Z)/14:1(9Z)/0:0)	HMDB	Endo	0.172993718	1	Glycerolipids	0	0	.
53478241	DG(20:3(5Z,8Z,11Z)/16:0/0:0)	HMDB	Endo	8.100291106	1	Glycerolipids	0	0	.
53478242	DG(20:3(5Z,8Z,11Z)/16:1(9Z)/0:0)	HMDB	Endo	5.279862206	1	Glycerolipids	0	0	.
53478243	DG(20:3(5Z,8Z,11Z)/18:0/0:0)	HMDB	Endo	0.279990389	1	Glycerolipids	0	0	.
53478244	DG(20:3(5Z,8Z,11Z)/18:1(11Z)/0:0)	HMDB	Endo	0.93997688	1	Glycerolipids	0	0	.
53478246	DG(20:3(5Z,8Z,11Z)/18:2(9Z,12Z)/0:0)	HMDB	Endo	1.609944973	1	Glycerolipids	0	0	.

PC_CID	Chemical	Dataset	Source	Concentration (µM)	No. studies	Class	PubMed	Biosys	CAS
53478247	DG(20:3(5Z,8Z,11Z)/18:3(6Z,9Z,12Z)/0:0)	HMDB	Endo	1.137007715	1	Glycerolipids	0	0	.
53478251	DG(20:3(5Z,8Z,11Z)/20:1(11Z)/0:0)	HMDB	Endo	0.036001308	1	Glycerolipids	0	0	.
53478253	DG(20:3(5Z,8Z,11Z)/20:3(5Z,8Z,11Z)/0:0)	HMDB	Endo	0.254005337	1	Glycerolipids	0	0	.
53478255	DG(20:3(5Z,8Z,11Z)/20:4(5Z,8Z,11Z,14Z)/0:0)	HMDB	Endo	0.309994724	1	Glycerolipids	0	0	.
53478280	DG(20:3(8Z,11Z,14Z)/20:1(11Z)/0:0)	HMDB	Endo	0.036001308	1	Glycerolipids	0	0	.
53478287	DG(20:4(5Z,8Z,11Z,14Z)/14:0/0:0)	HMDB	Endo	0.172993718	1	Glycerolipids	0	0	.
53478290	DG(20:4(5Z,8Z,11Z,14Z)/16:0/0:0)	HMDB	Endo	5.279862206	1	Glycerolipids	0	0	.
53478291	DG(20:4(5Z,8Z,11Z,14Z)/16:1(9Z)/0:0)	HMDB	Endo	0.960981616	1	Glycerolipids	0	0	.
53478292	DG(20:4(5Z,8Z,11Z,14Z)/18:0/0:0)	HMDB	Endo	0.93997688	1	Glycerolipids	0	0	.
53478293	DG(20:4(5Z,8Z,11Z,14Z)/18:1(11Z)/0:0)	HMDB	Endo	1.609944973	1	Glycerolipids	0	0	.
53478295	DG(20:4(5Z,8Z,11Z,14Z)/18:2(9Z,12Z)/0:0)	HMDB	Endo	1.137007715	1	Glycerolipids	0	0	.
53478299	DG(20:4(5Z,8Z,11Z,14Z)/20:0/0:0)	HMDB	Endo	0.036001308	1	Glycerolipids	0	0	.
53478301	DG(20:4(5Z,8Z,11Z,14Z)/20:2(11Z,14Z)/0:0)	HMDB	Endo	0.254005337	1	Glycerolipids	0	0	.
53478302	DG(20:4(5Z,8Z,11Z,14Z)/20:3(5Z,8Z,11Z)/0:0)	HMDB	Endo	0.309994724	1	Glycerolipids	0	0	.
53478340	DG(20:5(5Z,8Z,11Z,14Z,17Z)/16:0/0:0)	HMDB	Endo	0.960981616	1	Glycerolipids	0	0	.
53478342	DG(20:5(5Z,8Z,11Z,14Z,17Z)/18:0/0:0)	HMDB	Endo	1.609944973	1	Glycerolipids	0	0	.
53478343	DG(20:5(5Z,8Z,11Z,14Z,17Z)/18:1(11Z)/0:0)	HMDB	Endo	1.137007715	1	Glycerolipids	0	0	.
53478350	DG(20:5(5Z,8Z,11Z,14Z,17Z)/20:1(11Z)/0:0)	HMDB	Endo	0.254005337	1	Glycerolipids	0	0	.
53478351	DG(20:5(5Z,8Z,11Z,14Z,17Z)/20:2(11Z,14Z)/0:0)	HMDB	Endo	0.309994724	1	Glycerolipids	0	0	.
53478359	DG(22:0/14:0/0:0)	HMDB	Endo	0.689974941	1	Glycerolipids	0	0	.
53478360	DG(22:0/14:1(9Z)/0:0)	HMDB	Endo	1.239985889	1	Glycerolipids	0	0	.
53478362	DG(22:0/16:0/0:0)	HMDB	Endo	0.003000129	1	Glycerolipids	0	0	.
53478363	DG(22:0/16:1(9Z)/0:0)	HMDB	Endo	0.013000077	1	Glycerolipids	0	0	.
53478370	DG(22:0/18:4(6Z,9Z,12Z,15Z)/0:0)	HMDB	Endo	0.036001308	1	Glycerolipids	0	0	.
53478382	DG(22:1(13Z)/14:0/0:0)	HMDB	Endo	1.239985889	1	Glycerolipids	0	0	.
53478383	DG(22:1(13Z)/14:1(9Z)/0:0)	HMDB	Endo	4.060069128	1	Glycerolipids	0	0	.
53478385	DG(22:1(13Z)/16:0/0:0)	HMDB	Endo	0.013000077	1	Glycerolipids	0	0	.
53478386	DG(22:1(13Z)/16:1(9Z)/0:0)	HMDB	Endo	0.022000282	1	Glycerolipids	0	0	.
53478391	DG(22:1(13Z)/18:3(6Z,9Z,12Z)/0:0)	HMDB	Endo	0.036001308	1	Glycerolipids	0	0	.
53478406	DG(22:2(13Z,16Z)/14:0/0:0)	HMDB	Endo	4.060069128	1	Glycerolipids	0	0	.
53478407	DG(22:2(13Z,16Z)/14:1(9Z)/0:0)	HMDB	Endo	8.100291106	1	Glycerolipids	0	0	.

PC_CID	Chemical	Dataset	Source	Concentration (µM)	No. studies	Class	PubMed	Biosys	CAS
53478409	DG(22:2(13Z,16Z)/16:0/0:0)	HMDB	Endo	0.022000282	1	Glycerolipids	0	0	.
53478410	DG(22:2(13Z,16Z)/16:1(9Z)/0:0)	HMDB	Endo	0.279990389	1	Glycerolipids	0	0	.
53478414	DG(22:2(13Z,16Z)/18:2(9Z,12Z)/0:0)	HMDB	Endo	0.036001308	1	Glycerolipids	0	0	.
53478417	DG(22:2(13Z,16Z)/18:4(6Z,9Z,12Z,15Z)/0:0)	HMDB	Endo	0.254005337	1	Glycerolipids	0	0	.
53478431	DG(22:4(7Z,10Z,13Z,16Z)/14:0/0:0)	HMDB	Endo	5.279862206	1	Glycerolipids	0	0	.
53478432	DG(22:4(7Z,10Z,13Z,16Z)/14:1(9Z)/0:0)	HMDB	Endo	0.960981616	1	Glycerolipids	0	0	.
53478434	DG(22:4(7Z,10Z,13Z,16Z)/16:0/0:0)	HMDB	Endo	0.93997688	1	Glycerolipids	0	0	.
53478435	DG(22:4(7Z,10Z,13Z,16Z)/16:1(9Z)/0:0)	HMDB	Endo	1.609944973	1	Glycerolipids	0	0	.
53478436	DG(22:4(7Z,10Z,13Z,16Z)/18:0/0:0)	HMDB	Endo	0.036001308	1	Glycerolipids	0	0	.
53478439	DG(22:4(7Z,10Z,13Z,16Z)/18:2(9Z,12Z)/0:0)	HMDB	Endo	0.254005337	1	Glycerolipids	0	0	.
53478440	DG(22:4(7Z,10Z,13Z,16Z)/18:3(6Z,9Z,12Z)/0:0)	HMDB	Endo	0.309994724	1	Glycerolipids	0	0	.
53478457	DG(22:5(4Z,7Z,10Z,13Z,16Z)/14:0/0:0)	HMDB	Endo	0.960981616	1	Glycerolipids	0	0	.
53478460	DG(22:5(4Z,7Z,10Z,13Z,16Z)/16:0/0:0)	HMDB	Endo	1.609944973	1	Glycerolipids	0	0	.
53478461	DG(22:5(4Z,7Z,10Z,13Z,16Z)/16:1(9Z)/0:0)	HMDB	Endo	1.137007715	1	Glycerolipids	0	0	.
53478463	DG(22:5(4Z,7Z,10Z,13Z,16Z)/18:1(11Z)/0:0)	HMDB	Endo	0.254005337	1	Glycerolipids	0	0	.
53478465	DG(22:5(4Z,7Z,10Z,13Z,16Z)/18:2(9Z,12Z)/0:0)	HMDB	Endo	0.309994724	1	Glycerolipids	0	0	.
53478516	DG(22:6(4Z,7Z,10Z,13Z,16Z,19Z)/16:0/0:0)	HMDB	Endo	1.137007715	1	Glycerolipids	0	0	.
53478518	DG(22:6(4Z,7Z,10Z,13Z,16Z,19Z)/18:0/0:0)	HMDB	Endo	0.254005337	1	Glycerolipids	0	0	.
53478519	DG(22:6(4Z,7Z,10Z,13Z,16Z,19Z)/18:1(11Z)/0:0)	HMDB	Endo	0.309994724	1	Glycerolipids	0	0	.
53478541	DG(24:0/14:0/0:0)	HMDB	Endo	0.003000129	1	Glycerolipids	0	0	.
53478542	DG(24:0/14:1(9Z)/0:0)	HMDB	Endo	0.013000077	1	Glycerolipids	0	0	.
53478570	DG(24:1(15Z)/14:0/0:0)	HMDB	Endo	0.013000077	1	Glycerolipids	0	0	.
53478571	DG(24:1(15Z)/14:1(9Z)/0:0)	HMDB	Endo	0.022000282	1	Glycerolipids	0	0	.
439357	D-Galactose	HMDB	Endo	58.68592972	2	Monosaccharides	0	25	3646-73-9
79025	D-Glucose	HMDB	Endo	4801.098381	11	Monosaccharides	247	45	50-99-7
94715	D-Glucuronic acid	HMDB	Endo	164.992497	1	Sugar Acids and Derivatives	0	6	6556-12-3
24794350	D-Glyceraldehyde 3-phosphate	HMDB	Endo	4.799923595	1	Organic Phosphoric Acids and Derivatives	0	6	142-10-9
12594	DHEA sulfate	HMDB	Endo	3.951518755	2	Steroids and Steroid Derivatives	110	16	651-48-9,1099-87-2,1095-56-3
31296	Dibromochloromethane	NHANES	Pollut	4.16248E-06	1	Organochlorides	0	0	124-48-1
2724385	Digoxin	HMDB	Drug	0.000700021	1	Steroids and Steroid Derivatives	43	5	20830-75-5

PC_CID	Chemical	Dataset	Source	Concentration (µM)	No. studies	Class	PubMed	Biosys	CAS
5282272	Dihomo-gamma-Linolenoyl ethanolamide	HMDB	Endo	1.010050167	1	Fatty Amides	0	0	150314-34-4
15818	Dihydroandrosterone	HMDB	Endo	0.000474998	1	Steroids and Steroid Derivatives	0	0	1852-53-5
5460203	Dihydrobiopterin	HMDB	Endo	0.005999975	1	Pteridines and Derivatives	2	5	6779-87-9
98792	Dihydrofolic acid	HMDB	Endo	0.005000087	1	Pteridines and Derivatives	0	12	4033-27-6
10635	Dihydrotestosterone	HMDB	Endo	0.001487884	2	Steroids and Steroid Derivatives	19	11	521-18-6,12040-51-6,28801-96-9
649	Dihydrouracil	HMDB	Endo	0.314963034	2	Diazines	0	16	504-07-4
668	Dihydroxyacetone phosphate	HMDB	Endo	15.60045374	1	Organic Phosphoric Acids and Derivatives	0	34	57-04-5,2134-85-2
6213	Dimethyl sulfone	HMDB	Endo	8.800424861	1	Sulfonyls	0	1	67-71-0
674	Dimethylamine	HMDB	Endo	4.566741816	3	Alkylamines	1	1	124-40-3
673	Dimethylglycine	HMDB	Endo	2.222649605	2	Amino Acids and Derivatives	1	5	1118-68-9,18319-88-5,780741-89-1
123831	Dimethyl-L-arginine	HMDB	Endo	0.480609591	2	Amino Acids and Derivatives	0	0	102783-24-4
61503	D-Lactic acid	HMDB	Endo	11.000052	1	Hydroxy Acids and Derivatives	0	1	10326-41-7
10010	DL-Homocystine	HMDB	Endo	9.800172622	1	Amino Acids and Derivatives	36	1	870-93-9,462-10-2,626-72-2
440917	D-Limonene	HMDB	Food	0.190005929	1	Prenol Lipids	0	1	5989-27-5
18950	D-Mannose	HMDB	Endo	49.95886666	2	Monosaccharides	0	25	31103-86-3,530-26-7,50986-23-7,50986-24-8,147-74-0,40866-07-7
445580	Docosahexaenoic acid	NHANES	Food	124.9982828	1	Fatty Acids and Conjugates	79	14	6217-54-5,1024594-51-1,25377-50-8
5497182	Docosapentaenoic acid	NHANES	Food	41.59999304	1	Fatty Acids and Conjugates	0	10	24880-45-3
6441454	Docosapentaenoic-6 acid (22:5n-6)	NHANES	Food	19.59942051	1	Fatty Acids and Conjugates	0	4	25182-74-5
5312557	Docosatrienoic acid	HMDB	Endo	0.003999844	1	Fatty Acids and Conjugates	0	0	28845-86-5
5283028	Dodecanedioic acid	HMDB	Endo	219.7081336	1	Carboxylic Acids and Derivatives	0	1	6402-36-4
102182	Dodecanoic acid	HMDB	Food	3.277562481	4	Fatty Acids and Conjugates	0	1	1908-11-8
168381	Dodecanoylcarnitine	HMDB	Food	0.07211451	2	Fatty Acid Esters	0	0	25518-54-1
6433320	Dolichol-20	HMDB	Endo	0.03099901	1	Prenol Lipids	0	0	2067-66-5
681	Dopamine	HMDB	Endo	22.49965555	1	Phenols and Derivatives	0	54	51-61-6,50444-17-2
122136	Dopamine 3-O-sulfate	HMDB	Endo	0.026500279	1	Phenols and Derivatives	0	4	51317-41-0
123932	Dopamine 4-sulfate	HMDB	Endo	0.002700009	1	Phenols and Derivatives	0	0	38339-02-5
71082	D-Ornithine	HMDB	Endo	88.99676315	1	Amino Acids and Derivatives	3	2	348-66-3

PC_CID	Chemical	Dataset	Source	Concentration (µM)	No. studies	Class	PubMed	Biosys	CAS
736316	D-Pipecolic acid	HMDB	Endo	0.10999724	1	Amino Acids and Derivatives	0	0	1723-00-8
5779	D-Ribose	HMDB	Endo	2.299979017	1	Monosaccharides	0	3	50-69-1
77982	D-Ribose 5-phosphate	HMDB	Endo	13.19977785	1	Monosaccharides	0	9	3615-55-2
439184	D-Ribulose 5-phosphate	HMDB	Endo	1.579960742	1	Monosaccharides	0	13	4151-19-3
165007	D-Sedoheptulose 7-phosphate	HMDB	Endo	0.890030097	1	Disaccharides	0	11	2646-35-7
71077	D-Serine	HMDB	Endo	2.865090678	2	Amino Acids and Derivatives	0	5	312-84-5
145729	dUDP	HMDB	Endo	0.500023591	1	Pyrimidine Nucleotides	0	5	4208-67-7
6912	D-Xylitol	HMDB	Food	0.676989172	1	Sugar Alcohols	0	5	488-81-3
644160	D-Xylose	HMDB	Endo	2356.187204	2	Monosaccharides	0	18	25990-60-7
443844	Ecgonine methyl ester	HMDB	Drug	0.236998848	1	Piperidines	0	1	7143-09-1
6439848	Eicosadienoic acid	NHANES	Endo	21.19997495	1	Fatty Acids and Conjugates	0	1	2091-39-6
446284	Eicosapentaenoic acid	HMDB	Food	10.44997933	8	Fatty Acids and Conjugates	83	27	10417-94-4,25377-48-4,1553-41-9
5282768	Eicosenoic acid	NHANES	Food	13.60041082	1	Fatty Acids and Conjugates	0	1	26764-41-0
637517	Elaidic acid	HMDB	Food	100.0029814	1	Fatty Acids and Conjugates	2	9	112-79-8,2027-47-6,1120-45-2,861204-23-1
5281855	Ellagic acid	HMDB	Food	0.066997498	1	Benzopyrans	0	0	476-66-4
461776	Enkephalin L	HMDB	Endo	0.000300008	1	Peptides	0	0	14-18-6
11055399	Epiandrosterone	HMDB	Endo	2.899968863	1	Steroids and Steroid Derivatives	0	0	481-29-8
182232	Epicatechin	HMDB	Food	0.625002268	1	Flavonoids	0	0	35323-91-2
247732	Epietiocholanolone	HMDB	Drug	1.7E-05	1	Steroids and Steroid Derivatives	0	0	571-31-3
5816	Epinephrine	HMDB	Endo	0.000566123	4	Phenols and Derivatives	95	40	51-43-4,51028-73-0
3035453	Epinephrine sulfate	HMDB	Endo	0.005699938	1	Phenols and Derivatives	0	0	77469-50-2
228491	Epipregnanolone	HMDB	Endo	0.001009904	2	Steroids and Steroid Derivatives	0	0	128-21-2
91469	Equol	HMDB	Food	0.000599989	1	Flavonoids	10	0	531-95-3
5280793	Ergocalciferol	HMDB	Food	0.002738623	2	Steroids and Steroid Derivatives	0	2	50-14-6
444679	Ergosterol	HMDB	Food	0.009326458	1	Steroids and Steroid Derivatives	0	2	57-87-4
5281116	Erucic acid	NHANES	Food	3.44009814	1	Fatty Acids and Conjugates	0	1	112-86-7
222285	Erythritol	HMDB	Food	4.100053408	1	Sugar Alcohols	0	1	149-32-6
2781043	Erythronic acid	HMDB	Endo	1.999905641	1	Monosaccharides	0	0	13752-84-6
5757	Estradiol	HMDB	Endo	0.000116188	2	Steroids and Steroid Derivatives	373	21	50-28-2,73459-61-7,873662-39-6

PC_CID	Chemical	Dataset	Source	Concentration (µM)	No. studies	Class	PubMed	Biosys	CAS
5756	Estriol	HMDB	Endo	0.005999975	1	Steroids and Steroid Derivatives	13	2	50-27-1
160638	Estriol-3-glucuronide	HMDB	Endo	0.013999971	1	Steroids and Steroid Derivatives	0	0	2479-91-6
5870	Estrone	HMDB	Endo	0.000159798	3	Steroids and Steroid Derivatives	69	18	53-16-7,37242-41-4
3001028	Estrone sulfate	HMDB	Endo	0.001798684	6	Steroids and Steroid Derivatives	10	13	481-97-0,438-67-5
702	Ethanol	HMDB	Food	24.49575899	2	Alcohols and Polyols	95	13	64-17-5,121182-78-3,68475-56-9,71076-86-3,71329-38-9,8000-16-6,8024-45-1
700	Ethanolamine	HMDB	Endo	1.599994193	1	Alkylamines	0	12	141-43-5
7500	Ethyl benzene	NHANES	Pollut	0.000335563	3	Aromatic Homomonocyclic Compounds	0	1	100-41-4
18392195	Ethyl glucuronide	HMDB	Food	2.899968863	1	Sugar Acids and Derivatives	0	0	17685-04-0
134494	Etiocholanediol	HMDB	Drug	6.70012E-05	1	Steroids and Steroid Derivatives	0	0	1851-23-6
5880	Etiocholanolone	HMDB	Endo	0.001249015	2	Steroids and Steroid Derivatives	1	1	53-42-9
3314	Eugenol	HMDB	Food	6.49998585	1	Phenols and Derivatives	0	0	97-53-0
643975	FAD	HMDB	Endo	0.067637007	2	Flavin Nucleotides	0	93	146-14-5,16426-55-4,887565-61-9
3325	Famotidine	HMDB	Drug	4.000022556	1	Organic Sulfuric Acids and Derivatives	2	2	76824-35-6
445070	Farnesol	HMDB	Endo	0.24999859	1	Prenol Lipids	1	1	4602-84-0,106-28-5
445713	Farnesyl pyrophosphate	HMDB	Endo	0.00859994	1	Prenol Lipids	0	9	13058-04-3
643976	Flavin Mononucleotide	HMDB	Endo	0.007937158	2	Pteridines and Derivatives	1	35	146-17-8
6037	Folic acid	NHANES	Food	0.02786455	1	Pteridines and Derivatives	595	13	59-30-3,33609-88-0,47707-78-8
284	Formic acid	HMDB	Endo	98.83976465	4	Carboxylic Acids and Derivatives	0	70	64-18-6,82069-14-5,15907-03-6,8006-93-7
20484	Fructosamine	HMDB	Endo	1427.528634	4	Monosaccharides	44	2	4429-04-3
10267	Fructose 1,6-bisphosphate	HMDB	Endo	1.199974132	1	Monosaccharides	0	8	488-69-7
69507	Fructose 6-phosphate	HMDB	Endo	10.20012525	1	Monosaccharides	0	25	6814-87-5
444972	Fumaric acid	HMDB	Endo	1.500052339	1	Fatty Acids and Conjugates	0	20	110-17-8,623158-97-4,6915-18-0
3446	Gabapentin	HMDB	Drug	0.309994724	1	Amino Acids and Derivatives	0	0	60142-96-3
11850	Galactitol	HMDB	Food	0.543296537	2	Fatty Alcohols	0	2	608-66-2,18089-21-9,362631-40-1,40742-76-5
123986	Galactosylhydroxylysine	HMDB	Endo	0.060998866	1	Glycosyl Compounds	0	0	32448-36-5

PC_CID	Chemical	Dataset	Source	Concentration (μM)	No. studies	Class	PubMed	Biosys	CAS
370	Gallic acid	HMDB	Food	0.008773671	8	Benzoic Acid and Derivatives	0	0	149-91-7
119	Gamma-Aminobutyric acid	HMDB	Endo	0.10999724	1	Amino Acids and Derivatives	2	49	56-12-2,3131-86-0
7302	Gamma-Butyrolactone	HMDB	Endo	0.279990389	1	Lactones	28	0	96-48-0,187997-16-6
15887183	Gamma-CEHC	HMDB	Food	0.159997034	1	Benzopyrans	0	0	178167-77-6
123938	Gamma-Glutamylcysteine	HMDB	Endo	6.892266597	2	Amino Acids and Derivatives	1	11	636-58-8
5280933	Gamma-Linolenic acid	NHANES	Food	46.89917103	1	Lineolic Acids and Derivatives	2	12	506-26-3
92729	Gamma-Tocopherol	NHANES	Food	4.364920604	1	Prenol Lipids	0	2	54-28-4
20057285	Ganglioside GM3 (d18:1/16:0)	HMDB	Endo	4.500101716	1	Sphingolipids	0	0	54827-14-4
20057290	Ganglioside GM3 (d18:1/26:1(17Z)))	HMDB	Endo	0.566997718	1	Sphingolipids	0	0	54827-14-4
5280961	Genistein	HMDB	Food	0.003873875	4	Flavonoids	14	0	446-72-0,690224-00-1
3469	Gentisic acid	HMDB	Endo	0.806218888	2	Benzoic Acid and Derivatives	0	2	490-79-9
27812	Gestrinone	HMDB	Drug	0.24999859	1	Steroids and Steroid Derivatives	0	0	16320-04-0,40542-65-2
439213	Glucosamine	HMDB	Drug	0.289992563	1	Monosaccharides	0	1	3416-24-8
5958	Glucose 6-phosphate	HMDB	Endo	29.09888915	1	Monosaccharides	0	11	56-73-5
6475228	Glucosylceramide	HMDB	Endo	6.00024319	1	Sphingolipids	0	1	85305-87-9
20057354	Glucosylceramide (d18:1/12:0)	HMDB	Endo	3.599878158	1	Sphingolipids	0	0	85305-87-9
53481620	Glutaconylcarnitine	HMDB	Endo	0.02000046	1	Fatty Acid Esters	0	0	.
743	Glutaric acid	HMDB	Endo	0.800034842	1	Carboxylic Acids and Derivatives	0	2	110-94-1
124886	Glutathione	HMDB	Endo	21.77146135	2	Peptides	60	39	70-18-8
751	Glyceraldehyde	HMDB	Endo	1476.013276	1	Alcohols and Polyols	0	1	56-82-6
439194	Glyceric acid	HMDB	Endo	10.00014907	1	Sugar Acids and Derivatives	0	6	473-81-4
753	Glycerol	HMDB	Endo	71.87295166	5	Sugar Alcohols	9	34	56-81-5,25618-55-7,175385-78-1,280575-69-1,29796-42-7,30049-52-6,37228-54-9,64333-26-2,75398-78-6,78630-16-7,8013-25-0,8043-29-6
439162	Glycerol 3-phosphate	HMDB	Endo	30.00007855	1	Monosaccharides	0	17	17989-41-2
439285	Glycerophosphocholine	HMDB	Endo	32.99975047	1	Glycerophospholipids	0	8	28319-77-9,103709-68-8,117829-79-5
5257127	Glycine	HMDB	Endo	262.5390939	6	Amino Acids and Derivatives	30	25	56-40-6
5317750	Glycitein	HMDB	Food	0.000474333	2	Flavonoids	0	0	40957-83-3
10140	Glycocholic acid	HMDB	Endo	0.060000643	1	Steroids and Steroid Derivatives	0	16	475-31-0

PC_CID	Chemical	Dataset	Source	Concentration (µM)	No. studies	Class	PubMed	Biosys	CAS
439177	Glycogen	HMDB	Endo	41.14490071	2	Tetrasaccharides	0	1	9005-79-2
757	Glycolic acid	HMDB	Endo	7.849893776	2	Hydroxy Acids and Derivatives	0	6	79-14-1
12310288	Glycoursodeoxycholic acid	HMDB	Endo	0.190005929	1	Steroids and Steroid Derivatives	0	0	64480-66-6
10114	Glycyrrhetinic acid	HMDB	Food	0.103996296	1	Prenol Lipids	1	0	471-53-4,1449-05-4,107420-91-7,15301-63-0,202522-39-2,299198-00-8,8055-71-8
760	Glyoxylic acid	HMDB	Endo	3.049873151	1	Carboxylic Acids and Derivatives	0	10	298-12-4
23985	Gold	HMDB	Pollut	0.000239993	1	Homogeneous Transition Metal Compounds	6	0	7440-57-5
460	Guaiacol	HMDB	Endo	8.500287615	1	Phenols and Derivatives	0	0	90-05-1
3520	Guanidine	HMDB	Endo	0.17662967	2	Guanidines	0	5	50-01-1
439918	Guanidinosuccinic acid	HMDB	Endo	0.293875227	4	Amino Acids and Derivatives	0	0	6133-30-8
763	Guanidoacetic acid	HMDB	Endo	4.343584809	6	Amino Acids and Derivatives	2	8	352-97-6
6802	Guanosine	HMDB	Endo	0.800034842	1	Purine Nucleosides and Analogues	3	15	118-00-3,484-80-0,685891-87-6
8977	Guanosine diphosphate	HMDB	Endo	16.43149631	2	Purine Nucleotides	0	224	146-91-8
6804	Guanosine monophosphate	HMDB	Endo	0.009698345	2	Purine Nucleotides	0	48	85-32-5,25191-14-4,29593-02-0,573-48-8,642-41-1
6830	Guanosine triphosphate	HMDB	Endo	56.00270538	1	Purine Nucleotides	0	305	86-01-1,56001-37-7
53477714	Heparan sulfate	HMDB	Endo	0.500023591	1	Disaccharides	9	0	9050-30-0
5283211	Hepoxilin A3	HMDB	Endo	0.113995079	1	Eicosanoids	0	5	85589-24-8
10465	Heptadecanoic acid	HMDB	Food	1.505913966	2	Fatty Acids and Conjugates	2	0	506-12-7
53477856	Heptaglutamyl folic acid	HMDB	Endo	0.007600053	1	Pteridines and Derivatives	0	0	.
72281	Hesperetin	HMDB	Food	0.418323593	2	Flavonoids	0	0	520-33-2
8370	Hexachlorobenzene	NHANES	Pollut	0.000296371	1	Organo-chlorine Pesticides	12	0	118-74-1
10469	Hexacosanoic acid	HMDB	Endo	0.302159577	2	Fatty Acids and Conjugates	2	0	506-46-7
464	Hippuric acid	HMDB	Food	2.999963134	1	Amino Acids and Derivatives	0	9	495-69-2,66407-11-2,140480-84-8,21251-67-2,892119-18-5,892119-19-6
774	Histamine	HMDB	Endo	0.000670022	1	Azoles	12	31	51-45-6,65592-96-3
65137	Histidylproline diketopiperazine	HMDB	Endo	2.19993E-05	1	Piperazines	0	0	53109-32-3
778	Homocysteine	NHANES	Endo	8.210386507	1	Amino Acids and Derivatives	1469	19	454-29-5,454-28-4,6681-97-6

PC_CID	Chemical	Dataset	Source	Concentration (µM)	No. studies	Class	PubMed	Biosys	CAS
107712	Homocysteine thiolactone	HMDB	Endo	0.002820052	1	Thiolanes	3	0	14007-10-4,10593-85-8
780	Homogentisic acid	HMDB	Endo	0.042998072	1	Phenylacetic Acid Derivatives	0	4	451-13-8
9085	Homo-L-arginine	HMDB	Endo	2.37762003	3	Amino Acids and Derivatives	5	0	156-86-5,13094-78-5
1738	Homovanillic acid	HMDB	Endo	0.049011515	9	Phenols and Derivatives	1	3	306-08-1
7139	Homoveratric acid	HMDB	Endo	0.815217769	8	Phenols and Derivatives	0	0	93-40-3
37270	HpCDD	NHANES	Pollut	4.93669E-07	2	Chlorinated-p-dibenzo Dioxins	0	0	35822-46-9
38199	HpCDF	NHANES	Pollut	1.55115E-07	2	Chlorinated-p-dibenzo Dioxins	0	0	67562-39-4
29575	HxCDD	NHANES	Pollut	4.17117E-07	2	Chlorinated-p-dibenzo Dioxins	0	0	19408-74-3
24759	Hyaluronan	HMDB	Endo	0.052998058	1	Tetrasaccharides	0	3	29382-75-0,165324-65-2,37243-73-5,9039-38-7
3639	Hydrochlorothiazide	HMDB	Drug	0.299991841	1	Benzothiadiazines	81	1	58-93-5,125727-50-6,8049-49-8
107	Hydrocinnamic acid	HMDB	Food	1.298876942	8	Aromatic Homomonocyclic Compounds	0	1	501-52-0
769	Hydrogen carbonate	HMDB	Endo	24219.17612	3	Organic Carbonic Acids and Derivatives	0	42	71-52-3
784	Hydrogen peroxide	HMDB	Endo	10.5002598	1	Non-metal Oxoanionic Compounds	13	100	7722-84-1,218625-72-0,37355-84-3,66554-50-5,8007-30-5
402	Hydrogen sulfide	HMDB	Endo	0.333170803	2	Other Non-metal Organides	3	18	7704-34-9,7783-06-4,9035-99-8,15117-53-0,11144-15-3,12673-82-4,12684-31-0,12767-24-7,37331-50-3,56449-52-6,56591-09-4,56645-30-8,57035-13-9,63705-05-5,7782-45-8,8050-82-6,81032-32-8,97124-07-7
53481617	Hydroxybutyrylcarnitine	HMDB	Endo	0.10999724	1	Fatty Acid Esters	0	0	.
414	Hydroxycotinine	HMDB	Pollut	0.295791637	2	Alkaloids and Derivatives	0	1	27323-64-4
89	Hydroxykynurenine	HMDB	Endo	0.050001614	1	Amino Acids and Derivatives	1	2	2147-61-7,484-78-6,606-14-4
9378	Hydroxyphenyllactic acid	HMDB	Endo	0.639991746	1	Benzyl Alcohols and Derivatives	0	2	306-23-0
69248	Hydroxyproline	HMDB	Endo	14.88419631	4	Amino Acids and Derivatives	2	9	30724-02-8
68152	Hydroxypropionic acid	HMDB	Endo	2.299979017	1	Hydroxy Acids and Derivatives	0	6	503-66-2
82755	Hydroxytyrosol	HMDB	Food	0.159997034	1	Phenols and Derivatives	2	0	10597-60-1

PC_CID	Chemical	Dataset	Source	Concentration (μM)	No. studies	Class	PubMed	Biosys	CAS
790	Hypoxanthine	HMDB	Endo	3.452850079	8	Imidazopyrimidines	2	25	68-94-0,146469-94-5,146469-95-6,51953-23-2,184856-40-4,184856-41-5,25991-07-5,25991-08-6,25991-09-7,39464-15-8,39464-17-0,480-99-9,51953-04-9,6535-89-3,95121-06-5
3672	Ibuprofen	HMDB	Drug	241.9908704	1	Prenol Lipids	10	0	15687-27-1,58560-75-1,139466-08-3
96215	Imidazoleacetic acid	HMDB	Endo	0.099998509	1	Azoles	0	1	645-65-8
504	Imidazolone	HMDB	Endo	0.039999033	1	Imidazolines	0	1	1968-28-1
802	Indoleacetic acid	HMDB	Food	0.050001614	1	Indoles	1	2	87-51-4,6505-45-9,54692-39-6
92904	Indolelactic acid	HMDB	Endo	2.799945632	1	Indoles	0	1	1821-52-9
10258	Indoxyl sulfate	HMDB	Food	14.0005974	1	Indoles	2	0	487-94-5,130385-38-5
6021	Inosine	HMDB	Endo	0.349972745	4	Purine Nucleosides and Analogues	1	17	58-63-9,12712-98-0,132953-54-9,28861-88-3,292853-81-7,4181-51-5,691344-25-9,740029-83-8
8582	Inosinic acid	HMDB	Endo	62.99781228	1	Purine Nucleotides	1	16	131-99-7,30918-54-8,138240-72-9,25249-22-3,485-83-6,736074-82-1,801187-33-7
439744	Iodotyrosine	HMDB	Endo	0.000690013	1	Amino Acids and Derivatives	0	8	70-78-0
6590	Isobutyric acid	HMDB	Endo	2.299979017	1	Carboxylic Acids and Derivatives	0	1	79-31-2
1198	Isocitric acid	HMDB	Endo	6.00024319	1	Carboxylic Acids and Derivatives	0	9	320-77-4
736186	Isoferulic acid	HMDB	Food	0.173253403	8	Cinnamic Acid Derivatives	0	0	537-73-5
3776	Isopropyl alcohol	HMDB	Endo	83.29595595	1	Alcohols and Polyols	0	2	67-63-0,555-31-7,21388-65-8,5131-95-3
5281654	Isorhamnetin	HMDB	Food	0.042998072	1	Flavonoids	0	0	480-19-3
11552	Iso-Valeraldehyde	HMDB	Food	0.10999724	1	Carbonyl Compounds	0	0	590-86-3
1001	Isovaleric acid	HMDB	Food	1.599994193	1	Fatty Acids and Conjugates	0	13	64-04-0
6426851	Isovalerylcarnitine	HMDB	Food	0.13800022	1	Fatty Acid Esters	0	0	31023-24-2
5280863	Kaempferol	HMDB	Food	0.009999702	1	Flavonoids	1	1	520-18-3

PC_CID	Chemical	Dataset	Source	Concentration (µM)	No. studies	Class	PubMed	Biosys	CAS
446715	Keratan	HMDB	Endo	0.1400018	1	Tetrasaccharides	0	0	69992-87-6
70	Ketoleucine	HMDB	Endo	30.62755212	2	Fatty Acids and Conjugates	0	6	816-66-0
3845	Kynurenic acid	HMDB	Endo	0.022999104	1	Amino Acids and Derivatives	2	5	492-27-3,13593-94-7
89594	L(-)-Nicotine pestanal	HMDB	Pollut	0.366264327	3	Alkaloids and Derivatives	30	7	54-11-5,16760-37-5,13890-81-8,13890-82-9,551-13-3
439939	L-2-Hydroxyglutaric acid	HMDB	Endo	1.500052339	1	Hydroxy Acids and Derivatives	0	4	13095-48-2
18230	L-Acetylcarnitine	HMDB	Endo	5.785761584	3	Fatty Acid Esters	0	1	4398-79-2
20057304	Lactosylceramide (d18:1/12:0)	HMDB	Endo	4.500101716	1	Sphingolipids	1	0	4682-48-8
11333	Lactulose	HMDB	Drug	0.119995624	1	Disaccharides	1	0	4618-18-2,58166-24-8,29319-45-7,33980-82-4,40773-84-0,576-08-9
5950	L-Alanine	HMDB	Endo	436.592384	3	Amino Acids and Derivatives	38	53	56-41-7,115967-49-2,170805-71-7,18875-37-1,25191-17-7,6898-94-8,759445-89-1,787635-21-6,130380-93-7
99288	L-Alloisoleucine	HMDB	Endo	1.900087618	1	Amino Acids and Derivatives	0	0	1509-34-8
80283	L-Alpha-aminobutyric acid	HMDB	Endo	22.94270452	3	Amino Acids and Derivatives	0	0	1492-24-6
439195	L-Arabinose	HMDB	Endo	2.50002317	1	Monosaccharides	0	5	5328-37-0
439255	L-Arabitol	HMDB	Endo	1.999905641	1	Sugar Alcohols	0	2	7643-75-6
6322	L-Arginine	HMDB	Food	102.0537872	9	Amino Acids and Derivatives	286	65	74-79-3,1119-34-2,142-49-4,25212-18-4,4455-52-1,667422-95-9
6267	L-Asparagine	HMDB	Endo	45.57229655	6	Amino Acids and Derivatives	7	36	70-47-3,5794-13-8,7006-34-0,32640-57-6,328-41-6
5960	L-Aspartic acid	HMDB	Endo	20.62904429	3	Amino Acids and Derivatives	16	72	56-84-8,25608-40-6,39162-75-9,6899-03-2,155436-59-2,155436-61-6,155436-63-8,155436-65-0,181119-33-5,181119-34-6,221628-95-1,26834-87-7,27881-03-4,90819-17-3
93078	L-Aspartyl-L-phenylalanine	HMDB	Food	0.017999703	1	Amino Acids and Derivatives	0	0	13433-09-5
65728	Lathosterol	HMDB	Endo	9.89965591	1	Steroids and Steroid Derivatives	12	4	80-99-9
10917	L-Carnitine	HMDB	Endo	38.6559226	4	Alkylamines	11	14	541-15-1,44985-71-9,7634-98-2
439258	L-Cystathionine	HMDB	Endo	0.203599592	2	Amino Acids and Derivatives	3	13	56-88-2

PC_CID	Chemical	Dataset	Source	Concentration (µM)	No. studies	Class	PubMed	Biosys	CAS
5862	L-Cysteine	HMDB	Endo	71.74369671	3	Amino Acids and Derivatives	61	53	52-90-4,4371-52-2,62488-11-3
22833544	L-Cysteinylglycine disulfide	HMDB	Endo	13.60041082	1	Peptides	0	0	70555-24-7
67678	L-Cystine	HMDB	Endo	96.45725916	4	Amino Acids and Derivatives	4	7	56-89-3,24645-67-8
6047	L-Dopa	HMDB	Endo	0.007230117	1	Amino Acids and Derivatives	0	17	59-92-7,587-45-1,23734-74-9,25525-15-9,34241-25-3,72572-99-7,72573-00-3,88250-23-1,90638-38-3
5352425	Lead	NHANES	Pollut	0.065618343	6	Post-transition Metals	65	3	7439-92-1,54076-28-7,14701-27-0,15158-12-0,724427-66-1
5280492	Leukotriene B4	HMDB	Endo	0.001019646	3	Eicosanoids	8	25	71160-24-2
5283125	Leukotriene B5	HMDB	Endo	0.002498162	2	Eicosanoids	0	0	80445-66-5
5280493	Leukotriene C4	HMDB	Endo	0.000156493	3	Eicosanoids	0	23	72025-60-6,10-34-4
5280878	Leukotriene D4	HMDB	Endo	4.20017E-05	1	Eicosanoids	1	19	73836-78-9
5280879	Leukotriene E4	HMDB	Endo	3.54744E-05	3	Eicosanoids	1	18	75715-89-8
5280938	Leukotriene F4	HMDB	Endo	0.000305	1	Eicosanoids	0	3	.
149096	Levofloxacin	HMDB	Drug	0.134997368	1	Phenylpiperazines	6	1	100986-85-4
33032	L-Glutamic acid	HMDB	Endo	56.00830593	6	Amino Acids and Derivatives	21	131	56-86-0,25513-46-6,10549-13-0,138-16-9,24938-00-9,26717-13-5,6899-05-4,84960-48-5
6992086	L-Glutamine	HMDB	Endo	547.5205526	6	Amino Acids and Derivatives	33	21	56-85-9
6326776	L-Glyceric acid	HMDB	Endo	1.999905641	1	Sugar Acids and Derivatives	0	0	28305-26-2
3246938	L-Hexanoylcarnitine	HMDB	Endo	0.080002292	1	Fatty Acid Esters	0	0	22671-29-0
6274	L-Histidine	HMDB	Food	102.6063683	5	Amino Acids and Derivatives	6	35	71-00-1,7006-35-1,6027-02-7,150-35-6,155304-24-8,26062-48-6,35479-49-3,35558-59-9,45955-20-2,54166-13-1,736075-03-9
12647	L-Homoserine	HMDB	Endo	11.9999202	1	Amino Acids and Derivatives	0	6	672-15-1

PC_CID	Chemical	Dataset	Source	Concentration (µM)	No. studies	Class	PubMed	Biosys	CAS
727	Lindane	NHANES	Pollut	0.000208724	3	Chlorinated Organo-chlorine Pesticides	5	1	58-89-9,319-84-6,319-85-7,319-86-8,608-73-1,6108-10-7,20437-97-2,25897-48-7,39284-22-5,53529-37-6,55963-79-6,60291-32-9,6108-11-8,6108-12-9,6108-13-0,8007-42-9,8073-23-2,89609-19-8
5280450	Linoleic acid	NHANES	Food	3449.898192	1	Lineolic Acids and Derivatives	42	31	60-33-3,506-21-8,80969-37-5,8024-22-4,949900-18-9,98353-71-0
5283446	Linoleoyl ethanolamide	HMDB	Endo	13.70005709	1	Fatty Amides	0	0	68171-52-8
6450015	Linoleyl carnitine	HMDB	Endo	0.060000643	1	Fatty Acid Esters	0	0	36816-10-1
864	Lipoic acid	HMDB	Endo	0.077003839	1	Dithiolanes	0	6	1077-28-7
5280914	Lipoxin A4	HMDB	Endo	7.42406E-05	2	Eicosanoids	0	19	89663-86-5
6306	L-Isoleucine	HMDB	Food	67.80934383	5	Amino Acids and Derivatives	9	37	73-32-5,7004-09-3,34464-35-2,410078-51-2,501028-75-7,807611-99-0,959215-79-3
28486	Lithium	HMDB	Drug	0.1400018	1	Homogeneous Alkali Metal Compounds	0	6	17341-24-1
9903	Lithocholic acid	HMDB	Endo	0.329987666	1	Steroids and Steroid Derivatives	1	11	434-13-9
115245	Lithocholic acid glycine conjugate	HMDB	Endo	0.009000276	1	Steroids and Steroid Derivatives	0	0	474-74-8
53477716	Lithocholyltaurine	HMDB	Endo	1.05421878	2	Steroids and Steroid Derivatives	0	0	516-90-5
161166	L-Kynurenine	HMDB	Endo	1.751723219	3	Amino Acids and Derivatives	0	8	2922-83-0,16055-80-4
107689	L-Lactic acid	HMDB	Endo	1996.198698	5	Hydroxy Acids and Derivatives	65	27	50-21-5
6106	L-Leucine	HMDB	Food	150.2348896	5	Amino Acids and Derivatives	29	37	61-90-5,21675-61-6,25248-98-0,70-45-1,71000-80-1
5962	L-Lysine	HMDB	Food	221.0745555	5	Amino Acids and Derivatives	40	48	56-87-1,26714-32-9,10098-89-2,20166-34-1,12798-06-0,25104-18-1,280114-50-3,48050-57-3,6899-06-5
222656	L-Malic acid	HMDB	Endo	6.196595324	2	Carboxylic Acids and Derivatives	0	31	97-67-6,124501-05-9,498-37-3,6294-10-6,84781-39-5
6137	L-Methionine	HMDB	Food	29.27400732	5	Amino Acids and Derivatives	88	54	63-68-3,7005-18-7,58576-49-1,24425-78-3,26062-47-5,3654-96-4
11953814	L-Octanoylcarnitine	HMDB	Endo	0.166826655	2	Fatty Acid Esters	0	0	25243-95-2

PC_CID	Chemical	Dataset	Source	Concentration (µM)	No. studies	Class	PubMed	Biosys	CAS
3955	Loperamide	HMDB	Drug	0.002399968	1	Diphenylmethanes	0	6	53179-11-6
3957	Loratadine	HMDB	Drug	1.01999732	1	Piperidines	0	0	79794-75-5
16902	L-Palmitoylcarnitine	HMDB	Endo	0.112996323	1	Fatty Acid Esters	0	4	2364-67-2
6140	L-Phenylalanine	HMDB	Food	72.95917222	7	Amino Acids and Derivatives	12	46	63-91-2,67675-33-6,10549-09-4,3617-44-5,5297-02-9,801204-11-5,62056-68-2
145742	L-Proline	HMDB	Endo	188.4061491	5	Amino Acids and Derivatives	35	38	147-85-3,18875-45-1,37159-97-0,4305-67-3,4607-28-7
53481794	LPS core	HMDB	Endo	6.1479E-06	2	Polysaccharides	0	0	.
53481793	LPS with O-antigen	HMDB	Endo	3.31087E-06	3	Polysaccharides	0	0	.
14461866	L-Rhamnulose	HMDB	Food	0.00043699	1	Monosaccharides	0	1	14807-05-7
5951	L-Serine	HMDB	Endo	137.7031113	8	Amino Acids and Derivatives	19	60	56-45-1,25821-52-7
22253	L-Threo-2-pentulose	HMDB	Endo	8.999778807	1	Monosaccharides	0	1	527-50-4
6288	L-Threonine	HMDB	Food	148.4280012	6	Amino Acids and Derivatives	23	34	72-19-5,13095-55-1,25275-17-6,36676-50-3,7013-32-3
5461103	L-Thyronine	HMDB	Endo	0.289992563	1	Amino Acids and Derivatives	0	1	1596-67-4
6305	L-Tryptophan	HMDB	Food	52.84166556	5	Amino Acids and Derivatives	17	46	73-22-3,6912-86-3,80206-30-0
6057	L-Tyrosine	HMDB	Endo	81.20688222	6	Amino Acids and Derivatives	51	58	60-18-4,55520-40-6,140-43-2,1991-85-1,25619-78-7,46209-14-7
5281243	Lutein	NHANES	Food	0.242585756	1	Prenol Lipids	20	1	127-40-2
6287	L-Valine	HMDB	Food	233.4808358	5	Amino Acids and Derivatives	80	39	72-18-4,7004-03-7,16872-32-5
446925	Lycopene	NHANES	Food	0.693641516	1	Prenol Lipids	62	2	502-65-8
460604	LysoPC(14:0)	HMDB	Endo	3.616114114	3	Glycerophospholipids	0	0	20559-16-4
24779456	LysoPC(14:1(9Z))	HMDB	Endo	0.210999398	1	Glycerophospholipids	0	0	.
24779458	LysoPC(15:0)	HMDB	Endo	1.764028299	1	Glycerophospholipids	0	0	.
10917802	LysoPC(16:0)	HMDB	Endo	117.0147506	3	Glycerophospholipids	0	0	.
24779461	LysoPC(16:1(9Z))	HMDB	Endo	2.670324498	3	Glycerophospholipids	0	0	.
24779463	LysoPC(17:0)	HMDB	Endo	2.550016934	1	Glycerophospholipids	0	0	50930-23-9
24779527	LysoPC(18:0)	HMDB	Endo	47.85616741	3	Glycerophospholipids	0	0	.
53480465	LysoPC(18:1(11Z))	HMDB	Endo	7.499227386	3	Glycerophospholipids	0	0	.

PC_CID	Chemical	Dataset	Source	Concentration (µM)	No. studies	Class	PubMed	Biosys	CAS
16081932	LysoPC(18:1(9Z))	HMDB	Endo	35.36066344	3	Glycerophospholipids	0	0	3542-29-8
11005824	LysoPC(18:2(9Z,12Z))	HMDB	Endo	43.8642659	3	Glycerophospholipids	0	0	.
52924045	LysoPC(18:3(6Z,9Z,12Z))	HMDB	Endo	0.234007994	1	Glycerophospholipids	0	0	.
24779469	LysoPC(18:3(9Z,12Z,15Z))	HMDB	Endo	1.238994297	1	Glycerophospholipids	0	0	.
52924047	LysoPC(18:4(6Z,9Z,12Z,15Z))	HMDB	Endo	0.285989552	1	Glycerophospholipids	0	0	.
24779473	LysoPC(20:0)	HMDB	Endo	0.687976912	1	Glycerophospholipids	0	0	.
52924051	LysoPC(20:1(11Z))	HMDB	Endo	0.525975839	1	Glycerophospholipids	0	0	.
52924053	LysoPC(20:2(11Z,14Z))	HMDB	Endo	0.711983886	1	Glycerophospholipids	0	0	.
53480467	LysoPC(20:3(5Z,8Z,11Z))	HMDB	Endo	0.549910358	3	Glycerophospholipids	0	0	.
52924055	LysoPC(20:3(8Z,11Z,14Z))	HMDB	Endo	2.715836475	3	Glycerophospholipids	0	0	.
24779476	LysoPC(20:4(5Z,8Z,11Z,14Z))	HMDB	Endo	7.55795008	3	Glycerophospholipids	0	0	.
53480469	LysoPC(20:4(8Z,11Z,14Z,17Z))	HMDB	Endo	0.740744143	3	Glycerophospholipids	0	0	.
11757087	LysoPC(20:5(5Z,8Z,11Z,14Z,17Z))	HMDB	Endo	1.608014197	1	Glycerophospholipids	0	0	162440-04-2
24779479	LysoPC(22:0)	HMDB	Endo	0.42998713	1	Glycerophospholipids	0	0	.
53480471	LysoPC(22:1(13Z))	HMDB	Endo	0.568985687	1	Glycerophospholipids	0	0	.
52924059	LysoPC(22:2(13Z,16Z))	HMDB	Endo	0.094997947	1	Glycerophospholipids	0	0	.
52924039	LysoPC(22:4(7Z,10Z,13Z,16Z))	HMDB	Endo	0.063998221	1	Glycerophospholipids	0	0	.
53480473	LysoPC(22:5(4Z,7Z,10Z,13Z,16Z))	HMDB	Endo	0.10700283	1	Glycerophospholipids	0	0	.
53480475	LysoPC(22:5(7Z,10Z,13Z,16Z,19Z))	HMDB	Endo	0.742969712	1	Glycerophospholipids	0	0	.
10415542	LysoPC(22:6(4Z,7Z,10Z,13Z,16Z,19Z))	HMDB	Endo	2.780136424	1	Glycerophospholipids	0	0	.
24779481	LysoPC(24:0)	HMDB	Endo	0.210662069	3	Glycerophospholipids	0	0	.
53480477	LysoPC(24:1(15Z))	HMDB	Endo	0.727966681	1	Glycerophospholipids	0	0	.
.	LysoPC(P-16:0)	HMDB	Endo	0.928021851	1	Glycerophospholipids	.	.	.
.	LysoPC(P-18:0)	HMDB	Endo	0.066997498	1	Glycerophospholipids	.	.	.
24779513	LysoPC(P-18:1(9Z))	HMDB	Endo	0.093331277	2	Glycerophospholipids	0	0	.
7809	m/p-Xylene	NHANES	Pollut	0.001327577	3	Aromatic Homomonocyclic Compounds	0	1	106-42-3
888	Magnesium	HMDB	Food	832.9719765	1	Homogeneous Alkaline Earth Metal Compounds	135	209	22537-22-0,71763-40-1
10964	Malondialdehyde	HMDB	Endo	1.276089087	2	Carbonyl Compounds	122	5	542-78-9
867	Malonic acid	HMDB	Endo	10.02818873	3	Carboxylic Acids and Derivatives	0	2	141-82-2

PC_CID	Chemical	Dataset	Source	Concentration (µM)	No. studies	Class	PubMed	Biosys	CAS
23930	Manganese	HMDB	Food	0.041585655	2	Homogeneous Transition Metal Compounds	2	105	7439-96-5,19768-33-3,13966-31-9,17375-02-9,195161-78-5,22325-60-6,39303-06-5,8031-40-1,8075-39-6
6251	Mannitol	HMDB	Food	34.00134219	1	Fatty Alcohols	10	2	69-65-8,87-78-5,123897-58-5,133-43-7,36413-61-3,5149-40-6,75398-80-0,85085-15-0
637541	m-Coumaric acid	HMDB	Food	0.028438825	8	Cinnamic Acid Derivatives	0	1	588-30-7
6325610	Melanin	HMDB	Endo	5184.869751	2	Indolequinones	0	1	8049-97-6
896	Melatonin	HMDB	Endo	6.14799E-05	2	Indoles	15	24	73-31-4
16666	Menthol	HMDB	Food	0.063998221	1	Prenol Lipids	0	0	2216-51-5
26623	Mercury	NHANES	Pollut	0.004097821	4	Homogeneous Transition Metal Compounds	21	0	14302-87-5
688084	Metanephrine	HMDB	Endo	0.001599923	1	Phenols and Derivatives	0	2	5001-33-2
878	Methanethiol	HMDB	Endo	5.700192807	1	Thiols	0	2	74-93-1
887	Methanol	HMDB	Endo	60.44295333	2	Alcohols and Polyols	1	3	67-56-1,31648-08-5,14742-26-8,54841-71-3
158980	Methionine sulfoxide	HMDB	Endo	4.000022556	1	Amino Acids and Derivatives	1	1	3226-65-1,86631-49-4
6329	Methylamine	HMDB	Endo	1	1	Alkylamines	0	5	74-89-5
24417	Methylcysteine	HMDB	Food	3.900091444	1	Amino Acids and Derivatives	0	0	1187-84-4
10111	Methylguanidine	HMDB	Endo	0.024999486	1	Guanidines	0	0	471-29-4
75810	Methylimidazoleacetic acid	HMDB	Endo	0.084567944	1	Azoles	0	1	2625-49-2
487	Methylmalonic acid	NHANES	Endo	0.134002074	1	Carboxylic Acids and Derivatives	14	3	516-05-2
15413	Methyl-t-butyl ether	NHANES	Pollut	0.117501991	3	Aliphatic Acyclic Compounds	0	0	1634-04-4
4171	Metoprolol	HMDB	Drug	0.070998254	1	Phenols and Derivatives	34	0	51384-51-1,37350-58-6,54163-88-1
439230	Mevalonic acid	HMDB	Endo	0.034697071	2	Hydroxy Acids and Derivatives	0	7	17817-88-8
5319879	MG(0:0/18:1(9Z)/0:0)	HMDB	Endo	166.0020271	1	Glycerolipids	0	0	3443-84-3
5365676	MG(0:0/18:2(9Z,12Z)/0:0)	HMDB	Endo	145.9990332	1	Lineolic Acids and Derivatives	0	0	3443-82-1
12178130	MG(18:1(9Z)/0:0/0:0)	HMDB	Endo	170.0002657	1	Glycerolipids	0	0	.
6436630	MG(18:2(9Z,12Z)/0:0/0:0)	HMDB	Endo	37.59984771	1	Lineolic Acids and Derivatives	0	0	107380-08-5
16019980	MG(20:4(5Z,8Z,11Z,14Z)/0:0/0:0)	HMDB	Endo	4.710056954	1	Eicosanoids	0	0	124511-15-5

PC_CID	Chemical	Dataset	Source	Concentration (µM)	No. studies	Class	PubMed	Biosys	CAS
16945	Mirex	HMDB	Pollut	4.00012E-05	1	Organo-chlorine Pesticides	1	0	2385-85-5,12557-88-9,12707-43-6,12766-04-0,20594-49-4,56449-78-6
185498	Molybdenum	HMDB	Food	0.006779174	2	Homogeneous Transition Metal Compounds	0	0	7439-98-7
92272	Monoisobutyl phthalic acid	HMDB	Food	0.002199995	1	Benzoic Acid and Derivatives	0	0	30833-53-5
11492	m-Tyramine	HMDB	Endo	3.80009E-05	1	Phenethylamines	0	0	588-05-6
892	Myoinositol	HMDB	Endo	23.06923704	4	Cyclic Alcohols and Derivatives	5	24	87-89-8,6917-35-7,643-12-9,488-59-5,551-72-4,488-55-1,643-10-7,488-58-4,173524-45-3,38876-99-2,41546-34-3,488-54-0,53319-35-0,576-63-6
890	Myo-inositol hexakisphosphate	HMDB	Endo	0.340003285	1	Cyclic Alcohols and Derivatives	2	11	83-86-3,50762-79-3,78039-41-5,894854-44-5
5281672	Myricetin	HMDB	Food	45.00168799	1	Flavonoids	1	0	529-44-2
11005	Myristic acid	NHANES	Endo	118.9972044	1	Fatty Acids and Conjugates	0	14	544-63-8,32112-52-0,45184-05-2
5281119	Myristoleic acid	NHANES	Endo	6.569909121	1	Fatty Acids and Conjugates	0	0	544-64-9
164795	N(6)-Methyllysine	HMDB	Endo	6.999928957	1	Amino Acids and Derivatives	0	0	1188-07-4
496	N1-Acetylspermidine	HMDB	Endo	0.007000316	1	Carboxylic Acids and Derivatives	0	8	14278-49-0
92919	N2,N2-Dimethylguanosine	HMDB	Endo	0.029983741	2	Purine Nucleosides and Analogues	0	0	2140-67-2
143	N5-Formyl-THF	HMDB	Endo	0.002499911	1	Pteridines and Derivatives	52	4	54353-24-1,58-05-9,121451-09-0
123689	N8-Acetylspermidine	HMDB	Endo	0.050001614	1	Carboxylic Acids and Derivatives	0	0	34450-15-2
67427	N-a-Acetyl-L-arginine	HMDB	Endo	1.249945562	1	Amino Acids and Derivatives	0	0	155-84-0
5255	N-Acetylaspartylglutamic acid	HMDB	Endo	4.890037636	1	Peptides	0	0	3106-85-2
439197	N-Acetylneuraminic acid	HMDB	Endo	1.067692737	2	Sugar Acids and Derivatives	45	6	131-48-6
439232	N-Acetylornithine	HMDB	Endo	1.099988802	1	Amino Acids and Derivatives	0	5	6205-08-9
15938971	NAD	HMDB	Endo	24.0011081	1	Purine Nucleotides	1	87	53-84-9
21604869	NADH	HMDB	Endo	21.99906605	1	Purine Nucleotides	0	80	58-68-4
5884	NADPH	HMDB	Endo	50.99869275	1	Purine Nucleotides	0	118	53-57-6,2646-71-1,22046-90-8,3545-01-5

PC_CID	Chemical	Dataset	Source	Concentration (µM)	No. studies	Class	PubMed	Biosys	CAS
9904	Nandrolone	HMDB	Endo	0.014500096	1	Steroids and Steroid Derivatives	1	0	434-22-0
156391	Naproxen	HMDB	Drug	301.9918409	1	Acenes	3	0	22204-53-1
5283389	N-Arachidonoyl glycine	HMDB	Endo	1.090024311	1	Amino Acids and Derivatives	0	6	.
439246	Naringenin	HMDB	Food	0.02000046	1	Flavonoids	0	1	480-41-1
448839	Neopterin	HMDB	Endo	0.006993319	3	Pteridines and Derivatives	40	1	2009-64-5
5281120	Nervonic acid	NHANES	Endo	74.90345368	1	Fatty Acids and Conjugates	2	1	506-37-6
513472	Neuraminic acid	HMDB	Endo	0.570010783	1	Sugar Acids and Derivatives	0	0	114-04-5
936	Niacinamide	HMDB	Endo	0.439991443	1	Pyridines and Derivatives	10	16	98-92-0,123574-63-0,37321-14-5,78731-47-2,11032-50-1,55600-01-6,63748-44-7
934	Nickel	HMDB	Pollut	0.066810168	4	Homogeneous Transition Metal Compounds	7	1	14701-22-5
72661	Nicotinamide N-oxide	HMDB	Endo	282.9867283	1	Pyridines and Derivatives	0	0	1986-81-8
938	Nicotinic acid	HMDB	Food	51.11101338	2	Pyridines and Derivatives	35	16	59-67-6,123574-58-3
68499	Nicotinuric acid	HMDB	Endo	5.770738143	2	Amino Acids and Derivatives	0	1	583-08-4
944	Nitrate	HMDB	Endo	39.6979679	2	Non-metal Oxoanionic Compounds	0	8	7697-37-2
145068	Nitric oxide	HMDB	Endo	1.2E-05	1	Other Non-metal Organides	223	34	10102-43-9
946	Nitrite	HMDB	Endo	8.667669897	4	Non-metal Oxoanionic Compounds	0	4	14797-65-0
5283454	N-Oleoylethanolamine	HMDB	Endo	0.410450476	2	Fatty Amides	0	0	111-58-0
5312513	Nonadeca-10(Z)-enoic acid	HMDB	Endo	0.645971431	1	Fatty Acids and Conjugates	0	0	67228-95-9
53481660	Nonanoylcarnitine	HMDB	Endo	0.029998737	1	Fatty Acid Esters	0	0	.
92223	Norcotinine	HMDB	Pollut	0.051001512	1	Pyrrolidinylpyridines	0	2	5980-06-3
439260	Norepinephrine	HMDB	Endo	0.0026439	3	Phenols and Derivatives	117	48	51-41-2,4899-05-2,66197-73-7
123747	Norepinephrine sulfate	HMDB	Endo	0.007937158	2	Phenols and Derivatives	0	0	77469-51-3
1237	Normetanephrine	HMDB	Endo	0.000326004	3	Phenols and Derivatives	5	1	97-31-4,17484-20-7
126923	N-Ribosylhistidine	HMDB	Endo	0.072999696	1	Amino Acids and Derivatives	0	0	98379-91-0
18636	OCDD	NHANES	Pollut	5.02995E-06	1	Chlorinated-p-dibenzo Dioxins	0	0	3268-87-9
89472	O-Desmethylangolensin	HMDB	Food	0.000618694	2	Stilbenes	2	0	21255-69-6
10494	Oleanolic acid	HMDB	Endo	0.025998927	1	Prenol Lipids	1	0	508-02-1

PC_CID	Chemical	Dataset	Source	Concentration (µM)	No. studies	Class	PubMed	Biosys	CAS
445639	Oleic acid	NHANES	Endo	2100.01549	1	Fatty Acids and Conjugates	25	29	112-80-1,17156-84-2,56833-51-3,68412-07-7,8046-01-3,949900-16-7
6436908	Oleoyl glycine	HMDB	Endo	20.99952881	1	Amino Acids and Derivatives	0	0	2601-90-3
4594	Omeprazole	HMDB	Drug	0.340003285	1	Benzimidazoles	7	5	73590-58-6,119141-89-8,131959-78-9,172964-80-6,119141-88-7,161796-78-7
1015	O-Phosphoethanolamine	HMDB	Endo	4.000022556	1	Organic Phosphoric Acids and Derivatives	0	13	1071-23-4
134490	Ornithine	HMDB	Endo	71.35732489	6	Amino Acids and Derivatives	0	15	1758-80-1
967	Orotic acid	HMDB	Endo	2.109603468	2	Amino Acids and Derivatives	1	7	65-86-1,50887-69-9
92751	Orotidine	HMDB	Endo	148.9931006	1	Pyrimidine Nucleosides and Analogues	0	0	314-50-1
11970	Ortho-Hydroxyphenylacetic acid	HMDB	Food	0.324263118	8	Phenylacetic Acid Derivatives	0	2	614-75-5
971	Oxalic acid	HMDB	Drug	10.95066297	3	Carboxylic Acids and Derivatives	1	5	144-62-7,1068-63-9,2847-15-6,547-66-0,553-91-3,216451-38-6,63504-28-9
65359	Oxidized glutathione	HMDB	Endo	1.689951787	1	Peptides	7	19	27025-41-8,10421-65-5
51	Oxoglutaric acid	HMDB	Endo	7.89318714	2	Keto-Acids and Derivatives	0	75	328-50-7,27175-99-1,34410-46-3
46174053	Oxychlordane	NHANES	Pollut	0.000152782	2	Organo-chlorine Pesticides	1	0	27304-13-8
7237	o-Xylene	NHANES	Pollut	0.00034849	1	Aromatic Homomonocyclic Compounds	0	1	95-47-6
4644	Oxypurinol	HMDB	Endo	49.99884974	1	Imidazopyrimidines	0	0	2465-59-0
46891879	PA(16:0/16:0)	HMDB	Endo	0.186001601	1	Glycerophospholipids	0	0	169051-60-9
9547158	PA(16:0/18:1(11Z))	HMDB	Endo	0.147003336	1	Glycerophospholipids	0	0	.
46891859	PA(16:0/18:2(9Z,12Z))	HMDB	Endo	0.144006045	1	Glycerophospholipids	0	0	322647-59-6
46891863	PA(18:0/18:2(9Z,12Z))	HMDB	Endo	0.222996322	1	Glycerophospholipids	0	0	474943-29-8
53478602	PA(18:1(11Z)/18:1(11Z))	HMDB	Endo	0.222996322	1	Glycerophospholipids	0	0	.
5282745	Palmitelaidic acid	HMDB	Food	1.969933922	1	Fatty Acids and Conjugates	10	0	2091-29-4,10030-73-6,373-49-9
985	Palmitic acid	NHANES	Endo	2709.989393	1	Fatty Acids and Conjugates	12	60	57-10-3,116860-99-2,212625-86-0,60605-23-4,66321-94-6,67701-02-4
445638	Palmitoleic acid	NHANES	Endo	217.0005743	1	Fatty Acids and Conjugates	10	10	373-49-9
4671	Palmitoylethanolamide	HMDB	Endo	0.571209064	2	Fatty Amides	0	0	544-31-0

PC_CID	Chemical	Dataset	Source	Concentration (µM)	No. studies	Class	PubMed	Biosys	CAS
978	p-Aminobenzoic acid	HMDB	Drug	15.000747	1	Benzoic Acid and Derivatives	0	6	150-13-0
4679	Pantoprazole	HMDB	Drug	10.00014907	1	Benzimidazoles	0	0	102625-70-7
988	Pantothenic acid	HMDB	Food	3.747166671	2	Alkylamines	0	8	79-83-4
4687	Paraxanthine	HMDB	Endo	10.00014907	1	Imidazopyrimidines	0	10	611-59-6
42948	PBB153	NHANES	Pollut	2.43885E-05	1	Brominated Biphenyls	0	0	59080-40-9
53478671	PC(16:0/16:0)	HMDB	Endo	584.5837176	1	Glycerophospholipids	0	0	63-89-8
37807	PCB101	NHANES	Pollut	3.37848E-05	1	Chlorinated Biphenyls	.	.	37680-73-2
36188	PCB105	NHANES	Pollut	2.45721E-05	1	Chlorinated Biphenyls	.	.	32598-14-4
38015	PCB110	NHANES	Pollut	2.49809E-05	1	Chlorinated Biphenyls	.	.	38380-03-9
35823	PCB118	NHANES	Pollut	0.000122856	1	Chlorinated Biphenyls	.	.	31508-00-6
63090	PCB126	NHANES	Pollut	3.93888E-07	2	Chlorinated Biphenyls	.	.	57465-28-8
37035	PCB138_158	NHANES	Pollut	0.000321054	2	Chlorinated Biphenyls	.	.	35065-28-2
40234	PCB146	NHANES	Pollut	4.01897E-05	1	Chlorinated Biphenyls	.	.	52112-04-6
38016	PCB149	NHANES	Pollut	1.10763E-05	1	Chlorinated Biphenyls	.	.	38380-04-0
37034	PCB153	NHANES	Pollut	0.000429839	2	Chlorinated Biphenyls	.	.	35065-27-1
38019	PCB156	NHANES	Pollut	4.70453E-05	1	Chlorinated Biphenyls	.	.	38380-08-4
50891	PCB157	NHANES	Pollut	1.12056E-05	1	Chlorinated Biphenyls	.	.	69782-90-7
40479	PCB167	NHANES	Pollut	9.1496E-06	1	Chlorinated Biphenyls	.	.	52663-72-6
36231	PCB169	NHANES	Pollut	0.000331527	1	Chlorinated Biphenyls	.	.	32774-16-6,1336-36-3
37037	PCB170	NHANES	Pollut	9.23154E-05	1	Chlorinated Biphenyls	.	.	35065-30-6
40481	PCB172	NHANES	Pollut	1.09398E-05	1	Chlorinated Biphenyls	.	.	52663-74-8
40477	PCB177	NHANES	Pollut	1.91062E-05	1	Chlorinated Biphenyls	.	.	52663-70-4
40474	PCB178	NHANES	Pollut	1.57747E-05	1	Chlorinated Biphenyls	.	.	52663-67-9
37036	PCB180	NHANES	Pollut	0.000287899	2	Chlorinated Biphenyls	.	.	35065-29-3
40476	PCB183	NHANES	Pollut	2.45156E-05	1	Chlorinated Biphenyls	.	.	52663-69-1
40475	PCB187	NHANES	Pollut	7.15224E-05	1	Chlorinated Biphenyls	.	.	52663-68-0
37251	PCB194	NHANES	Pollut	4.18382E-05	1	Chlorinated Biphenyls	.	.	31472-83-0
40485	PCB195	NHANES	Pollut	1.24424E-05	1	Chlorinated Biphenyls	.	.	52663-78-2
39253	PCB196_203	NHANES	Pollut	5.24107E-05	1	Chlorinated Biphenyls	.	.	42740-50-1
40482	PCB199	NHANES	Pollut	4.37027E-05	1	Chlorinated Biphenyls	.	.	52663-75-9
38411	PCB206	NHANES	Pollut	3.06708E-05	1	Chlorinated Biphenyls	.	.	40186-72-9

PC_CID	Chemical	Dataset	Source	Concentration (µM)	No. studies	Class	PubMed	Biosys	CAS
16318	PCB209	NHANES	Pollut	2.01582E-05	1	Chlorinated Biphenyls	.	.	2051-24-3
23448	PCB28	NHANES	Pollut	0.000127168	1	Chlorinated Biphenyls	.	.	7012-37-5
38875	PCB44	NHANES	Pollut	4.71583E-05	1	Chlorinated Biphenyls	.	.	41464-39-5
38876	PCB49	NHANES	Pollut	2.95301E-05	1	Chlorinated Biphenyls	.	.	41464-40-8
37248	PCB52	NHANES	Pollut	6.08925E-05	1	Chlorinated Biphenyls	.	.	35693-99-3
36185	PCB66	NHANES	Pollut	3.18173E-05	1	Chlorinated Biphenyls	.	.	32598-10-0
36218	PCB74	NHANES	Pollut	0.000110102	1	Chlorinated Biphenyls	.	.	32690-93-0
38014	PCB87	NHANES	Pollut	1.34329E-05	1	Chlorinated Biphenyls	.	.	38380-02-8
38013	PCB99	NHANES	Pollut	8.51837E-05	1	Chlorinated Biphenyls	.	.	38380-01-7
.	PCBs (all)	NHANES	Pollut	.	.	Chlorinated Biphenyls	92	0	.
637542	p-coumaric acid	HMDB	Food	0.114211875	8	Cinnamic Acid Derivatives	0	3	7400-08-0
13849	Pentadecanoic acid	HMDB	Endo	1.330560125	3	Fatty Acids and Conjugates	0	18	1002-84-2
9555	Perfluorodecanoic acid	NHANES	Pollut	0.00059295	3	Perfluorinated Compounds	0	0	335-76-2
67734	Perfluorohexane sulfonic acid	NHANES	Pollut	0.004647155	5	Perfluorinated Compounds	0	0	355-46-4
67821	Perfluorononanoic acid	NHANES	Pollut	0.002283605	5	Perfluorinated Compounds	0	0	375-95-1
74483	Perfluorooctane sulfonic acid	NHANES	Pollut	0.033517084	5	Perfluorinated Compounds	2	0	1763-23-1,132324-11-9
9554	Perfluorooctanoic acid	NHANES	Pollut	0.00965287	5	Perfluorinated Compounds	4	0	335-67-1,2395-00-8,335-93-3,65618-66-8,71244-15-0
5289145	PG(16:0/18:1(11Z))	HMDB	Endo	0.603988573	1	Glycerophospholipids	0	0	.
52927246	PG(16:0/18:2(9Z,12Z))	HMDB	Endo	0.080002292	1	Glycerophospholipids	0	0	.
53480603	PG(16:0/20:3(5Z,8Z,11Z))	HMDB	Endo	0.338003172	1	Glycerophospholipids	0	0	.
24779550	PG(16:0/20:4(5Z,8Z,11Z,14Z))	HMDB	Endo	0.21699087	1	Glycerophospholipids	0	0	.
52927231	PG(16:0/22:4(7Z,10Z,13Z,16Z))	HMDB	Endo	0.190996534	1	Glycerophospholipids	0	0	.
53480604	PG(16:0/22:5(4Z,7Z,10Z,13Z,16Z))	HMDB	Endo	0.605016227	1	Glycerophospholipids	0	0	.
52926307	PG(16:0/22:6(4Z,7Z,10Z,13Z,16Z,19Z))	HMDB	Endo	0.918971655	1	Glycerophospholipids	0	0	.
52926476	PG(16:1(9Z)/18:0)	HMDB	Endo	0.603988573	1	Glycerophospholipids	0	0	.
53480606	PG(16:1(9Z)/18:1(11Z))	HMDB	Endo	0.080002292	1	Glycerophospholipids	0	0	.
53480607	PG(16:1(9Z)/20:3(5Z,8Z,11Z))	HMDB	Endo	0.21699087	1	Glycerophospholipids	0	0	.
52927164	PG(16:1(9Z)/20:4(5Z,8Z,11Z,14Z))	HMDB	Endo	0.350989139	1	Glycerophospholipids	0	0	.
52926490	PG(16:1(9Z)/22:4(7Z,10Z,13Z,16Z))	HMDB	Endo	0.605016227	1	Glycerophospholipids	0	0	.
53480608	PG(16:1(9Z)/22:5(4Z,7Z,10Z,13Z,16Z))	HMDB	Endo	0.918971655	1	Glycerophospholipids	0	0	.
52927152	PG(18:0/16:1(9Z))	HMDB	Endo	0.603988573	1	Glycerophospholipids	0	0	.

PC_CID	Chemical	Dataset	Source	Concentration (μM)	No. studies	Class	PubMed	Biosys	CAS
53480610	PG(18:0/18:1(11Z))	HMDB	Endo	1.589945915	1	Glycerophospholipids	0	0	.
52927245	PG(18:0/18:2(9Z,12Z))	HMDB	Endo	0.193999441	1	Glycerophospholipids	0	0	.
52926580	PG(18:0/18:3(6Z,9Z,12Z))	HMDB	Endo	0.338003172	1	Glycerophospholipids	0	0	.
24779553	PG(18:0/20:4(5Z,8Z,11Z,14Z))	HMDB	Endo	0.190996534	1	Glycerophospholipids	0	0	.
52927148	PG(18:0/22:4(7Z,10Z,13Z,16Z))	HMDB	Endo	0.323000955	1	Glycerophospholipids	0	0	.
53480612	PG(18:0/22:5(4Z,7Z,10Z,13Z,16Z))	HMDB	Endo	0.158992221	1	Glycerophospholipids	0	0	.
24779554	PG(18:0/22:6(4Z,7Z,10Z,13Z,16Z,19Z))	HMDB	Endo	0.145003123	1	Glycerophospholipids	0	0	.
53480614	PG(18:1(11Z)/16:0)	HMDB	Endo	0.603988573	1	Glycerophospholipids	0	0	.
53480615	PG(18:1(11Z)/16:1(9Z))	HMDB	Endo	0.080002292	1	Glycerophospholipids	0	0	.
53480616	PG(18:1(11Z)/18:0)	HMDB	Endo	1.589945915	1	Glycerophospholipids	0	0	.
53480617	PG(18:1(11Z)/18:1(11Z))	HMDB	Endo	0.193999441	1	Glycerophospholipids	0	0	.
53480619	PG(18:1(11Z)/18:2(9Z,12Z))	HMDB	Endo	0.338003172	1	Glycerophospholipids	0	0	.
53480620	PG(18:1(11Z)/18:3(6Z,9Z,12Z))	HMDB	Endo	0.21699087	1	Glycerophospholipids	0	0	.
53480622	PG(18:1(11Z)/20:3(5Z,8Z,11Z))	HMDB	Endo	0.190996534	1	Glycerophospholipids	0	0	.
53480624	PG(18:1(11Z)/20:4(5Z,8Z,11Z,14Z))	HMDB	Endo	0.605016227	1	Glycerophospholipids	0	0	.
53480625	PG(18:1(11Z)/22:4(7Z,10Z,13Z,16Z))	HMDB	Endo	0.158992221	1	Glycerophospholipids	0	0	.
53480626	PG(18:1(11Z)/22:5(4Z,7Z,10Z,13Z,16Z))	HMDB	Endo	0.145003123	1	Glycerophospholipids	0	0	.
53480628	PG(18:1(11Z)/22:6(4Z,7Z,10Z,13Z,16Z,19Z))	HMDB	Endo	0.180992443	1	Glycerophospholipids	0	0	.
52927146	PG(18:1(9Z)/16:1(9Z))	HMDB	Endo	0.080002292	1	Glycerophospholipids	0	0	.
52927224	PG(18:1(9Z)/18:0)	HMDB	Endo	1.589945915	1	Glycerophospholipids	0	0	.
52927142	PG(18:2(9Z,12Z)/16:0)	HMDB	Endo	0.080002292	1	Glycerophospholipids	0	0	.
52927141	PG(18:2(9Z,12Z)/18:0)	HMDB	Endo	0.193999441	1	Glycerophospholipids	0	0	.
53480633	PG(18:2(9Z,12Z)/18:1(11Z))	HMDB	Endo	0.338003172	1	Glycerophospholipids	0	0	.
52927219	PG(18:2(9Z,12Z)/18:2(9Z,12Z))	HMDB	Endo	0.21699087	1	Glycerophospholipids	0	0	.
52926616	PG(18:2(9Z,12Z)/18:3(6Z,9Z,12Z))	HMDB	Endo	0.350989139	1	Glycerophospholipids	0	0	.
53480634	PG(18:2(9Z,12Z)/20:3(5Z,8Z,11Z))	HMDB	Endo	0.605016227	1	Glycerophospholipids	0	0	.
52927140	PG(18:2(9Z,12Z)/20:4(5Z,8Z,11Z,14Z))	HMDB	Endo	0.918971655	1	Glycerophospholipids	0	0	.
52926630	PG(18:2(9Z,12Z)/22:4(7Z,10Z,13Z,16Z))	HMDB	Endo	0.145003123	1	Glycerophospholipids	0	0	.
53480635	PG(18:2(9Z,12Z)/22:5(4Z,7Z,10Z,13Z,16Z))	HMDB	Endo	0.180992443	1	Glycerophospholipids	0	0	.
52926310	PG(18:2(9Z,12Z)/22:6(4Z,7Z,10Z,13Z,16Z,19Z))	HMDB	Endo	0.104999468	1	Glycerophospholipids	0	0	.
52926642	PG(18:3(6Z,9Z,12Z)/18:0)	HMDB	Endo	0.338003172	1	Glycerophospholipids	0	0	.

PC_CID	Chemical	Dataset	Source	Concentration (µM)	No. studies	Class	PubMed	Biosys	CAS
53480637	PG(18:3(6Z,9Z,12Z)/18:1(11Z))	HMDB	Endo	0.21699087	1	Glycerophospholipids	0	0	.
52926644	PG(18:3(6Z,9Z,12Z)/18:2(9Z,12Z))	HMDB	Endo	0.350989139	1	Glycerophospholipids	0	0	.
53480638	PG(18:3(6Z,9Z,12Z)/20:3(5Z,8Z,11Z))	HMDB	Endo	0.918971655	1	Glycerophospholipids	0	0	.
52926660	PG(18:3(6Z,9Z,12Z)/22:4(7Z,10Z,13Z,16Z))	HMDB	Endo	0.180992443	1	Glycerophospholipids	0	0	.
53480639	PG(18:3(6Z,9Z,12Z)/22:5(4Z,7Z,10Z,13Z,16Z))	HMDB	Endo	0.104999468	1	Glycerophospholipids	0	0	.
71434303	PGD2 ethanolamide	HMDB	Endo	0.160992097	1	Fatty Amides	0	0	398138-28-8
53481911	PGF2a ethanolamide	HMDB	Endo	0.017300844	1	Fatty Amides	0	0	.
996	Phenol	HMDB	Endo	0.860019685	1	Phenols and Derivatives	0	11	108-95-2,100790-31-6,14534-23-7,27073-41-2,50356-25-7,61788-41-8,63496-48-0,73607-76-8,8002-07-1
999	Phenylacetic acid	HMDB	Endo	54.21729752	8	Phenylacetic Acid Derivatives	1	2	103-82-2,51146-16-8
6041	Phenylephrine	HMDB	Drug	2.299979017	1	Phenethylamines	13	0	59-42-7,1416-03-1
10297	Phenylpropanolamine	HMDB	Drug	1.999905641	1	Phenethylamines	0	2	492-41-1
997	Phenylpyruvic acid	HMDB	Endo	0.500023591	1	Benzyl Alcohols and Derivatives	0	5	156-06-9
1061	Phosphate	HMDB	Endo	379.0999915	1	Non-metal Oxoanionic Compounds	0	343	14265-44-2,264888-19-9
1005	Phosphoenolpyruvic acid	HMDB	Endo	17.40051842	1	Organic Oxoanionic Compounds	0	13	138-08-9
7339	Phosphoribosyl pyrophosphate	HMDB	Endo	4.899827498	1	Monosaccharides	0	24	7540-64-9,13270-65-0,13860-42-9,29321-99-1,62726-16-3,97-55-2
1004	Phosphoric acid	HMDB	Endo	949.9411165	2	Non-metal Oxoanionic Compounds	0	5	7664-38-2,1339-32-8,178560-73-1,28602-75-7,68891-72-5,71751-05-8,8017-16-1,9044-08-0
24404	Phosphorus	HMDB	Food	2358.308727	3	Homogeneous Other Non-metal Compounds	78	0	7723-14-0,7803-51-2,51457-48-8,13769-19-2,167076-44-0,29879-37-6
1014	Phosphorylcholine	HMDB	Endo	2.200093809	1	Alcohols and Polyols	9	13	3616-04-4
68841	Phosphoserine	HMDB	Endo	16.99977315	1	Amino Acids and Derivatives	0	11	407-41-0
127	p-Hydroxyphenylacetic acid	HMDB	Food	5.580620662	9	Phenols and Derivatives	0	2	156-38-7
26840	Phytanic acid	HMDB	Endo	3.599878158	2	Prenol Lipids	2	4	14721-66-5,18654-64-3
9963391	Phytoene	HMDB	Endo	0.130002708	1	Prenol Lipids	0	1	13920-14-4
6436722	Phytofluene	HMDB	Food	0.193398974	3	Prenol Lipids	0	1	540-05-6

PC_CID	Chemical	Dataset	Source	Concentration (µM)	No. studies	Class	PubMed	Biosys	CAS
52928334	PI(16:0/16:1(9Z))	HMDB	Endo	0.746022141	1	Glycerophospholipids	0	0	.
71296229	PI(16:0/18:0)	HMDB	Endo	0.472981029	1	Glycerophospholipids	0	0	.
53480051	PI(16:0/18:1(11Z))	HMDB	Endo	1.779976212	1	Glycerophospholipids	0	0	.
46891796	PI(16:0/18:2(9Z,12Z))	HMDB	Endo	2.640055659	1	Glycerophospholipids	0	0	.
52928395	PI(16:0/20:0)	HMDB	Endo	0.269011809	1	Glycerophospholipids	0	0	.
52927618	PI(16:0/20:2(11Z,14Z))	HMDB	Endo	4.290056916	1	Glycerophospholipids	0	0	.
53480052	PI(16:0/20:3(5Z,8Z,11Z))	HMDB	Endo	1.169995614	1	Glycerophospholipids	0	0	.
52928411	PI(16:0/20:4(5Z,8Z,11Z,14Z))	HMDB	Endo	1.269978537	1	Glycerophospholipids	0	0	.
52927621	PI(16:0/22:2(13Z,16Z))	HMDB	Endo	0.189001562	1	Glycerophospholipids	0	0	.
53480054	PI(16:0/22:3(10Z,13Z,16Z))	HMDB	Endo	1.829970781	1	Glycerophospholipids	0	0	.
53480055	PI(16:0/22:4(10Z,13Z,16Z,19Z))	HMDB	Endo	11.000052	1	Glycerophospholipids	0	0	.
53480056	PI(16:0/22:5(4Z,7Z,10Z,13Z,16Z))	HMDB	Endo	1.280051101	1	Glycerophospholipids	0	0	.
52927628	PI(16:1(9Z)/16:0)	HMDB	Endo	0.746022141	1	Glycerophospholipids	0	0	.
52927632	PI(16:1(9Z)/18:0)	HMDB	Endo	1.779976212	1	Glycerophospholipids	0	0	.
53480058	PI(16:1(9Z)/18:1(11Z))	HMDB	Endo	2.640055659	1	Glycerophospholipids	0	0	.
53480060	PI(16:2(9Z,12Z)/18:0)	HMDB	Endo	2.640055659	1	Glycerophospholipids	0	0	.
53480061	PI(16:2(9Z,12Z)/22:3(10Z,13Z,16Z))	HMDB	Endo	1.280051101	1	Glycerophospholipids	0	0	.
52928311	PI(18:0/16:0)	HMDB	Endo	0.472981029	1	Glycerophospholipids	0	0	.
52928310	PI(18:0/16:1(9Z))	HMDB	Endo	1.779976212	1	Glycerophospholipids	0	0	.
53480062	PI(18:0/16:2(9Z,12Z))	HMDB	Endo	2.640055659	1	Glycerophospholipids	0	0	.
42607490	PI(18:0/18:0)	HMDB	Endo	0.269011809	1	Glycerophospholipids	0	0	.
52928409	PI(18:0/18:2(9Z,12Z))	HMDB	Endo	4.290056916	1	Glycerophospholipids	0	0	.
52927736	PI(18:0/18:3(6Z,9Z,12Z))	HMDB	Endo	1.169995614	1	Glycerophospholipids	0	0	.
52928387	PI(18:0/20:2(11Z,14Z))	HMDB	Endo	0.189001562	1	Glycerophospholipids	0	0	.
53480063	PI(18:0/20:3(5Z,8Z,11Z))	HMDB	Endo	1.829970781	1	Glycerophospholipids	0	0	.
42607491	PI(18:0/20:4(5Z,8Z,11Z,14Z))	HMDB	Endo	11.000052	1	Glycerophospholipids	0	0	.
53480065	PI(18:0/22:4(10Z,13Z,16Z,19Z))	HMDB	Endo	0.450994561	1	Glycerophospholipids	0	0	.
53480066	PI(18:0/22:5(4Z,7Z,10Z,13Z,16Z))	HMDB	Endo	0.475019226	1	Glycerophospholipids	0	0	.
52927465	PI(18:0/22:6(4Z,7Z,10Z,13Z,16Z,19Z))	HMDB	Endo	0.539992515	1	Glycerophospholipids	0	0	.
53480068	PI(18:1(11Z)/16:0)	HMDB	Endo	1.779976212	1	Glycerophospholipids	0	0	.
53480069	PI(18:1(11Z)/16:1(9Z))	HMDB	Endo	2.640055659	1	Glycerophospholipids	0	0	.

PC_CID	Chemical	Dataset	Source	Concentration (µM)	No. studies	Class	PubMed	Biosys	CAS
53480070	PI(18:1(11Z)/18:1(11Z))	HMDB	Endo	4.290056916	1	Glycerophospholipids	0	0	.
53480072	PI(18:1(11Z)/18:2(9Z,12Z))	HMDB	Endo	1.169995614	1	Glycerophospholipids	0	0	.
53480073	PI(18:1(11Z)/18:3(6Z,9Z,12Z))	HMDB	Endo	1.269978537	1	Glycerophospholipids	0	0	.
53480075	PI(18:1(11Z)/20:1(11Z))	HMDB	Endo	0.189001562	1	Glycerophospholipids	0	0	.
53480076	PI(18:1(11Z)/20:3(5Z,8Z,11Z))	HMDB	Endo	11.000052	1	Glycerophospholipids	0	0	.
53480078	PI(18:1(11Z)/20:4(5Z,8Z,11Z,14Z))	HMDB	Endo	1.280051101	1	Glycerophospholipids	0	0	.
52928300	PI(18:2(9Z,12Z)/16:0)	HMDB	Endo	2.640055659	1	Glycerophospholipids	0	0	.
52928299	PI(18:2(9Z,12Z)/18:0)	HMDB	Endo	4.290056916	1	Glycerophospholipids	0	0	.
53480083	PI(18:2(9Z,12Z)/18:1(11Z))	HMDB	Endo	1.169995614	1	Glycerophospholipids	0	0	.
52928380	PI(18:2(9Z,12Z)/18:2(9Z,12Z))	HMDB	Endo	1.269978537	1	Glycerophospholipids	0	0	.
52927777	PI(18:2(9Z,12Z)/20:0)	HMDB	Endo	0.189001562	1	Glycerophospholipids	0	0	.
52927778	PI(18:2(9Z,12Z)/20:1(11Z))	HMDB	Endo	1.829970781	1	Glycerophospholipids	0	0	.
52927779	PI(18:2(9Z,12Z)/20:2(11Z,14Z))	HMDB	Endo	11.000052	1	Glycerophospholipids	0	0	.
53480084	PI(18:2(9Z,12Z)/20:3(5Z,8Z,11Z))	HMDB	Endo	1.280051101	1	Glycerophospholipids	0	0	.
52927785	PI(18:2(9Z,12Z)/22:2(13Z,16Z))	HMDB	Endo	0.450994561	1	Glycerophospholipids	0	0	.
53480085	PI(18:2(9Z,12Z)/22:3(10Z,13Z,16Z))	HMDB	Endo	0.475019226	1	Glycerophospholipids	0	0	.
52927798	PI(18:3(6Z,9Z,12Z)/18:0)	HMDB	Endo	1.169995614	1	Glycerophospholipids	0	0	.
53480086	PI(18:3(6Z,9Z,12Z)/18:1(11Z))	HMDB	Endo	1.269978537	1	Glycerophospholipids	0	0	.
53480087	PI(18:3(6Z,9Z,12Z)/22:3(10Z,13Z,16Z))	HMDB	Endo	0.539992515	1	Glycerophospholipids	0	0	.
52928286	PI(20:0/16:0)	HMDB	Endo	0.269011809	1	Glycerophospholipids	0	0	.
52928375	PI(20:0/18:2(9Z,12Z))	HMDB	Endo	0.189001562	1	Glycerophospholipids	0	0	.
52927943	PI(20:0/20:3(8Z,11Z,14Z))	HMDB	Endo	0.21699087	1	Glycerophospholipids	0	0	.
52928284	PI(20:0/20:4(5Z,8Z,11Z,14Z))	HMDB	Endo	0.450994561	1	Glycerophospholipids	0	0	.
53480091	PI(20:1(11Z)/18:1(11Z))	HMDB	Endo	0.189001562	1	Glycerophospholipids	0	0	.
52927962	PI(20:1(11Z)/18:2(9Z,12Z))	HMDB	Endo	1.829970781	1	Glycerophospholipids	0	0	.
52927971	PI(20:1(11Z)/20:4(5Z,8Z,11Z,14Z))	HMDB	Endo	0.475019226	1	Glycerophospholipids	0	0	.
52927985	PI(20:2(11Z,14Z)/16:0)	HMDB	Endo	4.290056916	1	Glycerophospholipids	0	0	.
52927990	PI(20:2(11Z,14Z)/18:0)	HMDB	Endo	0.189001562	1	Glycerophospholipids	0	0	.
52927992	PI(20:2(11Z,14Z)/18:2(9Z,12Z))	HMDB	Endo	11.000052	1	Glycerophospholipids	0	0	.
52928000	PI(20:2(11Z,14Z)/20:2(11Z,14Z))	HMDB	Endo	0.450994561	1	Glycerophospholipids	0	0	.
53480093	PI(20:3(5Z,8Z,11Z)/16:0)	HMDB	Endo	1.169995614	1	Glycerophospholipids	0	0	.

PC_CID	Chemical	Dataset	Source	Concentration (µM)	No. studies	Class	PubMed	Biosys	CAS
53480094	PI(20:3(5Z,8Z,11Z)/18:0)	HMDB	Endo	1.829970781	1	Glycerophospholipids	0	0	.
53480095	PI(20:3(5Z,8Z,11Z)/18:1(11Z))	HMDB	Endo	11.000052	1	Glycerophospholipids	0	0	.
53480097	PI(20:3(5Z,8Z,11Z)/18:2(9Z,12Z))	HMDB	Endo	1.280051101	1	Glycerophospholipids	0	0	.
53480098	PI(20:3(5Z,8Z,11Z)/20:3(5Z,8Z,11Z))	HMDB	Endo	0.539992515	1	Glycerophospholipids	0	0	.
52928281	PI(20:4(5Z,8Z,11Z,14Z)/16:0)	HMDB	Endo	1.269978537	1	Glycerophospholipids	0	0	.
52928051	PI(20:4(5Z,8Z,11Z,14Z)/18:0)	HMDB	Endo	11.000052	1	Glycerophospholipids	0	0	.
53480102	PI(20:4(5Z,8Z,11Z,14Z)/18:1(11Z))	HMDB	Endo	1.280051101	1	Glycerophospholipids	0	0	.
52928059	PI(20:4(5Z,8Z,11Z,14Z)/20:0)	HMDB	Endo	0.450994561	1	Glycerophospholipids	0	0	.
52928060	PI(20:4(5Z,8Z,11Z,14Z)/20:1(11Z))	HMDB	Endo	0.475019226	1	Glycerophospholipids	0	0	.
52928184	PI(22:2(13Z,16Z)/16:0)	HMDB	Endo	0.189001562	1	Glycerophospholipids	0	0	.
52928191	PI(22:2(13Z,16Z)/18:2(9Z,12Z))	HMDB	Endo	0.450994561	1	Glycerophospholipids	0	0	.
53480109	PI(22:3(10Z,13Z,16Z)/16:0)	HMDB	Endo	1.829970781	1	Glycerophospholipids	0	0	.
53480110	PI(22:3(10Z,13Z,16Z)/16:2(9Z,12Z))	HMDB	Endo	1.280051101	1	Glycerophospholipids	0	0	.
53480111	PI(22:3(10Z,13Z,16Z)/18:2(9Z,12Z))	HMDB	Endo	0.475019226	1	Glycerophospholipids	0	0	.
53480112	PI(22:3(10Z,13Z,16Z)/18:3(6Z,9Z,12Z))	HMDB	Endo	0.539992515	1	Glycerophospholipids	0	0	.
53480114	PI(22:4(10Z,13Z,16Z,19Z)/16:0)	HMDB	Endo	11.000052	1	Glycerophospholipids	0	0	.
53480115	PI(22:4(10Z,13Z,16Z,19Z)/18:0)	HMDB	Endo	0.450994561	1	Glycerophospholipids	0	0	.
53480116	PI(22:5(4Z,7Z,10Z,13Z,16Z)/16:0)	HMDB	Endo	1.280051101	1	Glycerophospholipids	0	0	.
53480117	PI(22:5(4Z,7Z,10Z,13Z,16Z)/18:0)	HMDB	Endo	0.475019226	1	Glycerophospholipids	0	0	.
52928249	PI(22:6(4Z,7Z,10Z,13Z,16Z,19Z)/18:0)	HMDB	Endo	0.539992515	1	Glycerophospholipids	0	0	.
1018	Picolinic acid	HMDB	Endo	0.2990035	1	Amino Acids and Derivatives	1	0	98-98-6,32075-31-3,88161-53-9
6451814	Pi-Methylimidazoleacetic acid	HMDB	Endo	0.072999696	1	Azoles	0	0	4200-48-0
439227	Pipecolic acid	HMDB	Food	1.718067306	2	Amino Acids and Derivatives	0	2	3105-95-1
440266	p-Octopamine	HMDB	Endo	0.001396481	2	Phenethylamines	0	1	104-14-3
1021	Porphobilinogen	HMDB	Endo	0.060000643	1	Carboxylic Acids and Derivatives	0	6	487-90-1
813	Potassium	HMDB	Food	4149.736014	2	Homogeneous Alkali Metal Compounds	0	90	24203-36-9
8955	Pregnenolone	HMDB	Endo	0.004601836	12	Steroids and Steroid Derivatives	0	22	145-13-1,116907-59-6
105074	Pregnenolone sulfate	HMDB	Endo	0.130002708	1	Steroids and Steroid Derivatives	0	5	1247-64-9

PC_CID	Chemical	Dataset	Source	Concentration (µM)	No. studies	Class	PubMed	Biosys	CAS
5994	Progesterone	HMDB	Endo	0.003416632	5	Steroids and Steroid Derivatives	76	27	57-83-0,257630-50-5,753497-20-0,8012-32-6,8023-13-0
115244	Proline betaine	HMDB	Food	10.00014907	1	Pyrrolidines	0	0	471-87-4
1032	Propionic acid	HMDB	Food	0.899964465	1	Carboxylic Acids and Derivatives	0	17	79-09-4,68937-68-8,3349-08-4,68990-37-4,784139-72-6
107738	Propionylcarnitine	HMDB	Endo	0.352149316	2	Fatty Acid Esters	1	4	17298-37-2,25518-45-0
1031	Propyl alcohol	HMDB	Endo	0.329987666	1	Alcohols and Polyols	3	1	71-23-8,4712-36-1,142583-61-7,62309-51-7
1030	Propylene glycol	HMDB	Food	6.678544002	2	Alcohols and Polyols	0	2	57-55-6,190913-75-8,4254-16-4
5281912	Prostaglandin A1	HMDB	Endo	7.40034E-05	1	Eicosanoids	0	0	14152-28-4
5280880	Prostaglandin A2	HMDB	Endo	0.000419688	7	Eicosanoids	0	7	13345-50-1
5280881	Prostaglandin B2	HMDB	Endo	0.01391344	2	Eicosanoids	0	7	13367-85-6
5280936	Prostaglandin D1	HMDB	Endo	1.00003E-05	1	Eicosanoids	0	0	17968-82-0
448457	Prostaglandin D2	HMDB	Endo	0.001644038	3	Eicosanoids	3	24	41598-07-6
5282260	Prostaglandin D3	HMDB	Endo	0.000123003	1	Eicosanoids	0	0	71902-47-1
5280723	Prostaglandin E1	HMDB	Endo	3.69992E-06	1	Eicosanoids	11	6	745-65-3,119314-69-1,22299-37-2,50-83-9,50865-30-0
5280360	Prostaglandin E2	HMDB	Endo	0.003263353	6	Eicosanoids	17	29	363-24-6
5280939	Prostaglandin F1a	HMDB	Endo	0.000376008	1	Eicosanoids	1	0	745-62-0
5280363	Prostaglandin F2a	HMDB	Endo	0.006333514	4	Eicosanoids	46	23	551-11-1,13535-33-6,99437-94-2
5280884	Prostaglandin J2	HMDB	Endo	4.4089E-05	2	Eicosanoids	0	7	60203-57-8
72	Protocatechuic acid	HMDB	Food	0.304921778	9	Benzoic Acid and Derivatives	0	6	99-50-3
4971	Protoporphyrin IX	HMDB	Endo	0.591437065	2	Tetrapyrroles and Derivatives	1	6	553-12-8,50865-01-5,1818-68-4,227475-00-5,263148-77-2,37188-04-8,392312-74-2,42183-71-1,50312-31-7,562103-89-3,61320-51-2,63872-77-5,673437-82-6,68034-48-0,720672-66-2,75440-82-3,75460-41-2,75599-49-4,75750-98-0,942418-20-4

PC_CID	Chemical	Dataset	Source	Concentration (µM)	No. studies	Class	PubMed	Biosys	CAS
52926083	PS(14:0/18:1(9Z))	HMDB	Endo	0.238998023	1	Glycerophospholipids	0	0	.
52925229	PS(14:1(9Z)/18:0)	HMDB	Endo	0.238998023	1	Glycerophospholipids	0	0	.
52926017	PS(16:0/16:1(9Z))	HMDB	Endo	0.238998023	1	Glycerophospholipids	0	0	.
52926014	PS(16:0/18:0)	HMDB	Endo	0.307002312	1	Glycerophospholipids	0	0	.
5283499	PS(16:0/18:1(9Z))	HMDB	Endo	0.186001601	1	Glycerophospholipids	0	0	40290-44-6
46891801	PS(16:0/18:2(9Z,12Z))	HMDB	Endo	0.150996292	1	Glycerophospholipids	0	0	383908-63-2
52926011	PS(16:0/20:3(8Z,11Z,14Z))	HMDB	Endo	0.122996424	1	Glycerophospholipids	0	0	.
24779544	PS(16:0/20:4(5Z,8Z,11Z,14Z))	HMDB	Endo	0.178994536	1	Glycerophospholipids	0	0	.
46891804	PS(16:0/22:6(4Z,7Z,10Z,13Z,16Z,19Z))	HMDB	Endo	0.161993349	1	Glycerophospholipids	0	0	474943-17-4
52925317	PS(16:1(9Z)/16:0)	HMDB	Endo	0.238998023	1	Glycerophospholipids	0	0	.
52925321	PS(16:1(9Z)/18:0)	HMDB	Endo	0.186001601	1	Glycerophospholipids	0	0	.
52926009	PS(16:1(9Z)/18:1(9Z))	HMDB	Endo	0.150996292	1	Glycerophospholipids	0	0	.
52925329	PS(16:1(9Z)/20:3(8Z,11Z,14Z))	HMDB	Endo	0.178994536	1	Glycerophospholipids	0	0	.
52925419	PS(18:0/14:1(9Z))	HMDB	Endo	0.238998023	1	Glycerophospholipids	0	0	.
52925997	PS(18:0/16:0)	HMDB	Endo	0.307002312	1	Glycerophospholipids	0	0	.
52925996	PS(18:0/16:1(9Z))	HMDB	Endo	0.186001601	1	Glycerophospholipids	0	0	.
46891784	PS(18:0/18:0)	HMDB	Endo	2.090075103	1	Glycerophospholipids	0	0	321595-13-5
59720717	PS(18:0/18:1(9Z))	HMDB	Endo	0.73300702	1	Glycerophospholipids	0	.	321883-23-2
9547091	PS(18:0/18:2(9Z,12Z))	HMDB	Endo	0.316004129	1	Glycerophospholipids	0	0	.
52925426	PS(18:0/18:3(9Z,12Z,15Z))	HMDB	Endo	0.122996424	1	Glycerophospholipids	0	0	.
52925994	PS(18:0/20:3(8Z,11Z,14Z))	HMDB	Endo	0.193999441	1	Glycerophospholipids	0	0	.
46891807	PS(18:0/20:4(5Z,8Z,11Z,14Z))	HMDB	Endo	0.480994232	1	Glycerophospholipids	0	0	.
53480355	PS(18:0/22:5(7Z,10Z,13Z,16Z,19Z))	HMDB	Endo	0.113995079	1	Glycerophospholipids	0	0	.
46891808	PS(18:0/22:6(4Z,7Z,10Z,13Z,16Z,19Z))	HMDB	Endo	0.636990842	1	Glycerophospholipids	0	0	.
52925991	PS(18:1(9Z)/14:0)	HMDB	Endo	0.238998023	1	Glycerophospholipids	0	0	.
52926066	PS(18:1(9Z)/16:0)	HMDB	Endo	0.186001601	1	Glycerophospholipids	0	0	.
52925990	PS(18:1(9Z)/16:1(9Z))	HMDB	Endo	0.150996292	1	Glycerophospholipids	0	0	.
9547094	PS(18:1(9Z)/18:0)	HMDB	Endo	0.73300702	1	Glycerophospholipids	0	0	.
23692652	PS(18:1(9Z)/18:1(9Z))	HMDB	Endo	0.316004129	1	Glycerophospholipids	0	.	70614-14-1
52926065	PS(18:1(9Z)/18:2(9Z,12Z))	HMDB	Endo	0.122996424	1	Glycerophospholipids	0	0	.
52925989	PS(18:1(9Z)/18:3(9Z,12Z,15Z))	HMDB	Endo	0.178994536	1	Glycerophospholipids	0	0	.

PC_CID	Chemical	Dataset	Source	Concentration (µM)	No. studies	Class	PubMed	Biosys	CAS
52925445	PS(18:1(9Z)/20:3(8Z,11Z,14Z))	HMDB	Endo	0.480994232	1	Glycerophospholipids	0	0	.
52925988	PS(18:1(9Z)/20:4(5Z,8Z,11Z,14Z))	HMDB	Endo	0.281000171	1	Glycerophospholipids	0	0	.
52925155	PS(18:1(9Z)/22:6(4Z,7Z,10Z,13Z,16Z,19Z))	HMDB	Endo	0.168992659	1	Glycerophospholipids	0	0	.
52925986	PS(18:2(9Z,12Z)/16:0)	HMDB	Endo	0.150996292	1	Glycerophospholipids	0	0	.
52925985	PS(18:2(9Z,12Z)/18:0)	HMDB	Endo	0.316004129	1	Glycerophospholipids	0	0	.
52925460	PS(18:2(9Z,12Z)/18:1(9Z))	HMDB	Endo	0.122996424	1	Glycerophospholipids	0	0	.
46891793	PS(18:2(9Z,12Z)/18:2(9Z,12Z))	HMDB	Endo	0.178994536	1	Glycerophospholipids	0	0	.
52925469	PS(18:2(9Z,12Z)/20:3(8Z,11Z,14Z))	HMDB	Endo	0.281000171	1	Glycerophospholipids	0	0	.
52925984	PS(18:2(9Z,12Z)/20:4(5Z,8Z,11Z,14Z))	HMDB	Endo	0.161993349	1	Glycerophospholipids	0	0	.
52925517	PS(18:3(9Z,12Z,15Z)/18:0)	HMDB	Endo	0.122996424	1	Glycerophospholipids	0	0	.
52925983	PS(18:3(9Z,12Z,15Z)/18:1(9Z))	HMDB	Endo	0.178994536	1	Glycerophospholipids	0	0	.
52925526	PS(18:3(9Z,12Z,15Z)/20:3(8Z,11Z,14Z))	HMDB	Endo	0.161993349	1	Glycerophospholipids	0	0	.
52925705	PS(20:3(8Z,11Z,14Z)/16:0)	HMDB	Endo	0.122996424	1	Glycerophospholipids	0	0	.
52925706	PS(20:3(8Z,11Z,14Z)/16:1(9Z))	HMDB	Endo	0.178994536	1	Glycerophospholipids	0	0	.
52925710	PS(20:3(8Z,11Z,14Z)/18:0)	HMDB	Endo	0.193999441	1	Glycerophospholipids	0	0	.
52925711	PS(20:3(8Z,11Z,14Z)/18:1(9Z))	HMDB	Endo	0.480994232	1	Glycerophospholipids	0	0	.
52925712	PS(20:3(8Z,11Z,14Z)/18:2(9Z,12Z))	HMDB	Endo	0.281000171	1	Glycerophospholipids	0	0	.
52925714	PS(20:3(8Z,11Z,14Z)/18:3(9Z,12Z,15Z))	HMDB	Endo	0.161993349	1	Glycerophospholipids	0	0	.
52925721	PS(20:3(8Z,11Z,14Z)/20:3(8Z,11Z,14Z))	HMDB	Endo	0.636990842	1	Glycerophospholipids	0	0	.
52925722	PS(20:3(8Z,11Z,14Z)/20:4(5Z,8Z,11Z,14Z))	HMDB	Endo	0.168992659	1	Glycerophospholipids	0	0	.
52925968	PS(20:4(5Z,8Z,11Z,14Z)/16:0)	HMDB	Endo	0.178994536	1	Glycerophospholipids	0	0	.
52925740	PS(20:4(5Z,8Z,11Z,14Z)/18:0)	HMDB	Endo	0.480994232	1	Glycerophospholipids	0	0	.
52925741	PS(20:4(5Z,8Z,11Z,14Z)/18:1(9Z))	HMDB	Endo	0.281000171	1	Glycerophospholipids	0	0	.
52925742	PS(20:4(5Z,8Z,11Z,14Z)/18:2(9Z,12Z))	HMDB	Endo	0.161993349	1	Glycerophospholipids	0	0	.
52925751	PS(20:4(5Z,8Z,11Z,14Z)/20:3(8Z,11Z,14Z))	HMDB	Endo	0.168992659	1	Glycerophospholipids	0	0	.
52925956	PS(22:6(4Z,7Z,10Z,13Z,16Z,19Z)/16:0)	HMDB	Endo	0.161993349	1	Glycerophospholipids	0	0	.
52925937	PS(22:6(4Z,7Z,10Z,13Z,16Z,19Z)/18:0)	HMDB	Endo	0.636990842	1	Glycerophospholipids	0	0	.
52925938	PS(22:6(4Z,7Z,10Z,13Z,16Z,19Z)/18:1(9Z))	HMDB	Endo	0.168992659	1	Glycerophospholipids	0	0	.
7028	Pseudoephedrine	HMDB	Drug	3.020130372	1	Phenethylamines	0	2	90-82-4
15047	Pseudouridine	HMDB	Endo	3.180059795	1	Glycosyl Compounds	1	1	1445-07-4
7172	p-Synephrine	HMDB	Endo	0.000339988	1	Phenethylamines	0	0	94-07-5

PC_CID	Chemical	Dataset	Source	Concentration (μM)	No. studies	Class	PubMed	Biosys	CAS
1045	Putrescine	HMDB	Endo	0.213995563	1	Alkylamines	1	19	110-60-1
1050	Pyridoxal	HMDB	Endo	0.251000587	1	Pyridines and Derivatives	1	8	66-72-8,65-22-5
1051	Pyridoxal 5'-phosphate	NHANES	Endo	0.051400879	1	Pyridines and Derivatives	34	31	54-47-7,853645-22-4,52064-48-9,52441-27-7,41468-25-1
1052	Pyridoxamine	HMDB	Endo	0.163998172	1	Pyridines and Derivatives	0	7	85-87-0
1053	Pyridoxamine 5'-phosphate	HMDB	Endo	0.003834179	2	Pyridines and Derivatives	0	8	529-96-4
1054	Pyridoxine	HMDB	Endo	0.024999486	1	Pyridines and Derivatives	53	7	65-23-6,58-56-0
32819	Pyrocatechol	HMDB	Food	1.101640024	8	Phenols and Derivatives	1	1	3938-16-7
7405	Pyroglutamic acid	HMDB	Endo	19.49971792	1	Pyrrolidines	3	7	98-79-3,16891-48-8,29222-42-2,312618-42-1,35255-51-7,498-91-9,6886-28-8,87430-62-4,95650-42-3
1023	Pyrophosphate	HMDB	Endo	1.800024003	1	Non-metal Oxoanionic Compounds	0	274	2466-09-3
880	Pyruvaldehyde	HMDB	Endo	8.055861797	2	Carbonyl Compounds	7	6	78-98-8,51252-84-7
1060	Pyruvic acid	HMDB	Endo	62.12198798	7	Keto-Acids and Derivatives	2	64	127-17-3,1892-67-7,151677-69-9
5280343	Quercetin	HMDB	Food	0.042425741	2	Flavonoids	10	1	117-39-5,74893-81-5,7255-55-2,73123-10-1,849061-97-8
1066	Quinolinic acid	HMDB	Endo	0.470010615	1	Amino Acids and Derivatives	1	5	89-00-9,18970-62-2,28605-84-7,339155-13-4
439242	Raffinose	HMDB	Food	4.799923595	1	Trisaccharides	0	1	512-69-6
3001055	Ranitidine	HMDB	Drug	7.300187261	1	Furans	4	2	66357-35-5,68109-63-7,66357-59-3
44251266	Resolvin D1	HMDB	Endo	0.04540196	1	Fatty Acids and Conjugates	0	1	872993-05-0
10473088	Resolvin E1	HMDB	Endo	0.080467653	3	Eicosanoids	0	1	.
638015	Retinal	HMDB	Food	0.155004675	1	Prenol Lipids	0	20	116-31-4,472-86-6,7058-59-5
444795	Retinoic acid	HMDB	Food	0.053852847	2	Prenol Lipids	25	15	302-79-4,187175-63-9,56573-65-0,7005-78-9
5281877	Retinoyl b-glucuronide	HMDB	Food	0.006599905	1	Prenol Lipids	0	1	401-10-5
6440956	Retinyl beta-glucuronide	HMDB	Food	0.006800222	1	Prenol Lipids	0	0	16639-19-3
5460164	Retinyl ester	HMDB	Food	0.09082688	2	Prenol Lipids	0	1	.

PC_CID	Chemical	Dataset	Source	Concentration (µM)	No. studies	Class	PubMed	Biosys	CAS
5280531	Retinyl palmitate	NHANES	Food	0.040199529	1	Fatty Acid Esters	14	11	79-81-2,108066-99-5,37340-08-2,674786-07-3,7488-89-3
827	Ribitol	HMDB	Endo	0.460013243	1	Sugar Alcohols	0	2	488-81-3
493570	Riboflavin	HMDB	Food	0.08916203	2	Pteridines and Derivatives	19	7	83-88-5
445408	Ribothymidine	HMDB	Endo	0.200007583	1	Pyrimidine Nucleosides and Analogues	0	0	1463-10-1
5281792	Rosmarinic acid	HMDB	Food	1.380022771	1	Cinnamic Acid Derivatives	0	1	537-15-5
10253	Salicyluric acid	HMDB	Drug	1	1	Amino Acids and Derivatives	0	6	487-54-7
44257	Sapropterin	HMDB	Endo	0.010435936	2	Pteridines and Derivatives	0	2	62989-33-7
153311	Scopolamine	HMDB	Drug	0.02100069	1	Phenylacetic Acid Derivatives	0	1	51-34-3
.	Scyllitol	HMDB	Food	0.025998927	1	Cyclic Alcohols and Derivatives	.	.	.
1090	Selenite	HMDB	Endo	0.001299988	1	Non-metal Oxoanionic Compounds	0	2	14124-67-5
6326970	Selenium	HMDB	Food	1.480233729	2	Homogeneous Other Non-metal Compounds	186	0	7782-49-2,7783-07-5,11125-23-8,11133-88-3,12640-29-8,12640-30-1,12641-96-2,12733-65-2,37256-19-2,37258-85-8,37276-15-6,37368-02-8,50954-17-1,51882-60-1,95788-45-7
15104	Selenocystine	HMDB	Endo	0.073999274	1	Amino Acids and Derivatives	0	0	1464-43-3
105024	Selenomethionine	HMDB	Endo	0.689974941	1	Amino Acids and Derivatives	3	1	3211-76-5
5202	Serotonin	HMDB	Endo	0.84721566	2	Indoles	25	56	50-67-9
68617	Sertraline	HMDB	Drug	0.150002998	1	Tetralins	4	0	79617-96-2,79559-97-0
8742	Shikimic acid	HMDB	Food	0.04797861	8	Cyclic Alcohols and Derivatives	0	3	138-59-0
4082203	Silicon	HMDB	Pollut	5.979877005	1	Homogeneous Metalloid Compounds	0	0	.
104755	Silver	HMDB	Pollut	0.006300036	1	Homogeneous Transition Metal Compounds	4	0	14701-21-4
54454	Simvastatin	HMDB	Drug	0.043998488	1	Fatty Acid Esters	157	0	79902-63-9
6453725	SM(d18:1/18:0)	HMDB	Endo	774.9511538	3	Sphingolipids	0	0	58909-84-5
923	Sodium	HMDB	Food	142600.173	1	Homogeneous Alkali Metal Compounds	189	110	17341-25-2
65727	Solanidine	HMDB	Food	0.003179918	1	Steroids and Steroid Derivatives	0	0	80-78-4

PC_CID	Chemical	Dataset	Source	Concentration (µM)	No. studies	Class	PubMed	Biosys	CAS
5780	Sorbitol	HMDB	Food	3.764443344	2	Fatty Alcohols	3	6	50-70-4,15060-73-8,36134-87-9,3959-53-3,63800-20-4,75398-79-7,8013-15-8,8014-89-9,8036-93-9,8042-39-5,8045-74-7,8046-05-7
1102	Spermidine	HMDB	Endo	9.339941604	2	Alkylamines	2	26	124-20-9
1103	Spermine	HMDB	Endo	9.97019358	1	Alkylamines	0	20	71-44-3,115-04-8
3126	Sphinganine	HMDB	Endo	0.01099956	1	Sphingolipids	2	1	3102-56-5,13552-09-5,15639-50-6,73938-69-9,2304-75-8
644260	Sphinganine 1-phosphate	HMDB	Endo	0.013000077	1	Sphingolipids	0	6	19794-97-9
5280335	Sphingosine	HMDB	Endo	0.050001614	1	Sphingolipids	4	9	123-78-4,2733-29-1,475662-41-0,477243-03-1
5283560	Sphingosine 1-phosphate	HMDB	Endo	0.184833474	3	Sphingolipids	2	18	26993-30-6
638072	Squalene	HMDB	Food	1.872922415	2	Prenol Lipids	5	4	111-02-4,7683-64-9,11051-27-7,21245-10-3,94016-35-0
5281	Stearic acid	NHANES	Endo	692.0097188	1	Fatty Acids and Conjugates	9	2	57-11-4,126539-56-8,646-29-7,134503-33-6,135152-99-7,197923-10-7,294203-07-9,39390-61-9,57485-56-0,58392-66-8,609343-71-7,68937-76-8,8013-28-3,8023-06-1,8037-40-9,8037-83-0,8039-51-8,8039-52-9,8039-53-0,8039-54-1,82497-27-6,85404-83-7
5312508	Stearidonic acid	HMDB	Food	0.080798248	2	Lineolic Acids and Derivatives	0	1	20290-75-9
6426855	Stearoylcarnitine	HMDB	Food	0.056568669	2	Fatty Acid Esters	0	0	1976-27-8
27902	Stearoylethanolamide	HMDB	Food	15.000747	1	Fatty Amides	0	0	111-57-9
15559396	Stigmastanol	HMDB	Food	2.799945632	1	Steroids and Steroid Derivatives	0	0	19466-47-8
104798	Strontium	HMDB	Pollut	0.359335064	4	Homogeneous Alkaline Earth Metal Compounds	0	0	22537-39-9
10457	Suberic acid	HMDB	Endo	3.599878158	1	Carboxylic Acids and Derivatives	0	0	505-48-6
36511	Substance P	HMDB	Endo	3.59992E-06	1	Amino Acids and Derivatives	6	1	33507-63-0,11035-08-8,12769-48-1

PC_CID	Chemical	Dataset	Source	Concentration (µM)	No. studies	Class	PubMed	Biosys	CAS
160419	Succinic acid	HMDB	Endo	14.90057794	3	Carboxylic Acids and Derivatives	1	8	56-14-4
5312	Succinylacetone	HMDB	Endo	0.044157168	2	Keto-Acids and Derivatives	0	0	51568-18-4
5988	Sucrose	HMDB	Food	1.800024003	1	Disaccharides	11	14	57-50-1,8027-47-2,8030-20-4,85456-51-5,86101-30-6,87430-66-8,92004-84-7,12040-73-2,25702-74-3,100405-08-1,104242-10-6,131932-12-2,146054-35-5,146187-04-4,151756-02-4,220376-22-7,29253-78-9,29764-06-5,30027-72-6,47167-52-2,47185-09-1,47257-91-0,50857-68-6,51909-69-4,635681-90-2,64533-66-0,65545-99-5,75398-84-4,76056-38-7,78654-77-0,786702-63-4,80165-03-3
1117	Sulfate	HMDB	Endo	324.9593183	2	Non-metal Oxoanionic Compounds	0	44	14808-79-8,18785-72-3
451489	Sulfolithocholic acid	HMDB	Endo	1.599994193	1	Steroids and Steroid Derivatives	0	4	34669-57-3
443113	Sulfolithocholylglycine	HMDB	Endo	0.060000643	1	Steroids and Steroid Derivatives	0	0	15324-64-8
5350	Sulforaphane	HMDB	Food	0.539992515	1	Sulfoxides	1	1	4478-93-7
169148	Symmetric dimethylarginine	HMDB	Endo	0.5346195	3	Amino Acids and Derivatives	0	0	30344-00-4
1123	Taurine	HMDB	Endo	81.49160428	6	Sulfonic Acids and Derivatives	9	21	107-35-7,91105-79-2
387316	Taurochenodesoxycholic acid	HMDB	Endo	0.299991841	1	Steroids and Steroid Derivatives	0	12	516-35-8
6675	Taurocholic acid	HMDB	Endo	0.37999393	1	Steroids and Steroid Derivatives	0	12	81-24-3
68340	Taurocyamine	HMDB	Endo	0.367842655	2	Sulfonic Acids and Derivatives	0	1	543-18-0
2733768	Taurodeoxycholic acid	HMDB	Endo	0.062001295	1	Steroids and Steroid Derivatives	0	0	516-50-7
439763	Tauroursodeoxycholic acid	HMDB	Endo	1.999905641	1	Steroids and Steroid Derivatives	0	0	516-90-5
6013	Testosterone	HMDB	Endo	0.005345501	3	Steroids and Steroid Derivatives	493	17	58-22-0
11197	Tetracosanoic acid	NHANES	Endo	54.00086149	1	Fatty Acids and Conjugates	1	5	557-59-5
53477791	Tetradecanoylcarnitine	HMDB	Endo	0.039999033	1	Fatty Acid Esters	0	0	25597-07-3
20057343	Tetrahexosylceramide (d18:1/12:0)	HMDB	Endo	1.500052339	1	Tetrasaccharides	0	0	11034-93-8
1125	Tetrahydrobiopterin	HMDB	Endo	0.00930038	1	Pteridines and Derivatives	2	24	17528-72-2,27070-47-9
124072	Tetrahydrocurcumin	HMDB	Food	16.00018045	1	Phenols and Derivatives	0	0	36062-04-1

PC_CID	Chemical	Dataset	Source	Concentration (µM)	No. studies	Class	PubMed	Biosys	CAS
101771	Tetrahydrodeoxycorticosterone	HMDB	Endo	0.005199983	1	Steroids and Steroid Derivatives	0	1	567-03-3
5460413	Tetrahydrofolic acid	HMDB	Food	0.002499911	1	Pteridines and Derivatives	0	12	135-16-0
53480481	TG(16:0/14:0/18:1(9Z))[iso6]	HMDB	Endo	26.99900464	1	Glycerolipids	0	0	.
53480482	TG(16:0/14:0/18:2(9Z,12Z))[iso6]	HMDB	Endo	20.20035139	1	Glycerolipids	0	0	.
9543986	TG(16:0/16:0/16:1(9Z))[iso3]	HMDB	Endo	26.99900464	1	Glycerolipids	0	3	.
3246953	TG(16:0/16:0/18:0)[iso3]	HMDB	Endo	11.59994086	1	Glycerolipids	0	3	2177-98-2
53480483	TG(16:0/16:0/18:1(11Z))[iso3]	HMDB	Endo	63.59914329	1	Glycerolipids	0	0	.
25240356	TG(16:0/16:0/18:2(9Z,12Z))[iso3]	HMDB	Endo	79.79812437	1	Glycerolipids	0	0	.
25240358	TG(16:0/16:0/18:3(9Z,12Z,15Z))[iso3]	HMDB	Endo	57.09976494	1	Glycerolipids	0	0	.
9544062	TG(16:0/16:0/20:1(11Z))[iso3]	HMDB	Endo	29.60075891	1	Glycerolipids	0	3	.
9544125	TG(16:0/16:0/20:4(5Z,8Z,11Z,14Z))[iso3]	HMDB	Endo	90.90363597	1	Glycerolipids	0	3	.
9544489	TG(16:0/16:0/22:6(4Z,7Z,10Z,13Z,16Z,19Z))[iso3]	HMDB	Endo	36.49955249	1	Glycerolipids	0	0	.
9543987	TG(16:0/16:1(9Z)/16:1(9Z))[iso3]	HMDB	Endo	20.20035139	1	Glycerolipids	0	3	.
9544004	TG(16:0/16:1(9Z)/18:0)[iso6]	HMDB	Endo	63.59914329	1	Glycerolipids	0	3	.
9544011	TG(16:0/16:1(9Z)/18:1(9Z))[iso6]	HMDB	Endo	79.79812437	1	Glycerolipids	0	3	.
9544021	TG(16:0/16:1(9Z)/18:2(9Z,12Z))[iso6]	HMDB	Endo	57.09976494	1	Glycerolipids	0	3	.
9544061	TG(16:0/16:1(9Z)/20:0)[iso6]	HMDB	Endo	29.60075891	1	Glycerolipids	0	3	.
9544079	TG(16:0/16:1(9Z)/20:1(11Z))[iso6]	HMDB	Endo	139.5049382	1	Glycerolipids	0	3	.
9544150	TG(16:0/16:1(9Z)/20:4(5Z,8Z,11Z,14Z))[iso6]	HMDB	Endo	31.99885113	1	Glycerolipids	0	3	.
25240360	TG(16:0/18:0/18:1(9Z))[iso6]	HMDB	Endo	29.60075891	1	Glycerolipids	0	0	.
9544069	TG(16:0/18:0/18:2(9Z,12Z))[iso6]	HMDB	Endo	139.5049382	1	Glycerolipids	0	3	.
9544279	TG(16:0/18:0/20:4(5Z,8Z,11Z,14Z))[iso6]	HMDB	Endo	68.49768849	1	Glycerolipids	0	3	.
25240174	TG(16:0/18:1(9Z)/18:1(9Z))[iso3]	HMDB	Endo	139.5049382	1	Glycerolipids	0	0	27071-84-7
25240361	TG(16:0/18:1(9Z)/18:2(9Z,12Z))[iso6]	HMDB	Endo	214.7984185	1	Glycerolipids	0	0	.
9544199	TG(16:0/18:1(9Z)/20:1(11Z))[iso6]	HMDB	Endo	21.50101192	1	Glycerolipids	0	3	.
9544319	TG(16:0/18:1(9Z)/20:4(5Z,8Z,11Z,14Z))[iso6]	HMDB	Endo	53.59737002	1	Glycerolipids	0	3	.
25240363	TG(16:0/18:2(9Z,12Z)/18:2(9Z,12Z))[iso3]	HMDB	Endo	90.90363597	1	Glycerolipids	0	0	.
9544195	TG(16:0/18:2(9Z,12Z)/20:0)[iso6]	HMDB	Endo	21.50101192	1	Glycerolipids	0	3	.
9544231	TG(16:0/18:2(9Z,12Z)/20:1(11Z))[iso6]	HMDB	Endo	69.10312817	1	Glycerolipids	0	3	.
9544362	TG(16:0/18:2(9Z,12Z)/20:4(5Z,8Z,11Z,14Z))[iso6]	HMDB	Endo	36.49955249	1	Glycerolipids	0	3	.
53480485	TG(16:1(9Z)/14:0/18:1(11Z))[iso6]	HMDB	Endo	20.20035139	1	Glycerolipids	0	0	.

PC_CID	Chemical	Dataset	Source	Concentration (µM)	No. studies	Class	PubMed	Biosys	CAS
53480488	TG(16:1(9Z)/16:0/18:1(11Z))[iso6]	HMDB	Endo	79.79812437	1	Glycerolipids	0	0	.
53480489	TG(16:1(9Z)/16:0/18:3(9Z,12Z,15Z))[iso6]	HMDB	Endo	18.90071969	1	Glycerolipids	0	0	.
9544010	TG(16:1(9Z)/16:1(9Z)/18:0)[iso3]	HMDB	Endo	79.79812437	1	Glycerolipids	0	3	.
25240357	TG(16:1(9Z)/16:1(9Z)/18:1(9Z))[iso3]	HMDB	Endo	57.09976494	1	Glycerolipids	0	0	.
25240359	TG(16:1(9Z)/16:1(9Z)/18:2(9Z,12Z))[iso3]	HMDB	Endo	18.90071969	1	Glycerolipids	0	0	.
9544078	TG(16:1(9Z)/16:1(9Z)/20:0)[iso3]	HMDB	Endo	139.5049382	1	Glycerolipids	0	3	.
9544099	TG(16:1(9Z)/16:1(9Z)/20:1(11Z))[iso3]	HMDB	Endo	214.7984185	1	Glycerolipids	0	3	.
9544049	TG(16:1(9Z)/18:0/18:0)[iso3]	HMDB	Endo	29.60075891	1	Glycerolipids	0	3	.
53480490	TG(16:1(9Z)/18:0/18:1(11Z))[iso6]	HMDB	Endo	139.5049382	1	Glycerolipids	0	0	.
9544087	TG(16:1(9Z)/18:0/18:2(9Z,12Z))[iso6]	HMDB	Endo	214.7984185	1	Glycerolipids	0	3	.
9544200	TG(16:1(9Z)/18:0/20:1(11Z))[iso6]	HMDB	Endo	21.50101192	1	Glycerolipids	0	3	.
9544320	TG(16:1(9Z)/18:0/20:4(5Z,8Z,11Z,14Z))[iso6]	HMDB	Endo	53.59737002	1	Glycerolipids	0	3	.
9544083	TG(16:1(9Z)/18:1(9Z)/18:1(9Z))[iso3]	HMDB	Endo	214.7984185	1	Glycerolipids	0	3	.
25240364	TG(16:1(9Z)/18:1(9Z)/18:2(9Z,12Z))[iso6]	HMDB	Endo	90.90363597	1	Glycerolipids	0	0	.
9544196	TG(16:1(9Z)/18:1(9Z)/20:0)[iso6]	HMDB	Endo	21.50101192	1	Glycerolipids	0	3	.
9544232	TG(16:1(9Z)/18:1(9Z)/20:1(11Z))[iso6]	HMDB	Endo	69.10312817	1	Glycerolipids	0	3	.
9544363	TG(16:1(9Z)/18:1(9Z)/20:4(5Z,8Z,11Z,14Z))[iso6]	HMDB	Endo	36.49955249	1	Glycerolipids	0	3	.
25240366	TG(16:1(9Z)/18:2(9Z,12Z)/18:2(9Z,12Z))[iso3]	HMDB	Endo	31.99885113	1	Glycerolipids	0	0	.
9544227	TG(16:1(9Z)/18:2(9Z,12Z)/20:0)[iso6]	HMDB	Endo	69.10312817	1	Glycerolipids	0	3	.
25240372	TG(16:1(9Z)/18:2(9Z,12Z)/20:1(11Z))[iso6]	HMDB	Endo	68.49768849	1	Glycerolipids	0	0	.
9544634	TG(16:1(9Z)/20:1(11Z)/20:4(5Z,8Z,11Z,14Z))[iso6]	HMDB	Endo	23.4999901	1	Glycerolipids	0	3	.
53480491	TG(18:0/14:0/16:1(9Z))[iso6]	HMDB	Endo	26.99900464	1	Glycerolipids	0	0	.
545588	TG(18:0/14:0/18:0)[iso3]	HMDB	Endo	11.59994086	1	Glycerolipids	0	0	.
53480492	TG(18:0/14:0/18:1(11Z))[iso6]	HMDB	Endo	63.59914329	1	Glycerolipids	0	0	.
53480494	TG(18:0/14:0/18:2(9Z,12Z))[iso6]	HMDB	Endo	79.79812437	1	Glycerolipids	0	0	.
53480495	TG(18:0/16:0/18:1(11Z))[iso6]	HMDB	Endo	29.60075891	1	Glycerolipids	0	0	.
53480496	TG(18:0/16:0/18:3(9Z,12Z,15Z))[iso6]	HMDB	Endo	214.7984185	1	Glycerolipids	0	0	.
25240368	TG(18:0/18:0/18:2(9Z,12Z))[iso3]	HMDB	Endo	21.50101192	1	Glycerolipids	0	0	.
9544216	TG(18:0/18:1(9Z)/18:2(9Z,12Z))[iso6]	HMDB	Endo	69.10312817	1	Glycerolipids	0	3	.
25240370	TG(18:0/18:2(9Z,12Z)/18:2(9Z,12Z))[iso3]	HMDB	Endo	68.49768849	1	Glycerolipids	0	0	.
9544638	TG(18:0/18:2(9Z,12Z)/20:4(5Z,8Z,11Z,14Z))[iso6]	HMDB	Endo	23.4999901	1	Glycerolipids	0	3	.

PC_CID	Chemical	Dataset	Source	Concentration (µM)	No. studies	Class	PubMed	Biosys	CAS
53480498	TG(18:1(11Z)/14:0/18:1(11Z))[iso3]	HMDB	Endo	79.79812437	1	Glycerolipids	0	0	.
53480500	TG(18:1(11Z)/14:0/18:2(9Z,12Z))[iso3]	HMDB	Endo	57.09976494	1	Glycerolipids	0	0	.
53480501	TG(18:1(11Z)/16:0/18:1(11Z))[iso3]	HMDB	Endo	139.5049382	1	Glycerolipids	0	0	.
53480503	TG(18:1(11Z)/16:0/18:2(9Z,12Z))[iso3]	HMDB	Endo	214.7984185	1	Glycerolipids	0	0	.
53480504	TG(18:1(11Z)/16:0/18:3(9Z,12Z,15Z))[iso6]	HMDB	Endo	90.90363597	1	Glycerolipids	0	0	.
53480505	TG(18:1(11Z)/16:0/20:4(5Z,8Z,11Z,14Z))[iso6]	HMDB	Endo	53.59737002	1	Glycerolipids	0	0	.
53480506	TG(18:1(11Z)/16:1(9Z)/18:1(11Z))[iso3]	HMDB	Endo	214.7984185	1	Glycerolipids	0	0	.
53480508	TG(18:1(11Z)/16:1(9Z)/18:2(9Z,12Z))[iso6]	HMDB	Endo	90.90363597	1	Glycerolipids	0	0	.
53480509	TG(18:1(11Z)/18:0/18:1(11Z))[iso3]	HMDB	Endo	21.50101192	1	Glycerolipids	0	0	.
53480511	TG(18:1(11Z)/18:0/18:2(9Z,12Z))[iso6]	HMDB	Endo	69.10312817	1	Glycerolipids	0	0	.
53480512	TG(18:1(9Z)/14:0/18:1(9Z))[iso3]	HMDB	Endo	79.79812437	1	Glycerolipids	0	0	.
53480514	TG(18:1(9Z)/14:0/20:4(5Z,8Z,11Z,14Z))[iso6]	HMDB	Endo	31.99885113	1	Glycerolipids	0	0	.
53480515	TG(18:1(9Z)/16:0/18:3(9Z,12Z,15Z))[iso6]	HMDB	Endo	90.90363597	1	Glycerolipids	0	0	.
53480516	TG(18:1(9Z)/16:0/20:2(11Z,14Z))[iso6]	HMDB	Endo	69.10312817	1	Glycerolipids	0	0	.
53480517	TG(18:1(9Z)/16:0/20:5(5Z,8Z,11Z,14Z,17Z))[iso6]	HMDB	Endo	36.49955249	1	Glycerolipids	0	0	.
53480518	TG(18:1(9Z)/16:0/22:5(7Z,10Z,13Z,16Z,19Z))[iso6]	HMDB	Endo	23.4999901	1	Glycerolipids	0	0	.
53480520	TG(18:1(9Z)/18:0/18:3(9Z,12Z,15Z))[iso6]	HMDB	Endo	68.49768849	1	Glycerolipids	0	0	.
53480521	TG(18:1(9Z)/18:1(11Z)/18:1(9Z))[iso3]	HMDB	Endo	69.10312817	1	Glycerolipids	0	0	.
53480522	TG(18:1(9Z)/18:1(11Z)/18:2(9Z,12Z))[iso6]	HMDB	Endo	68.49768849	1	Glycerolipids	0	0	.
53480523	TG(18:1(9Z)/18:1(9Z)/18:3(6Z,9Z,12Z))[iso6]	HMDB	Endo	53.59737002	1	Glycerolipids	0	0	.
9544639	TG(18:1(9Z)/18:1(9Z)/20:4(5Z,8Z,11Z,14Z))[iso3]	HMDB	Endo	23.4999901	1	Glycerolipids	0	3	.
25240373	TG(18:1(9Z)/18:2(9Z,12Z)/18:2(9Z,12Z))[iso3]	HMDB	Endo	53.59737002	1	Glycerolipids	0	0	.
53480526	TG(18:2(9Z,12Z)/14:0/18:2(9Z,12Z))[iso3]	HMDB	Endo	18.90071969	1	Glycerolipids	0	0	.
53480529	TG(18:2(9Z,12Z)/16:0/18:3(9Z,12Z,15Z))[iso6]	HMDB	Endo	31.99885113	1	Glycerolipids	0	0	.
53480530	TG(18:2(9Z,12Z)/16:0/20:2(11Z,14Z))[iso6]	HMDB	Endo	68.49768849	1	Glycerolipids	0	0	.
53480533	TG(18:2(9Z,12Z)/18:0/18:3(9Z,12Z,15Z))[iso6]	HMDB	Endo	53.59737002	1	Glycerolipids	0	0	.
53480537	TG(18:2(9Z,12Z)/18:1(9Z)/18:3(6Z,9Z,12Z))[iso6]	HMDB	Endo	36.49955249	1	Glycerolipids	0	0	.
53480541	TG(18:2(9Z,12Z)/18:1(9Z)/20:3(8Z,11Z,14Z))[iso6]	HMDB	Endo	23.4999901	1	Glycerolipids	0	0	.
5322095	TG(18:2(9Z,12Z)/18:2(9Z,12Z)/18:2(9Z,12Z))	HMDB	Endo	36.49955249	1	Glycerolipids	0	3	537-40-6
9544625	TG(18:2(9Z,12Z)/18:2(9Z,12Z)/20:2(11Z,14Z))[iso3]	HMDB	Endo	23.4999901	1	Glycerolipids	0	0	.
53480549	TG(18:3(9Z,12Z,15Z)/18:0/18:3(9Z,12Z,15Z))[iso3]	HMDB	Endo	36.49955249	1	Glycerolipids	0	0	.

PC_CID	Chemical	Dataset	Source	Concentration (µM)	No. studies	Class	PubMed	Biosys	CAS
9545280	TG(20:0/20:1(11Z)/20:4(5Z,8Z,11Z,14Z))[iso6]	HMDB	Endo	0.009999702	1	Glycerolipids	0	3	.
5429	Theobromine	HMDB	Food	1.099988802	1	Imidazopyrimidines	0	2	83-67-0
2153	Theophylline	HMDB	Food	29.00012093	1	Imidazopyrimidines	15	2	58-55-9,111079-49-3,46157-00-0,56645-32-0,75448-53-2
1130	Thiamine	HMDB	Food	0.107335053	2	Thiamines	0	10	67-03-8,100660-17-1,115461-66-0,55463-15-5,57777-32-9,70-16-6
9322	Thiocyanate	HMDB	Endo	42.84547335	2	Thiocyanates	11	2	302-04-5,71048-69-6
1084	Thiosulfate	HMDB	Endo	12.29631962	2	Non-metal Oxoanionic Compounds	0	5	14383-50-7
5280497	Thromboxane A2	HMDB	Endo	0.000239993	1	Eicosanoids	12	26	57576-52-0
5283137	Thromboxane B2	HMDB	Endo	0.016324308	10	Eicosanoids	36	7	54397-85-2
6438711	Thromboxane B3	HMDB	Endo	1.7E-05	1	Eicosanoids	0	0	71953-80-5
5789	Thymidine	HMDB	Endo	0.204947793	2	Pyrimidine Nucleosides and Analogues	14	11	50-89-5,50-88-4,157049-39-3,35902-13-7
6989	Thymol	HMDB	Drug	0.609997756	1	Prenol Lipids	0	0	89-83-8
5819	Thyroxine	HMDB	Endo	1.78877E-05	2	Amino Acids and Derivatives	146	18	51-48-9,24486-40-6,587-29-1,587-30-4,7200-84-2,74-16-8,55-03-8,25416-65-3
44237187	Thyroxine sulfate	HMDB	Endo	1.99756E-05	2	Amino Acids and Derivatives	0	1	77074-49-8
22833596	Tiglylcarnitine	HMDB	Endo	0.040619786	2	Fatty Acid Esters	0	0	64191-86-2
5352426	Tin	HMDB	Pollut	0.013000077	1	Homogeneous Post-transition Metal Compounds	3	0	7440-31-5
114942	Titanium	HMDB	Pollut	0.001499985	1	Homogeneous Transition Metal Compounds	0	0	16043-45-1,77889-68-0,22541-75-9
1140	Toluene	NHANES	Pollut	0.00146032	3	Aromatic Homomonocyclic Compounds	1	1	108-88-3
5284627	Topiramate	HMDB	Drug	1.470055272	1	Dioxolopyrans	1	0	97240-79-4
445858	trans-Ferulic acid	HMDB	Food	0.049075272	9	Cinnamic Acid Derivatives	0	1	537-98-4
12313421	trans-Nonachlor	NHANES	Pollut	0.038300206	3	Pesticides	1	0	39765-80-5,5103-73-1,24143-69-9,29555-44-0
5558	Tribromomethane	NHANES	Pollut	6.21217E-06	1	Organobromides	0	0	75-25-2
6212	Trichloromethane	NHANES	Pollut	0.000100174	3	Organochlorides	0	0	67-66-3,8013-54-5
17085	Tricosanoic acid	HMDB	Endo	0.033001575	1	Fatty Acids and Conjugates	0	0	2433-96-7

PC_CID	Chemical	Dataset	Source	Concentration (µM)	No. studies	Class	PubMed	Biosys	CAS
53477785	Trihexosylceramide (d18:1/12:0)	HMDB	Endo	1.999905641	1	Sphingolipids	0	0	71965-57-6
20057314	Trihexosylceramide (d18:1/9Z-18:1)	HMDB	Endo	1.699951974	1	Sphingolipids	0	0	71965-57-6
1146	Trimethylamine	HMDB	Food	0.420000238	1	Alkylamines	1	5	75-50-3,13960-80-0,19530-21-3,4558-12-7
1145	Trimethylamine N-oxide	HMDB	Food	37.79965592	1	Organic Oxoazanium Compounds	1	4	1184-78-7
23964	Tungsten	HMDB	Pollut	0.000149921	3	Homogeneous Transition Metal Compounds	0	1	7440-33-7
5610	Tyramine	HMDB	Endo	0.002157511	2	Phenethylamines	0	8	51-67-2
4462	Ubiquinone-1	HMDB	Endo	0.00312007	1	Benzoquinones	0	12	727-81-1
1174	Uracil	HMDB	Endo	0.597500595	2	Diazines	5	23	66-22-8,51953-14-1,51953-19-6,766-19-8,138285-60-6,144104-68-7,153445-42-2,16908-84-2,24897-50-5,42910-77-0,4433-21-0,4433-24-3,66224-60-0,66255-05-8
1176	Urea	HMDB	Endo	5175.027851	4	Ureas	57	24	57-13-6,173144-80-4,173994-65-5,174693-33-5,175276-38-7,30535-50-3,37955-36-5,4744-36-9,860639-56-1
1175	Uric acid	HMDB	Endo	317.0628439	8	Imidazopyrimidines	569	18	69-93-2,13154-20-6,33278-42-1,34318-07-5,42911-25-1,42911-27-3,42911-28-4,530-13-2
6029	Uridine	HMDB	Endo	3.109929322	2	Pyrimidine Nucleosides and Analogues	1	15	58-96-8,12693-39-9,21231-59-4
6031	Uridine 5'-diphosphate	HMDB	Endo	41.00114528	1	Pyrimidine Nucleotides	0	69	58-98-0
6030	Uridine 5'-monophosphate	HMDB	Endo	183.9934207	1	Pyrimidine Nucleotides	0	60	58-97-9,27416-86-0,53624-79-6,81795-92-8
8629	Uridine diphosphate glucose	HMDB	Endo	154.9961069	1	Pyrimidine Nucleotides	0	9	133-89-1
736715	Urocanic acid	HMDB	Endo	0.42998713	1	Azoles	0	5	104-98-3
72424	Uroporphyrin I	HMDB	Endo	0.012000584	1	Tetrapyrroles and Derivatives	0	1	607-14-7
31401	Ursodeoxycholic acid	HMDB	Endo	0.159997034	1	Steroids and Steroid Derivatives	9	0	128-13-2,50809-41-1,80225-86-1
21252318	Ursodeoxycholic acid 3-sulfate	HMDB	Endo	2.68693194	2	Steroids and Steroid Derivatives	0	0	68780-73-4
7991	Valeric acid	HMDB	Food	0.600015374	1	Fatty Acids and Conjugates	0	0	109-52-4

PC_CID	Chemical	Dataset	Source	Concentration (μM)	No. studies	Class	PubMed	Biosys	CAS
53481619	Valerylcarnitine	HMDB	Food	0.1400018	1	Fatty Acid Esters	0	0	.
3121	Valproic acid	HMDB	Drug	207.9920796	1	Fatty Acids and Conjugates	10	2	99-66-1
23990	Vanadium	HMDB	Pollut	0.000884401	3	Homogeneous Transition Metal Compounds	1	0	7440-62-2,22537-31-1,13966-93-3,195161-77-4,22541-77-1,24763-58-4
8468	Vanillic acid	HMDB	Food	0.41765481	9	Benzoic Acid and Derivatives	0	0	121-34-6
1183	Vanillin	HMDB	Food	6.387225627	8	Phenols and Derivatives	0	2	121-33-5
1245	Vanillylmandelic acid	HMDB	Endo	0.035000253	1	Phenols and Derivatives	2	2	55-10-7,2394-20-9
10805	Vanylglycol	HMDB	Endo	0.020795888	2	Phenols and Derivatives	3	3	534-82-7,67423-45-4
8230	Vasopressin	HMDB	Endo	5.30002E-06	1	Peptides	12	2	113-79-1
5656	Venlafaxine	HMDB	Drug	1.999905641	1	Phenols and Derivatives	4	0	93413-69-5,93413-44-6
445354	Vitamin A	NHANES	Food	1.909611847	1	Prenol Lipids	193	19	68-26-8,11103-57-4,13123-33-6,1341-18-0,1406-67-3,17104-91-5,53637-36-8,5979-23-7
54670067	Vitamin C	NHANES	Food	56.09799095	1	Dihydrofurans	189	18	50-81-7,89924-69-6,129940-97-2,14536-17-5,154170-90-8,259133-78-3,30208-61-8,50976-75-5,56172-55-5,56533-05-2,57304-74-2,57606-40-3,623158-95-2,6730-29-6,882690-91-7,884381-69-5,885512-24-3,88845-26-5,53262-66-1
5280795	Vitamin D3	HMDB	Food	0.020213593	2	Prenol Lipids	461	13	67-97-0,1406-16-2
5280483	Vitamin K1	HMDB	Food	0.002525794	2	Prenol Lipids	45	3	84-80-0,12001-79-5,10485-69-5,15973-57-6,27696-10-2,50926-17-5,11104-38-4
91577	Vitamin K1 2,3-epoxide	HMDB	Food	2.09994E-06	1	Prenol Lipids	0	5	25486-55-9
1188	Xanthine	HMDB	Endo	0.994813497	7	Imidazopyrimidines	0	15	69-89-6,16819-86-6,51953-26-5,28522-58-9,33669-67-9,42911-15-9,6050-36-8
1189	Xanthosine	HMDB	Endo	5.079942791	1	Purine Nucleosides and Analogues	0	4	146-80-5
5699	Xanthurenic acid	HMDB	Endo	0.022000282	1	Amino Acids and Derivatives	0	2	59-00-7

PC_CID	Chemical	Dataset	Source	Concentration (µM)	No. studies	Class	PubMed	Biosys	CAS
5280899	Zeaxanthin	HMDB	Food	0.035778791	2	Prenol Lipids	14	2	144-68-3
32051	Zinc (II) ion	HMDB	Food	6.454644828	6	Homogeneous Transition Metal Compounds	0	133	23713-49-7
115139	Zirconium	HMDB	Pollut	0.002634926	2	Homogeneous Transition Metal Compounds	0	0	7440-67-7
